# A supermatrix analysis of genomic, morphological, and paleontological data from crown Cetacea

**DOI:** 10.1186/1471-2148-11-112

**Published:** 2011-04-25

**Authors:** Jonathan H Geisler, Michael R McGowen, Guang Yang, John Gatesy

**Affiliations:** 1 Department of Anatomy, New York College of Osteopathic Medicine, New York Institute of Technology, Northern Boulevard, Old Westbury, NY,11568, USA; 2 Department of Biology, Spieth Hall, University of California, Riverside, CA, 92521, US; 3 Center for Molecular Medicine and Genetics, Wayne State University School of Medicine, 540 E. Canfield St., Detroit, MI, 48201, USA; 4 Jiangsu Key Laboratory for Biodiversity and Biotechnology, College of Life Sciences, Nanjing Normal University, Nanjing 210046, China

## Abstract

**Background:**

Cetacea (dolphins, porpoises, and whales) is a clade of aquatic species that includes the most massive, deepest diving, and largest brained mammals. Understanding the temporal pattern of diversification in the group as well as the evolution of cetacean anatomy and behavior requires a robust and well-resolved phylogenetic hypothesis. Although a large body of molecular data has accumulated over the past 20 years, DNA sequences of cetaceans have not been directly integrated with the rich, cetacean fossil record to reconcile discrepancies among molecular and morphological characters.

**Results:**

We combined new nuclear DNA sequences, including segments of six genes (~2800 basepairs) from the functionally extinct Yangtze River dolphin, with an expanded morphological matrix and published genomic data. Diverse analyses of these data resolved the relationships of 74 taxa that represent all extant families and 11 extinct families of Cetacea. The resulting supermatrix (61,155 characters) and its sub-partitions were analyzed using parsimony methods. Bayesian and maximum likelihood (ML) searches were conducted on the molecular partition, and a molecular scaffold obtained from these searches was used to constrain a parsimony search of the morphological partition. Based on analysis of the supermatrix and model-based analyses of the molecular partition, we found overwhelming support for 15 extant clades. When extinct taxa are included, we recovered trees that are significantly correlated with the fossil record. These trees were used to reconstruct the timing of cetacean diversification and the evolution of characters shared by "river dolphins," a non-monophyletic set of species according to all of our phylogenetic analyses.

**Conclusions:**

The parsimony analysis of the supermatrix and the analysis of morphology constrained to fit the ML/Bayesian molecular tree yielded broadly congruent phylogenetic hypotheses. In trees from both analyses, all Oligocene taxa included in our study fell outside crown Mysticeti and crown Odontoceti, suggesting that these two clades radiated in the late Oligocene or later, contra some recent molecular clock studies. Our trees also imply that many character states shared by river dolphins evolved in their oceanic ancestors, contradicting the hypothesis that these characters are convergent adaptations to fluvial habitats.

## Background

**Figure 1:**
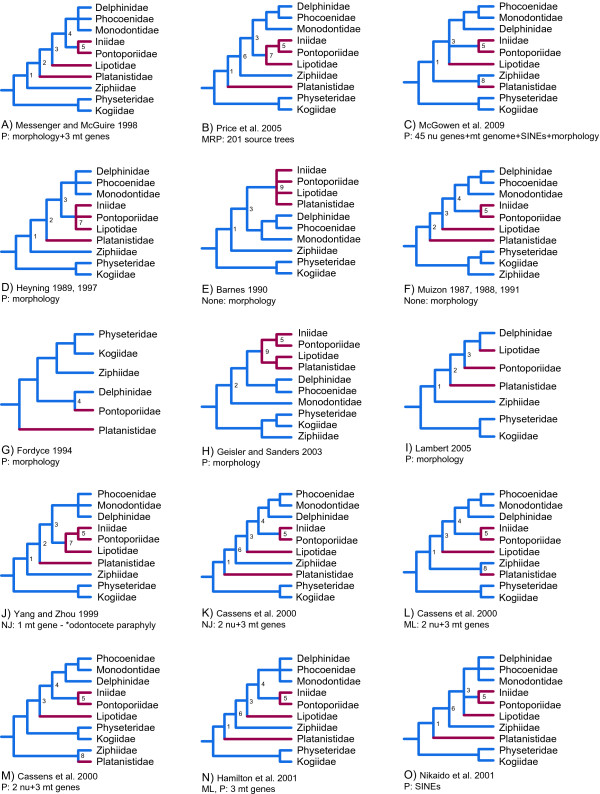
**Previous hypotheses that position extant river dolphins, including *Pontoporia*, relative to other living odontocete lineages. Continued in Figure 2**. Topologies based on combined analysis of morphology and molecules (A-C), morphology (D-I), and molecules (J-O) are shown. River dolphin lineages are colored red, and other branches are blue. Groupings that are commonly replicated in the various trees are labeled 1-9. For each topology, the following are shown: authors, date of publication, mode of analysis (P = parsimony, ML = maximum likelihood, NJ = neighbor joining distance, Bayes = Bayesian analysis, MRP = matrix representation with parsimony supertree, none = tree constructed manually), and data examined (morphology, mitochondrial [mt] genes, nuclear [nu] genes, mt genomes, source trees = published topologies used as input for MRP, SINEs = insertions of short interspersed nu elements). In the analysis of Yang and Zhou [12], Odontoceti was not supported as monophyletic (J).

**Figure 2:**
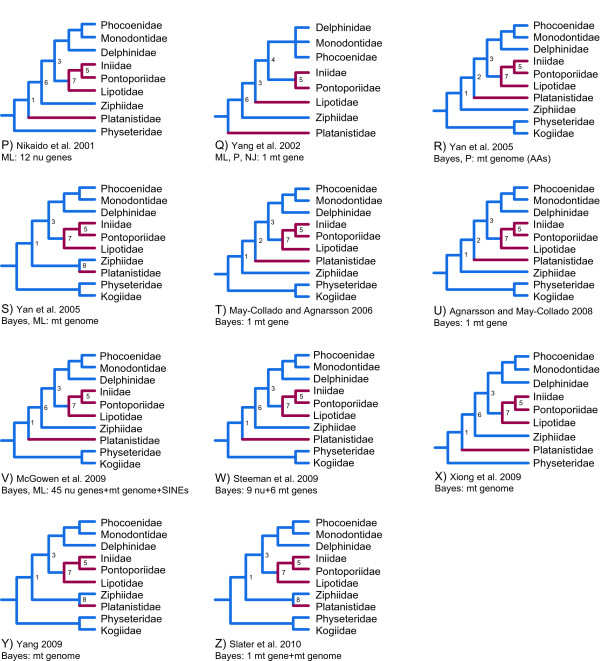
**Previous hypotheses that position extant river dolphins, including *Pontoporia*, relative to other living odontocete lineages. Continued from Figure 1**. Topologies based on molecules (P-Z) are shown. River dolphin lineages are colored red, and other branches are blue. Groupings that are commonly replicated in the various trees are labeled 1-9. For each topology, the following are shown: authors, date of publication, mode of analysis (ML = maximum likelihood, NJ = neighbor joining distance, Bayes = Bayesian analysis), and data examined (mitochondrial [mt] genes, nuclear [nu] genes, mt genomes, AAs = amino acids).

It has been 12 years since the publication of Messenger and McGuire [1], the first major effort to develop a phylogenetic hypothesis for crown Cetacea (Neoceti) based on a combined phylogenetic analysis of morphological and molecular characters (Figure 1A). Since that time, the amount of molecular data published on cetaceans has increased by more than two orders of magnitude, the number of relevant morphological characters has increased ~50%, while advances in computer applications and analytical methods now enable large-scale phylogenetic analyses that could not be completed in 1998. Although the Messenger and McGuire [1] study was groundbreaking, some of their morphological characters and observations have been disputed [2]. In addition, the only extinct cetacean included in their study was a composite outgroup taxon, Archaeoceti, despite the fact that Cetacea has a rich fossil record [3]. Given these developments and the wide range of topologies supported by subsequent morphological [4-11] (Figure 1D-I), molecular [12-24] (Figure 1J-O, Figure 2P-Z), and combined analyses [20,25] (Figure 1B-C), a second look at cetacean phylogeny using a concatenation of morphological and molecular characters from both living and extinct taxa is long overdue.

In the absence of a robust phylogenetic hypothesis for Cetacea that includes extant and extinct taxa, molecular systematists have used DNA-based clocks to time branching events within Cetacea (e.g, [24]). To date, these molecular clock studies have produced estimates for speciation events that vary widely. For example, Cassens et al. [13] suggested that the split between Kogiidae (pygmy and dwarf sperm whales) and Physeteridae (giant sperm whale) occurred approximately 37 Ma (million years ago) whereas recent dating analyses produced much younger estimates, from means of 22 Ma [21] to 24 Ma [20]. Many calibration points in molecular clock studies of Cetacea have been based on extinct taxa that have not been included in rigorous phylogenetic analyses of character matrices, which may explain in part the wide range of published divergence dates. In these cases, molecular systematists have had to trust the opinions of paleontologists regarding relationships of these extinct taxa to extant cetaceans [20-22,24]. A reliance on expert opinions is understandable given the absence of rigorous phylogenetic studies of fossils. However, a more comprehensive phylogenetic hypothesis that directly combines molecular data and fossils is required to rigorously estimate the timing of cetacean diversification, to test divergence times based on molecular clocks, and also to develop more reliable calibration points for subsequent molecular clock studies.

Messenger and McGuire [1] focused on the apparent conflict between molecular data, which at the time supported paraphyly of Odontoceti [26,27], and morphological data, which strongly supported odontocete monophyly [5,28]. Since 1998, additional morphological support for Odontoceti has been presented [2], while the balance of molecular studies, in particular insertions of transposons [15,29], mitochondrial (mt) genomes (e.g., [30]), and nuclear (nu) DNA sequences (e.g., [31]), now support odontocete monophyly. Messenger and McGuire [1] found relatively low bootstrap support for most higher-level clades within Odontoceti, including nodes defining the branching sequence of taxa collectively referred to as "river dolphins." River dolphins include odontocetes that share long, narrow rostra, an elongate and fused mandibular symphysis, and numerous teeth in the upper and lower jaws [32]. These taxa also are characterized by a flexible neck, broad forelimb flippers, and eyes that are reduced relative to most extant cetaceans [13,33]. Four of these species are restricted to rivers; Ganges and Indus River dolphins (*Platanista gangetica*, *P. minor*), the functionally extinct Yangtze River dolphin (*Lipotes vexillifer*), and the Amazon River dolphin (*Inia geoffrensis*); whereas one, the franciscana (*Pontoporia blainvillei*), occurs in coastal/estuarine waters off of Eastern South America. Although molecular data for *Lipotes *and *Pontoporia *were not available at the time of the Messenger and McGuire [1] study, subsequently published DNA sequences for these two taxa [13,17], as well as new sequences for *Platanista *and *Inia *[14,20,30], have not led to a consensus on river dolphin relationships (Figures 1, 2). A synthesis of these diverse data and new character evidence are necessary to determine which signals emerge as the strongest in combined analysis of all relevant phylogenetic data.

Skeletal similarities among river dolphins were long thought to be evidence of their monophyly [6,34], although the presence of a vestibular sac off the nasal passage [4] and some basicranial sinus features [35] ally *Lipotes*, *Inia*, and in some cases *Pontoporia *with Delphinoidea, the clade that includes porpoises and oceanic dolphins. If extant river dolphins are monophyletic, and if their affinity for freshwater is an ancestral trait, then their far-flung distribution can be explained by river hopping, analogous to the widely recognized biogeographic process of island hopping [2]. However, this scenario is now unwarranted given that recent molecular data strongly support river dolphin paraphyly or polyphyly [13-15,17,20,21]. Instead, Hamilton et al. [14] suggested that Cenozoic changes in sea level essentially stranded the ancestors of extant river dolphins in different river systems, where they subsequently developed intolerance to salt water on at least three occasions. It also has been suggested that the scarcity of close extant relatives to river dolphins in the oceans is the result of past competition with extinct members of Delphinoidea in the marine environment [13,15]. In developing these scenarios, molecular workers frequently referred to extinct taxa thought to be close relatives of extant river dolphins; however, their hypotheses were seriously hampered by the fact that there is still no published phylogenetic hypothesis based on molecular and morphological characters that includes extensive sampling of both extant and extinct odontocete taxa. Until such a joint study is completed, hypotheses that explain the distribution of extant river dolphins will remain highly speculative.

The main objectives of the current study are: 1) to derive a robust phylogenetic hypothesis for crown Cetacea that is based on a supermatrix analysis of both genomic and paleontological data, 2) to allocate, for the first time, many extinct crown cetaceans to clades with extant members in the context of molecular data, and to discuss the temporal implications of these allocations for the radiation of crown Odontoceti and crown Mysticeti, and 3) to use our integrated supermatrix analysis of molecules, morphology, and fossils to reconstruct the biogeographic history of river dolphins and the evolution of skeletal features shared by these species. Our combined dataset merges published data with newly generated morphological and molecular characters, including six nu gene fragments (~2,800 basepairs) for the Yangtze River dolphin, a species that has been difficult to place in previous systematic studies. Unlike previous systematic studies that have sampled nearly all extant species of Cetacea [18-21,24,25], the present study takes a different approach. Speciose extant families are represented by multiple taxa, all other extant families are represented by at least one species, and nearly all extinct families of crown Cetacea are sampled for at least one exemplar (Table 1).

**Table 1:** Classification of named taxa and operational taxonomic units included in phylogenetic analyses.

Outgroups	
	*Sus scrofa*

	*Bos taurus*

	Hippopotamidae^

**Cetacea**	

	*†Georgiacetus vogtlensis*

	*†Zygorhiza kochii*

**Neoceti**	

**Odontoceti**	

	†ChM PV2761

	†ChM PV2764

	†ChM PV4178

	†ChM PV4802

	†ChM PV4961

	†ChM PV5852

	*†Archaeodelphis patrius*

	*†Agorophius pygmaeus*

	*†Simocetus rayi*

	*†Patriocetus kazakhstanicus*

	*†Prosqualodon davidis*

	*†Squaloziphius emlongi*

	**†Xenorophidae**

	†ChM PV2758

	†ChM PV4746

	†ChM PV4834

	†ChM PV5711

	*†Xenorophus sloanii*

	*†Xenorophus *sp.

	**†Waipatiidae**

	*†Waipatia maerewhenua*

	**†Squalodontidae**

	*†Squalodon calvertensis*

	**Physeteroidea**

	*†Orycterocetus crocodilinus*

	**Physeteridae**

	*Physeter macrocephalus*

	**Kogiidae**

	*Kogia^*

Synrhina*	

	**†Eurhinodelphinidae**

	*Xiphiacetus bossi*

	**Ziphiidae**

	*†Ninoziphius platyrostris*

	*Berardius*^

	*Tasmacetus shepherdi*

	*Ziphius cavirostris*

	*Mesoplodon^*

	**Platanistoidea**

	**†Squalodelphinidae**

	*†Notocetus vanbenedeni*

	**†Platanistidae**

	*†Zarhachis flagellator*

	*Platanista^*

**Delphinida**	

	*†Atocetus nasalis*

	**†Kentriodontidae**

	*†Kentriodon pernix*

	**Lipotidae**

	*Lipotes vexillifer*

	*†Parapontoporia wilsoni*

	*†Parapontoporia sternbergi*

	**Inioidea**

	*†Brachydelphis mazeasi*

	*†Pliopontos littoralis*

	**Iniidae**

	*Inia geoffrensis*

	**Pontoporiidae**

	*Pontoporia blainvillei*

	**Delphinoidea**

	**†Albireonidae**

	*†Albireo whistleri*

	**Delphinidae**

	*Orcinus orca*

	*Orcaella brevirostris*

	*Leucopleurus acutus*

	**Delphininae**

	*Delphinus^*

	*Tursiops truncatus*

	**Globicephalinae**

	*Globicephala^*

	*Pseudorca crassidens*

	*Grampus griseus*

	Monodontoidae*

	Monodontidae^

	**Phocoenidae**

	*Phocoena phocoena*

	*Phocoenoides dalli*

**Mysticeti**	

	†ChM PV4745

	†ChM PV5720

	**†Mammalodontidae**

	*†Mammalodon colliveri*

	*†Janjucetus hunderi*

	**†Aetiocetidae**

	*†Aetiocetus cotylalveus*

	*†Chonecetus goedertorum*

**Chaeomysticeti**	

	**†Eomysticetoidea**

	**†Cetotheriopsidae**

	*†Micromysticetus rothauseni*

	**†Eomysticetidae**

	*†Eomysticetus whitmorei*

	**Balaenomorpha**

	*†Diorocetus hiatus*

	*†Pelocetus calvertensis*

	Balaenidae^

	Plicogulae*

	*Caperea marginata*

	**Balaenopteroidea**

	**Balaenopteridae**

	*†Parabalaenoptera baulinensis*

	*Balaenoptera physalus*

	*Megaptera novaeangliae*

	**Eschrichtiidae**

	*Eschrichtius robustus*

Taxa and OTUs included in the phylogenetic analysis are unbolded. * Taxa named in the present study (see Appendix). ^ Taxa assumed to be monophyletic in analyses. † Extinct.

## Results

### Phylogenetic Hypotheses Based on Fossils and Molecules

The primary focus of this study was to produce phylogenetic hypotheses for crown group Cetacea that incorporate extensive character information from both fossils and molecules. First, we executed separate analyses of morphological and molecular datasets to record phylogenetic patterns. Then, we analyzed the combined database in a parsimony supermatrix context and executed an analysis of the morphological data constrained to fit the ML/Bayesian molecular tree.

**Figure 3:**
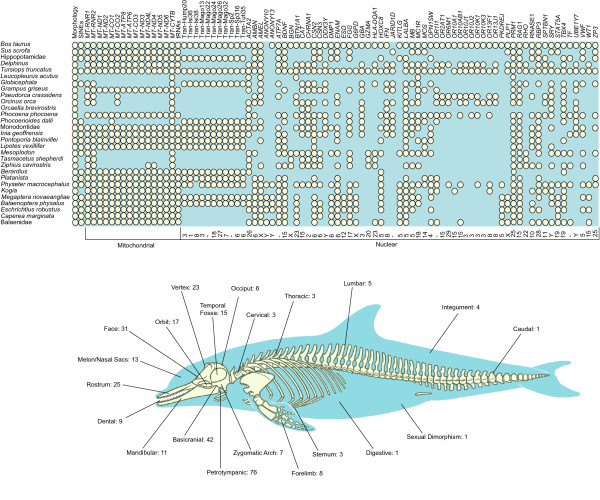
**Characters in our supermatrix of Cetacea**. Datasets (top) sampled for each of the 29 extant taxa in the analysis (left) are indicated by cream colored circles (see Methods for composition of composite operational taxonomic units). The chromosomal positions of most nuclear loci in the domestic cow genome are given (29 autosomes, X and Y sex chromosomes, "-" = not mapped). "Tran-" indicates sequences that flank SINE insertions [15]. The 45 extinct taxa (Table 1) were coded for morphology only. The anatomical positions of the 304 morphological characters in the supermatrix are shown at the bottom of the figure.

The morphological dataset includes 304 characters with a focus on variation in the skull region (Figure 3). Parsimony analysis of the morphology partition yielded four minimum length trees, each 1743.78 steps in length (Additional file 1: Fig. S1). In describing the results of our analyses, we use an unranked classification scheme that is new to this study (Table 1), but heavily influenced by several previous phylogenetic hypotheses and classifications [2,3,36,37]. As in an analysis of an earlier version of the morphological partition [2], monophyly of Mysticeti, Odontoceti, Inioidea, and Physteridae + Kogiidae (Physeteroidea) was supported. However, unlike that earlier work, Delphinoidea and Inioidea + Delphinoidea also were supported, as in many molecular studies [15,17,18]. Our greater taxonomic sampling of delphinidans, as compared to that of Geisler and Sanders [2], allowed us to test several traditional families and subfamilies of Cetacea. We found support for monophyly of Delphinidae, Phocoenidae, and Delphininae. Although many nodes are shared among the trees supported by morphology and those favored by molecules, areas of disagreement remain. The morphological partition supported several groupings found by previous morphological studies but contradicted by most molecular studies, including Balaenoidea [2,38,39], Physeteroidea + Ziphiidae [2,9,10], *Platanista *+ *Lipotes *[2], and the grouping of *Orcinus orca *within Globicephalinae [6]. We also found morphological support for two novel groupings, Balaenoidea + Balaenopteridae and Monodontidae + Delphinidae. A parsimony analysis with implied weighting of characters (Additional file 1: Fig. S2; [40]) and a Bayesian analysis (Additional file 1: Fig. S3) produced broadly similar topologies. These additional analyses of the morphological partition support monophyly of Mysticeti, Balaenoidea, Odontoceti, Physeteroidea, Ziphiidae, Inioidea, Delphinoidea, and Inioidea + Delphinoidea. Most notably, a clade including all extant odontocetes, but excluding all Oligocene taxa, was supported by a posterior probability (PP) of 0.96. Most higher-level relationships within the odontocete crown group were not well supported in the Bayesian analysis (PP < 0.95), and as in the parsimony analysis with implied weights, Monodontidae fell among the delphinids as the sister-group to *Orcaella *(Additional file 1: Figs. S2, S3).

**Figure 4:**
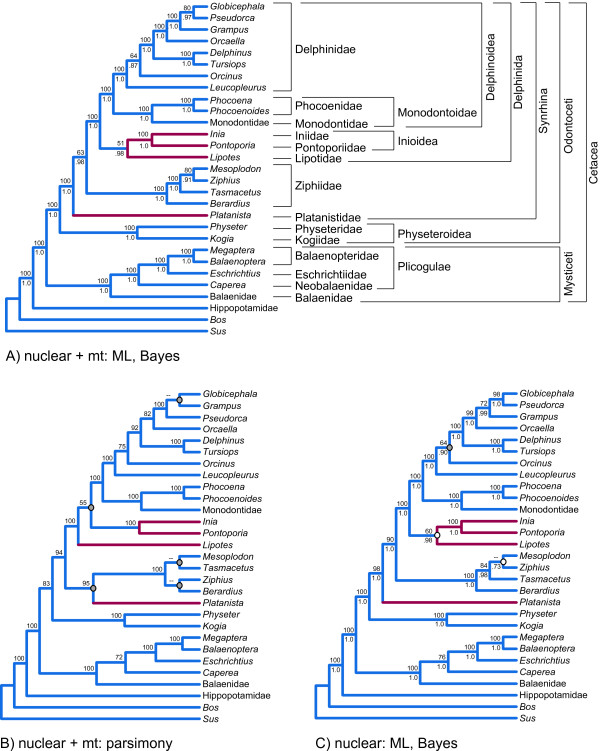
**Topologies supported by analyses of molecular data in the supermatrix**. ML/Bayesian tree for all molecular data (A) parsimony tree for all molecular data (B), and ML/Bayesian tree for the nuclear data (C) are shown. Bootstrap scores >50% are above internodes, and Bayesian posterior probabilities >0.50 are below internodes. In B, gray circles at nodes indicate conflicts between the parsimony tree for all molecular data and the ML/Bayesian tree for all molecular data (A). In C, the gray circle indicates the single conflict between the ML/Bayesian tree for nuclear data and the parsimony tree for nuclear data (not shown). The white circles in C mark nodes that are unresolved in the parsimony analysis. Higher-level cetacean taxa are delimited by brackets to the right of the tree in A.

The molecular partition includes mt genomes, transposon insertions, and segments of 69 nu loci that are distributed across the different chromosomes of the cow genome (Figure 3; Additional file 2: Table S1). Bayesian and ML analyses of the combined molecular data yielded identical trees (Figure 4A), and a Bayesian search in which the molecular dataset was partitioned by gene gave the same basic topology with similar support scores (not shown). The ML/Bayesian topology was congruent with the Bayesian consensus in a recent molecular supermatrix analysis [20] except that the positions of two delphinid species, *Orcinus orca *and *Leucopleurus acutus*, are swapped. In our trees, *L. acutus *is the sister species to all other delphinids included in our analysis, and *O. orca *is the sister species to the next most inclusive delphinid clade. Among the river dolphins, *Lipotes *groups with Inioidea (*Inia *+ *Pontoporia*), and *Platanista *is placed as the sister taxon to Delphinida plus Ziphiidae (Figure 4A). Relationships among odontocete families are congruent with the hypothesis of Nikaido et al. [15] (Figure 1O, 2P), and several subsequent studies (Figure 2V, W, X)

**Figure 5:**
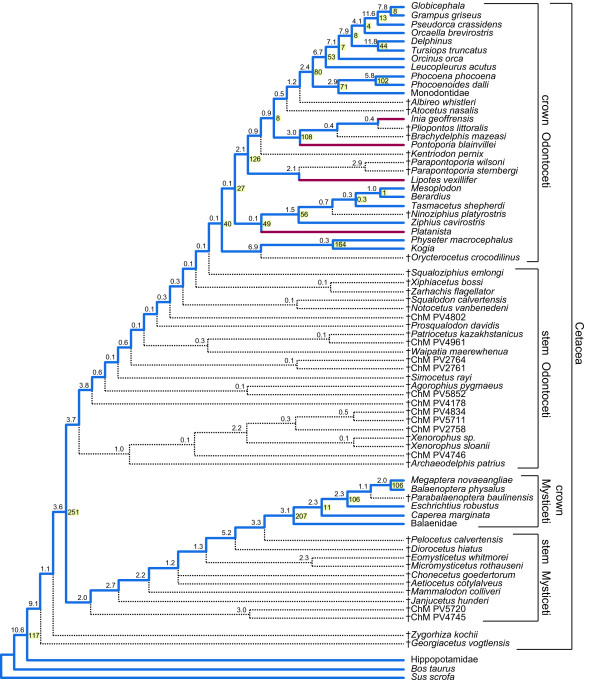
**Strict consensus of minimum length trees derived from parsimony analysis of the supermatrix**. Lineages that connect extant taxa are colored; river dolphin lineages are red, and other branches are blue. Dotted lines represent fossil lineages and lead to extinct taxa/OTUs (†). Branch support (BS) is above internodes, and double decay branch support (ddBS) is to the right of nodes that define relationships among extant taxa (cream background). BS and ddBS are expressed in terms of the number of extra steps beyond minimum tree length. BS scores are rounded up to the nearest tenth of a step, and ddBS scores are rounded up to the nearest step. Higher-level groupings are delimited by brackets to the right of the tree.

Our Bayesian/ML tree is largely congruent with the parsimony trees obtained from the analysis of our morphological and molecular supermatrix (described below) (Figure 5); 21 of 26 nodes that define relationships among extant taxa were the same (Figure 4A). Differences were largely due to contrasting methodologies and not to the inclusion of morphological/fossil data in the supermatrix; the tree supported by parsimony analysis of the molecular data alone (Figure 4B) is highly congruent with the parsimony analysis of the fossil + molecular supermatrix (Figure 5) and conflicts at the same five nodes with the trees from the Bayesian/ML analyses of the molecular data. In contrast to the explicitly model-based approaches, parsimony analysis of the molecular matrix positions *Lipotes *as the sister-group to the remaining delphinidans, and also resolves a clade composed of *Platanista *and Ziphiidae. Relationships within Ziphiidae do not match those supported by ML and Bayesian analyses (Figure 4A-B).

Nearly all nodes in the Bayesian/ML molecular tree were well supported; 21 nodes received a PP of 1.0 and bootstrap support of 100% (Figure 4A). Only one of these highly supported nodes (*Mesoplodon *+ *Ziphius *+ *Tasmacetu*s) did not occur in the parsimony analysis of the complete supermatrix (Figure 5). Several other clades that did not occur in the parsimony trees received less support: *Mesoplodon *+ *Ziphius *(PP = 0.91, ML = 80%), *Lipotes *+ Inioidea (PP = 0.98, ML = 51%), *Globicephala *+ *Pseudorca *(PP = 0.97, ML = 80%), and Ziphiidae + Delphinida (PP = 0.98, ML = 63%). Most clades favored by parsimony analysis of the molecular data that conflicted with Bayesian and ML results were weakly supported (bootstrap scores <50 to 55%; Figure 4B). The lone exception was the grouping of *Platanista *with Ziphiidae (bootstrap = 95%). When the nu DNA data were analyzed in isolation from the very large and more rapidly evolving mt DNA partition using parsimony or model-based methods (Figure 4C), results closely matched the combined molecular tree derived from ML/Bayesian analyses (Figure 4A).

Our overall supermatrix merged information from the fossil record and from the mt and nu genomes. Two most parsimonious trees were found for the combined supermatrix, each 41081.07 steps in length, and the strict consensus of these two trees is well resolved (Figure 5). The minimum length trees vary only in the positions of the extinct toothed mysticetes *Aetiocetus cotylalveus *and *Chonecetus goedertorum*; in one they form a monophyletic Aetiocetidae, which has been supported by several studies [2,41-45], whereas in the other, *Aetiocetus *is more closely related to Chaeomysticeti (edentulous mysticetes) than is *Chonecetus*, a result previously obtained by Fitzgerald [46]. Relationships among extant taxa are the same in both trees, with the strict consensus reconstructing many traditionally recognized taxa as monophyletic (Figure 5). Because of the instability of many extinct taxa in our trees, we calculated double decay branch support (ddBS [47]) in addition to branch support (BS) scores [48]. We used ddBS to measure the stability of relationships among extant taxa within the context of evidence from the complete matrix, including fossils. Branch support was used to measure the character support for relationships among all taxa, extant and extinct, in our trees (see Materials and Methods). Clades that received high ddBS values include: Mysticeti (BS = 2.0, ddBS = 206.98), Balaenopteridae (BS = 2.0, ddBS = 106.24), Balaenopteroidea (BS = 2.34, ddBS = 105.55), Odontoceti (BS = 3.69, ddBS = 40.13), Physeteroidea (BS = 6.85, ddBS = 163.82), Ziphiidae (BS = 1.53, ddBS = 55.76), Delphinida (BS = 2.07, ddBS = 125.90), Delphinoidea (BS = 0.53, ddBS = 79.89), Inioidea (BS = 3.04, ddBS = 107.53), Phocoenidae (BS = 5.81, ddBS = 101.95), Delphinidae (BS = 6.65, ddBS = 53.05), Delphininae (BS = 11.84, ddBS = 44.25), and Globicephalinae (BS = 11.64, ddBS = 12.61).

Branch support values generally are low (Figure 5). This is primarily attributed to the inclusion of extinct taxa that can only be scored for the morphology partition and which subdivide long internodes into much shorter internal branches. Corresponding ddBS values are dramatically higher than BS, often by more than a factor of 10. Of the 70 nodes in the strict consensus, 15 (21%) received very low BS values (i.e. 0.13 steps). Many of these weakly supported nodes connect to branches that are situated on the stem lineage to crown Odontoceti. Nodes within Delphinidae, where no fossils were sampled, have much higher BS values, some of which are identical to the ddBS values (i.e. *Grampus *+ *Globicephala *[7.82]; Delphinidae excluding *Leucopleurus *[7.12]). Where comparable, BS values are lower than those reported by Geisler and Sanders [2]. Some of this reduction is attributed to the addition of 17 taxa to their morphological matrix. For example, Geisler and Sanders [2] did not include the extinct ziphiid *Ninoziphius platyrostris*, which is known only from a partial skeleton with poorly preserved skull. The BS for Ziphiidae is much lower than that found by Geisler and Sanders [2] (1.53 vs. 10 steps); however, a double decay analysis that ignores the phylogenetic position of *Ninoziphius*, but not the positions of other extinct taxa, increases the BS to 7.07 steps.

Relationships among odontocete families are broadly congruent with published molecular and morphological studies (Figure 5). Physeteroidea is the extant sister group to all other living odontocetes, consistent with numerous molecular studies (Figure 1J-O, Figure 2L, N-P, R-Z) and some morphological analyses (Figure 1D, E, and 1I). The grouping of Physeteroidea with Ziphiidae, as found in some morphological analyses (Figure 1F-H; Additional file 1: Fig. S1), was not supported. As in the majority of recent molecular studies, Monodontidae is the sister-group to Phocoenidae [49] with Inioidea, Delphinida, and also Delphinoidea recovered (Figures 1, 2).

**Figure 6:**
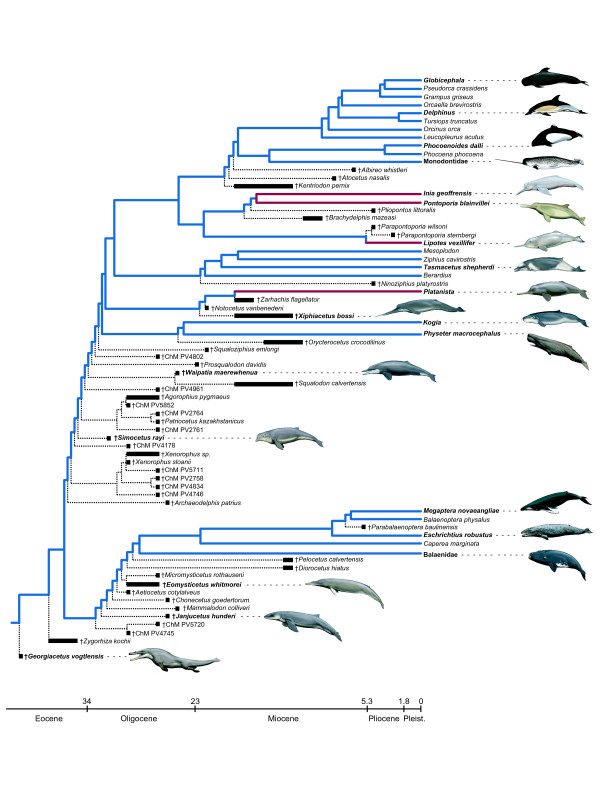
**Strict consensus of minimum length trees derived from parsimony analysis of the morphological data with implied weighting (k = 3) and relationships among extant taxa constrained to fit the ML/Bayesian analysis of all molecular data (Figure 4A)**. Lineages that connect extant taxa are colored; river dolphin lineages are red, and other branches are blue. Dotted lines represent fossil lineages and lead to extinct taxa/OTUs (†). Divergence times between extant taxa in the tree are according to the molecular clock analysis of McGowen et al. [20]; divergences of extinct taxa/OTUs are based on first and last appearances in the fossil record (thick black bars; see Table 2 and Additional file 2: Table S2). Note that all Oligocene or older cetaceans fell outside of crown Odontoceti and outside of crown Mysticeti. The molecular clock divergences among extant taxa [20] are shown here to contrast with the patterns recorded in the sampling of the fossil record in our study. Cetacean OTUs in bold followed by "- - - -" are associated with paintings to the right.

A more controversial finding is a sister-group relationship between *Platanista *and Ziphiidae (Figure 5). Although the BS for this node is low (0.13), the ddBS is near the median of recovered values at 48.79. This sister-group relationship had previously been recovered in multiple previous analyses (Figure 1C, L, M, 2S, Y, Z; Figure 4B-C). Alternatively, *Platanista *has been placed as the sister-group to Delphinida + Ziphiidae (Figure 1B, K, N, O, 2P, Q, V-X; Figure 4A and 4D). A third alternative places *Platanista *as the sister-group to Delphinida as in Heyning [4,5] (Figure 1D) among others (Figure 1A, F, I, J, 2R, T, U), but this grouping was not recovered in any of our phylogenetic analyses (Figures 4, 5, 6).

Similar to the situation with *Platanista*, the placement of *Lipotes vexillifer *has varied among previous studies; the combined parsimony analysis agrees with Messenger and McGuire [1] (Figure 1A) and others (Figure 1F, K-N, Figure 2Q) in placing the Yangtze River dolphin as the extant sister-group to a clade composed of Delphinoidea and Inioidea. This conflicts with multiple studies that position *Lipotes *as the extant sister-group to Inioidea (Figure 1B, D, J, 2P, R-Z). It should be noted that the support for *Lipotes *as the extant sister-group to Delphinoidea + Inioidea, instead of as the extant sister taxon of Inioidea, is marginal (BS = 0.87; ddBS = 7.48). The closest relative to *Lipotes *is the extinct genus *Parapontoporia*, here represented by two species, *P. wilsoni *and *P. sternbergi*. This placement is consistent with some morphological studies [2,8] but is at odds with others that consider *Parapontoporia *to be a pontoporiid [50,51].

Relationships within Ziphiidae are somewhat unconventional (Figure 5). As in the morphological analysis of Geisler and Sanders [2], *Mesoplodon *is more closely related to *Berardius *than to *Ziphius *or *Tasmacetus*. Such an apical position for *Berardius *contradicts previous morphological [52-54] and molecular hypotheses [20,55]. An important caveat to the ziphiid relationships found here is the very low series of BS and ddBS values for these nodes (all ≤ 1.05 steps).

Within Delphinidae, *Orcaella brevirostris *is placed as the sister-group to Globicephalinae, a result that is consistent with the molecular analysis of Caballero et al. [37] and the supermatrix tree of McGowen et al. [20], but contradicts the smaller supermatrix of Steeman et al. [21], the supertree of Price et al. [25], and analysis of mt DNA data [19,24]. Within Globicephalinae, *Globicephala *is more closely related to *Grampus *than to *Pseudorca*, a result that was weakly supported by LeDuc et al. [36]. Among the delphinids included in our analysis, *Leucopleurus acutus *is positioned as the sister-species to all remaining delphinids, with *Orcinus orca *branching from a more apical node as the sister to all delphinids except *L. acutus*. Although McGowen et al. [20] also recovered a tree in which these two taxa represented early branching events within Delphinidae, the positions of these taxa were reversed with *Orcinus*, not *Leucopleurus*, as the sister-group to all other extant delphinid species.

Of the 45 extinct taxa and/or specimens included in the analysis, the majority were determined to be stem odontocetes (Figure 5). A similar result was recovered by Geisler and Sanders [2] based on an earlier dataset that was further augmented and modified in the current study. Overall, the arrangement of stem odontocetes is quite similar, with Xenorophidae as the first odontocete branch, followed *Agorophius*, *Patriocetus*, and undescribed taxa intercalated among these three named taxa. However, some topological details differ from the tree supported by Geisler and Sanders [2]. For example, in the strict consensus of the current study, *Waipatia *forms a clade with *Patriocetus *and an undescribed form from South Carolina (ChM PV4961). Somewhat surprisingly, the putative platanistid *Zarhachis *and the eurhinodelphid *Xiphiacetus *did not fall inside crown Cetacea but instead form a clade outside of it (but see below). Eurhinodelphids have typically been placed within crown Odontoceti; however, there has been no consensus beyond that. Eurhinodelphids have been considered as a stem group to Ziphiidae [11], sister-group to Delphinida [9,10], or sister-group to Squalodontidae + Squalodelphidae [6]. *Simocetus rayi*, which was not sampled by Geisler and Sanders [2], here is placed slightly more apical in the cladogram than *Agorophius *and an unnamed taxon (ChM PV5852). As noted above, relationships among stem odontocetes in the parsimony analysis of the supermatrix were weakly supported (Figure 5).

Within the odontocete crown group, two extinct taxa, *Pliopontos *and *Brachydelphis*, are positioned inside Inioidea (Figure 5). The placement of *Brachydelphis *inside Inioidea contrasts with an analysis of an earlier version of the morphological dataset utilized here [2]; the authors of that study reconstructed *Brachydelphis *as a stem platanistoid (sensu [56]). Identifying *Pliopontos *and *Brachydelphis *as inioids is in agreement with a previous phylogenetic hypothesis [9], although unlike that study and others [51,57-59] these two taxa are here reconstructed as successive stem taxa to *Inia*, instead of being positioned inside Pontoporiidae. *Kentriodon pernix *and *Atocetus nasalis*, two extinct species from the possibly paraphyletic Kentriodontidae, were also included in the analysis. The former is the sister-group to Delphinoidea + Inioidea whereas the latter is the sister-group to Delphinoidea in our minimum length trees (Figure 5).

The Miocene mysticetes *Diorocetus *and *Pelocetus *are immediately outside the mysticete crown group (Figure 5). Historically such taxa were referred to as cetotheres [60,61], but more recent systematic work on mysticete phylogeny has redefined Cetotheriidae to a monophyletic family that excludes these taxa [62]. Regardless, the phylogenetic positions of these Miocene mysticetes are controversial, with some studies excluding them from the crown group [2,44-46,62,63], which is supported here. Other studies place some Miocene mysticetes as more closely related to balaenopterids [38] or to balaenopterids and eschrichtiids [39,41,42,64]. Within crown Mysticeti, Balaenidae is sister to the remaining extant lineages and *Caperea *is sister to a monophyletic Balaenopteroidea, as has been found in many analyses of DNA sequence data [30,65], molecular supermatrices [20,21], a recent morphological analysis [66], and in an analysis that combined morphological and molecular data [43]. Balaenoidea (Neobalaenidae + Balaenidae), which is favored by nearly all morphological studies (Additional file 1: Fig. S1) [2,38,39,42,43,62], is not supported. *Parabalaenoptera *is a stem balaenopterid as in previous work ([39,44] but see [63]).

In addition to the parsimony analysis of the supermatrix, we also executed a morphological analysis with ML/Bayesian molecular constraints. This search yielded three trees, each with a score of 15214.10, which differ only in the relationships of three xenorophids: *Xenorophus sloanii*, an undescribed species of *Xenorophus *(*Xenorophus *sp.), and another undescribed taxon (ChM PV5711) (Figure 6). Given the backbone constraint, relationships among extant taxa are identical to those obtained by ML and Bayesian analyses of the molecular partition (i.e. 26 nodes); however, 11 of these nodes are also supported by the parsimony analysis of morphology (Additional file 1: Fig. S1) and three more are supported when implied weighting was applied to morphology (Additional file 1: Fig. S2). Implementation of the ML/Bayesian constraint and implied weighting changed the positions of several extinct OTU's (operational taxonomic units) as compared to the combined parsimony analysis of morphology and molecules (Figure 5). Unlike our parsimony analysis of the supermatrix and the hypothesis of Geisler and Sanders [2], *Archaeodelphis *is placed as the sister-group to all remaining odontocetes instead of being closely related to *Xenorophus *(Figure 6). The trees obtained with the ML/Bayesian constraint separate *Archaeodelphis *from the Xenorophidae, as defined by Uhen [67]. Unlike the combined parsimony tree, the clade including *Agorophius *and an undescribed OTU (ChM PV5852) is positioned in a group of Oligocene taxa that includes *Patriocetus kazakhstanicus*. This contradicts the allocation of *Patriocetus *to the Squalodontidae [68,69].

The ML/Bayesian molecular constraint positions *Platanista *as the extant sister-group to Ziphiidae plus Delphinida. Enforcing this relationship resulted in the recovery of a platanistoid clade, although here Platanistoidea includes Squalodelphinidae (represented by *Notocetus vanbenedeni*) but not Waipatiidae and Squalodontidae (contra [10,70]). *Xiphiacetus *(Eurhinodelphidae) is here placed as the sister-group to platanistoids, unlike the unconstrained analysis where it was positioned outside of the odontocete crown group. Another difference is that Platanistidae (*Platanista *+ *Zarhachis*) is monophyletic in the analysis with the ML/Bayesian constraint. Moving to more apical nodes in the tree, the only extinct ziphiid included in the analysis (*Ninoziphius*) is sister to a clade composed of the remaining ziphiids, whereas in the unconstrained parsimony analysis *Ziphius *is sister to all other ziphiids in our sample (Figure 5). Within Delphinida, the extinct taxon *Kentriodon *moved from being outside Inioidea + Delphinoidea to being an early-branching stem delphinoid, and phylogenetic relationships within Inioidea were rearranged (Figure 6).

### Temporal Implications of Phylogenetic Hypotheses

The fit between the fossil record (Table 2; Additional file 2: Table S2) and all minimum length trees recovered from the four parsimony analyses that included extinct taxa (Figures 5, 6; Additional file 1: Figs. S1, S2) was measured by the modified Manhattan stratigraphic measure (MSM*) and the gap excess ratio (GER) (Additional file 2: Table S3). All trees implied substantial ghost lineages as indicated by the fairly low MSM* scores (0.11-0.12); however, GER scores, which are standardized by the maximum possible sum of all ghost lineages, are much higher (0.83-0.84). The MSM* scores for all trees are statistically significant (p = 0.001). Taken together, these results suggest that the fossil record of Neoceti (as sampled in the present study) is reasonably good.

**Table 2:** Ages, cladistic codings for the stratigraphic character, and distributions of extinct taxa included in the phylogenetic analyses.

Taxon		FAD	LAD	Code	Distribution
	†*Georgiacetus vogtlensis*	41	-	0	USA: GA
	
	†*Zygorhiza kochii*	38	35	1	USA: AR, MS, LA. AL, GA

Mysticeti					

	†*Aetiocetus cotylalveus*	30	-	3	USA:OR
	
	†ChM PV4745	30	-	3	USA: SC
	
	†*Micromysticetus rothauseni*	30	27	3	USA: SC
	
	†ChM PV5720	27	-	4	USA: SC
	
	†*Eomysticetus whitmorei*	27	-	4	USA: SC
	
	†*Chonecetus goedertorum*	26	25.8	5	USA: WA
	
	†*Janjucetus hunderi*	26	-	5	Australia
	
	†*Mammalodon colliveri*	25	24.8	6	Australia
	
	†*Diorocetus hiatus*	14	13	B	USA: MD, VA
	
	†*Pelocetus calvertensis*	14	13	B	USA: MD, VA
	
	†*Parabalaenoptera baulinensis*	6	-	F	USA: CA

Odontoceti					

	†*Simocetus rayi*	32	-	2	USA: OR
	
	†*Agorophius pygmaeus*	30	27	3	USA: SC
	
	†ChM PV4178	30	-	3	USA: SC
	
	†ChM PV5852	30	-	3	USA: SC
	
	†*Xenorophus sloanii*	30	-	3	USA: SC
	
	†*Xenorophus *sp.	30	27	3	USA: SC
	
	†ChM PV2758	27	-	4	USA: SC
	
	†ChM PV2761	27	-	4	USA: SC
	
	†ChM PV2764	27	-	4	USA: SC
	
	†ChM PV4746	27	-	4	USA: SC
	
	†ChM PV4802	27	-	4	USA: SC
	
	†ChM PV4834	27	-	4	USA: SC
	
	†ChM PV4961	27	-	4	USA: SC
	
	†ChM PV5711	27	-	4	USA: SC
	
	†*Patriocetus kazakhstanicus*	27	-	4	Kazakhstan
	
	†*Archaeodelphis patrius*	26	-	5	USA: SC*
	
	†*Waipatia maerewhenua*	25	-	6	New Zealand
	
	†*Prosqualodon davidis*	23	-	7	Tasmania, Australia
	
	†*Squaloziphius emlongi*	22	-	8	USA: WA
	
	†*Notocetus vanbenedeni*	22	21.8	8	Argentina
	
	†*Squalodon calvertensis*	19	13	9	USA: DE, MD, NC, VA
	
	†*Kentriodon pernix*	19	13	9	USA: MD, VA

	†*Xiphiacetus bossi*	19	13	9	USA: MD, VA; Belgium
	
	†*Zarhachis flagellator*	19	17	9	USA:MD, DE
	
	†*Orycterocetus crocodilinus*	16	12	A	USA: MD, VA; Belgium; France
	
	†*Brachydelphis mazeasi*	12	10	C	Peru; Chile
	
	†*Atocetus nasalis*	9	8.8	D	USA: CA
	
	†*Albireo whistleri*	7	6.8	E	Mexico
	
	†*Ninoziphius platyrostris*	5	4.8	G	Peru
	
	†*Parapontoporia wilsoni*	5	-	G	USA: CA
	
	†*Pliopontos littoralis*	5	4.8	G	Peru
	
	†*Parapontoporia sternbergi*	3	2.8	H	USA: CA

In cases where the FAD and LAD cannot be distinguished, and multiple specimens are known, the LAD is arbitrarily placed 200 Ka after the FAD. FAD: first appearance datum; LAD: last appearance datum; * provenance uncertain. See Additional file 2: Table S2 and Additional file 3 for details and references.

Sister-group relationships and the first appearances of extinct taxa in the fossil record suggest that the diversifications of crown Odontoceti and crown Mysticeti postdated the Oligocene, in contrast to molecular clock studies that suggested earlier dates (Figure 6). The shortest suboptimal tree that includes an Oligocene taxon inside either crown Odontoceti or crown Mysticeti is 5.27 steps longer than the minimum length trees (Additional file 2: Table S4). In this suboptimal tree, the undescribed OTU represented by ChM PV4802 is positioned as a stem taxon to the clade that includes Delphinida, Ziphiidae, and Platanistidae. Although not supported by the supermatrix of the present study, this alternative topology could not be statistically rejected (Templeton test p value = 0.505-0.506; winning-sites p value = 0.218-0.257). The shortest suboptimal trees that include other Oligocene odontocetes inside crown Odontoceti are much longer (10.25-16.29 steps); however, these suboptimal topologies could not be rejected at a p value of 0.05 either. The shortest tree that includes *Agorophius *in the odontocete crown group (10.25 steps longer) is also the shortest tree that places *Simocetus*, *Patriocetus*, *Waipatia*, and *Prosqualodon davidis *in the odontocete crown group. This suboptimal tree positions those Oligocene odontocetes in a clade that is the sister-group to Delphinida + Ziphiidae + Platanistidae. Although this topology is still above the p = 0.05 threshold, its rejection approached statistical significance for the winning-sites test (p = 0.051). The shortest tree that places *Waipatia *inside Platanistoidea, as advocated by some studies [10,70], is 13.83 steps longer than the minimum length trees, although this too could not be rejected at the p = 0.05 level (Templeton test p value = 0.152-0.168; winning-sites p value = 0.096-0.118).

The shortest suboptimal trees that included Oligocene mysticetes within crown Mysticeti were somewhat longer than their counterparts on the odontocete side of the tree (13.72-20.84 steps longer than minimum length trees). Most of these hypotheses could be rejected at p ≤ 0.05 for the Templeton and winning-sites tests; the sole exception was a topology that placed *Eomysticetus whitmorei *as the sister-group to Balaenidae (Templeton test p value = 0.115-.121). Even though the combined supermatrix dwarfs the morphological partition in size, the same p values are obtained when Templeton and winning-sites tests are conducted on the morphological partition because the suboptimal trees differ from the minimum length trees in the positions of extinct, not extant, taxa.

### The Evolution of River Dolphins

**Figure 7:**
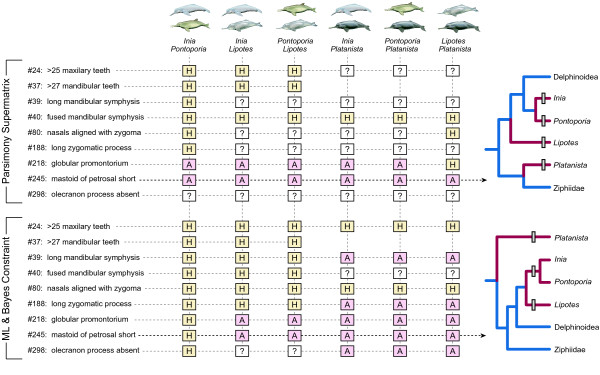
**Homology ("H" in tan boxes) versus analogy ("A" in pink boxes) of character states shared by extant river dolphins including *Pontoporia***. Results for parsimony optimizations of nine characters on two trees, parsimony supermatrix (Fig. 5) and ML/Bayesian constraint of morphology (Fig. 6), are shown for all pairwise comparisons between genera of river dolphins. Question marks indicate cases where optimizations and resulting estimates of homology versus analogy were ambiguous. Trees on the right show alternative mappings of character 245 on the two phylogenetic hypotheses: four transitions to the shared river dolphin state (mastoid of petrosal short) for the parsimony tree and three transitions for the ML/Bayesian constrained tree. Note that many extant and extinct taxa have been pruned from the illustrated trees, but all taxa were considered in the actual character optimizations (Figs. 5, 6). Branches are colored as in Fig. 1.

Not surprisingly, all morphological character states shared by river dolphins cannot be simply described as convergences, reversals, or symplesiomorphies. We focused on nine of the potential river dolphin "synapomorphies" listed by Geisler and Sanders [2], specifically those that occur in at least three of the four extant genera. River dolphin characters were optimized onto the trees derived from parsimony analysis of the supermatrix (Figure 5) and those supported by analysis of the morphological data with ML/Bayesian molecular constraints (Figure 6). Pairwise comparisons among the four river dolphin genera for each of the nine characters summarize whether shared similarities in character states between genera are most simply interpreted as homologous - inherited from a common ancestor, or analogous - independently derived through either convergence or reversal (Figure 7). When the character state similarities among all extant river dolphins are homologous, this implies symplesiomorphy (e.g. fused mandibular symphysis on parsimony trees; number of maxillary teeth and position of nasals on ML/Bayesian constraint trees). By contrast, when all pairwise similarities among genera are analogous, the character is purely convergent and evolved independently in each of the four river dolphin genera (e.g. length of mastoid process on parsimony trees). If some but not all states are homologous, the simplest explanation is either that the character experienced reversal(s) (e.g. globular promontorium on parsimony trees) or, more commonly, convergent evolution to similar character states in two or three separate lineages. Several characters previously interpreted as synapomorphies of all extant river dolphins [2] are here interpreted as synapomorphies of more exclusive clades, such as Inioidea (globular promontorium and short mastoid process on constraint trees) or Iniodea plus *Lipotes *(long zygoma and mandibular symphysis on constraint trees). Alternatively, some characters previously interpreted as synapomorphies of all river dolphins are, in the context of our combined phylogenetic hypotheses, better interpreted as synapomorphies for even more inclusive clades (e.g. fused mandibular symphysis on parsimony trees). In many cases, character optimizations offer multiple equally parsimonious interpretations of the evidence, and at least some interpretations of homology versus analogy are ambiguous (question marks in Figure 7).

For the trees derived from parsimony analysis of the supermatrix, two characters are symplesiomorphic (number of mandibular teeth and fused mandibular symphysis). Character 218 (globular promontorium) is symplesiomorphic for all river dolphins, but there are two subsequent reversals to the primitive condition in *Inia *and in *Pontoporia *(Figure 7). Character 245 (short mastoid process) is purely convergent, with the morphology of each extant genus interpreted as the result of an independent derivation. The remaining five characters have ambiguous optimizations, but for character 80 (position of nasals), the inference of homology between two distantly related genera, *Lipotes *and *Platanista*, implies that this similarity was present in the last common ancestor of all extant river dolphin genera (Figure 7). In contrast, when these same nine characters are mapped on trees derived from parsimony analysis of morphology with the ML/Bayesian molecular constraint, a more consistent pattern of convergence is implied (Figure 7). Three shared character states are symplesiomorphic (numbers of maxillary and mandibular teeth, position of nasals), and four characters experienced one or more instances of convergence (long symphysis and zygoma, globular promontorium, and short mastoid process). The optimizations for fusion of the mandibular symphysis and absence of the olecranon process are uncertain, but the latter is interpreted as convergently evolved between *Platanista *and the remaining three genera (Figure 7). The discrepancies in character optimizations between the trees obtained from constrained and unconstrained parsimony analyses underscore the importance of resolving the phylogenetic positions of *Platanista *and *Lipotes *for understanding the evolution of morphological characters shared by these two taxa.

## Discussion

### Combination of Diverse Evidence and the Phylogeny of Neoceti

Our analyses found strong support for several traditionally recognized clades. Most notable is the support for Mysticeti, Inioidea, and Delphinida; the ddBS for each is more than 100 steps. All of our phylogenetic analyses that included molecular evidence (Figures 4, 5, 6) also supported three newly named clades; 1) Plicogulae (Balaenopteridae, Eschrichtiidae, plus *Caperea*), 2) Synrhina (Delphinida, Platanistidae, plus Ziphiidae), and 3) Monodontoidae (Monodontidae plus Phocoenidae). Based on the support we found in combined analyses of molecules and fossils and the fact that these clades have been recovered by numerous previous studies (e.g., [49,66]), we provide names, definitions, and morphological diagnoses for each (Appendix 1).

A parsimony search of the complete supermatrix (Figure 5) and analysis of the morphological data with ML/Bayesian molecular constraints (Figure 6) showed conflicting phylogenetic positions for two river dolphins, *Platanista *and *Lipotes*, despite the fact that we added six new nu gene fragments for the latter taxon. Although we do not have a strong preference for either hypothesis, we do note that only the analysis with the ML/Bayesian constraint allocates the extinct taxa *Zarhachis *and *Notocetus *to the Platanistoidea, a result supported by some morphological analyses [10,11,70]. Furthermore, two SINE transposon insertions, considered by some to be very reliable phylogenetic characters [71], support the constraint tree (Figure 6) and conflict with the parsimony analysis of the supermatrix (Figure 5). Clear resolution of remaining conflicts is critical, because the placements of fossils relative to different extant species, the timing of diversification, and the reconstruction of evolutionary changes are profoundly altered depending on the basic set of relationships among the major lineages of extant cetaceans (see Results above and Discussion below). However, given the conflicts among separate analyses of smaller datasets regarding the placements of *Platanista *and *Lipotes *(Figures 1, 2), the amount of data included in our supermatrix (Figure 3), and the generally weak support for the placement of *Lipotes *in all of our concatenated analyses (Figures 4, 5), it is clear that the interrelationships of these river dolphins represent challenging systematic problems that may require a more complete matrix with less missing data, a genome-scale dataset, or a much broader sampling of extinct taxa to derive a consistently robust resolution.

### Dating the Radiations of Crown Odontoceti and Crown Mysticeti

We used the first appearances between sister taxa in the fossil record to infer minimum dates of divergence at particular nodes in our trees. In all analyses that included extinct taxa, we were not able to confirm the hypothesis based on molecular clocks that eight to ten distinct lineages of cetaceans that have extant representatives existed by the end of the Oligocene [13,20,21,24]. All 25 Oligocene OTU's sampled in the present study (~34% of the total taxonomic sample) were positioned outside of crown Mysticeti and crown Odontoceti, implying post-Oligocene ages for these two crown clades. Although the significance of this result in comparison to the molecular clock studies is hard to gauge, it suggests one or more possibilities: 1) the fossil record of Oligocene cetaceans is poor, 2) the fossil record of Oligocene cetaceans is good, but we inadvertently excluded Oligocene members of the crown groups from our phylogenetic analyses, 3) molecular clocks have overestimated the dates for the earliest splits in crown Odontoceti and Mysticeti, or 4) the morphological character data simply are not sufficient for robust resolution of these relationships. The first possibility appears unlikely given the significant correlation (i.e. p ≤ 0.001) between the topologies recovered here and the fossil record. The second possibility is more likely; our study heavily samples Oligocene, described and undescribed OTU's from the Southeastern United States. If Oligocene faunas were highly endemic, and the radiations of Odontoceti and Mysticeti did not occur in the Southeastern United States, then exclusion of Oligocene taxa from crown Odontoceti and from crown Mysticeti may not be a surprising result.

Examples of Oligocene taxa/specimens that have been referred to clades within crown Odontoceti, but were not sampled here because they could not be coded based on published descriptions, are *Oligodelphis*, a putative delphinoid, and *Ferecetotherium*, a putative physeteroid. Both taxa are only represented by fragmentary holotypes that were collected from the Maikop Series near the town of Perikeshkul, Azerbaijan. Recent chemostratigraphic and biostratigraphic work [72] indicates that the lower Miocene and upper Oligocene are equally represented (in terms of stratigraphic thickness) at the outcrops near Perikeshkul. Thus future work is needed to determine if these two taxa in fact came from the Oligocene part of the section and if they have been accurately allocated to Delphinoidea and Physeteroidea.

A review of recent literature would suggest that there are several accepted records of Platanistoidea in the Oligocene [3]. As mentioned in the results section, unlike Fordyce [10] we did not find *Waipatia *to be a member of Platanistoidea (also see [2,11]). Instead this taxon falls outside of crown group Odontoceti. Similarly we found *Squalodon *and *Prosqualodon *to be outside of the odontocete crown group and not within Platanistoidea, contrary to Muizon [73] and Fordyce [10]. Four of the Oligocene records of platanistoids on the paleobiology database are considered squalodelphinids, a family that fell inside Platanistoidea in our analysis with the ML/Bayesian constraint but not so in the unconstrained, combined parsimony analysis (Figures 5, 6). Three of these records are not well substantiated, but *Notocetus marplesi*, from the late Oligocene Otekaike Limestone Formation [74], was positioned as the sister-group to the clade of *Notocetus vanbenedeni *+ *Squalodelphis *in the phylogenetic analysis of Fordyce [10]. Although we have not had an opportunity to examine the holotype and only reported specimen of this taxon, allocation of this species to Squalodelphinidae would only extend the range of this family by 1 to 3 million years (*Notocetus vanbenedeni*, which is included in our analyses, is known from the earliest Miocene). As discussed in more detail below, the occurrence of at least a few lineages of crown odontocetes in the late Oligocene is to be expected.

Among described Oligocene mysticetes, Steeman [47] suggested that one taxon, *Mauicetus parki*, is a member of crown Mysticeti. The holotype consists of part of the postorbital region of the skull, and previously published descriptions suggest limited fossil remains that can only be identified as a chaeomysticete [75,76]. Steeman [42] reported that one of the petrosals of the holotype had been freed from the skull, and her analysis of morphological data placed this Oligocene form not only within the crown group but also within Balaenopteroidea. If correct, then molecular estimates for the radiation of Balaenopteroidea are substantially (i.e. 6-10 Ma) underestimated [20,21,30]. However, it is difficult to compare the topology of Steeman [21] to phylogenetic studies of molecular data that are sampled at the species level. Steeman [21] included the genera *Eschrichtius *and *Balaenoptera *as OTUs (the latter a composite based on *B. musculus *and *B. acutorostrata*) and found these genera to be separated by multiple extinct mysticetes, including *Mauicetus parki*. By contrast, some molecular analyses have found *Eschrichtius *to be nested within the genus *Balaenoptera *[20,24,65]. Furthermore, the positions of many extinct mysticetes in Steeman's tree differ sharply from the only studies on mysticete systematics that included molecular and fossil data [43,63]. Regardless, *Mauicetus parki *should be included in future analyses that combine morphological and molecular data, particularly now that the petrosal, which has many diagnostic features, is available for study.

Other putative records of crown odontocetes or crown mysticetes in the Oligocene consist of undescribed taxa that we have not had the opportunity to study. Ichishima et al. [77] and Steeman et al. [21] mentioned a specimen from the Oligocene of New Zealand which they referred to as "*Kentriodon *? sp." or as "cf. *Kentriodon*." However, in neither case was the morphology of this specimen described or the basis for this identification discussed. In a meeting abstract, Fordyce [78] introduced the first putative stem balaenid mysticete from the late Oligocene, based on a partial skull and other elements (OU 22224). The morphology of the specimen was briefly described, although Fordyce did not specify what features ally it with extant balaenids. Steeman [42] included an undescribed taxon from the Oligocene of New Zealand (ZMT 67) in her phylogenetic analysis, which was positioned as the sister-taxon to *Mauicetus parki*. Thus placed, this taxon would be a member of the mysticete crown group as well as a stem balaenopteroid; however, as with *Mauicetus parki*, it is difficult to reconcile the position of ZMT 67 in light of recent molecular phylogenies based on species-level OTUs that conflict with the basic structure of Steeman's [42] phylogenetic hypothesis.

The above discussion should not be understood as a rejection of any putative crown odontocete or mysticete in the Oligocene. To the contrary, we think that several early splits in crown Odontoceti and crown Mysticeti did occur in the late Oligocene because the oldest undisputed physeteroids [79,80] and a balaenid [81,82] are known from the earliest Miocene. However, what is known of the late Oligocene fossil record consists predominantly of plesiomorphic odontocetes and mysticetes, many with long intertemporal regions, some with external nares that are anterior to the orbits, and numerous mysticetes that retain teeth [83]. As noted above, possible exceptions to this pattern that require further investigation are *Ferecetotherium*, which is likely a physeteroid and possibly Oligocene in age, and *Notocetus marplesi*, which is Oligocene in age and is possibly a squalodelphinid. Certainly other undescribed taxa that have been tentatively placed in crown Odontoceti or crown Mysticeti should be described and placed in phylogenetic analyses as well [78,42,21], but we caution against the use of these undescribed taxa as calibration points in molecular clock studies until that is done [21,20,24,84]. Our phylogenetic analyses, which are based on a reasonable sample of the known Oligocene cetacean fossil record, do not support the radiation of crown Mysticeti and crown Odontoceti in the early Oligocene, as reconstructed by several molecular clock studies [20,21,24]. The timing of these basal splits is critical because if they occurred in the earliest Oligocene, then they would have coincided with an interval of pronounced ocean cooling [21] that may have helped spur early neocete evolution [85].

Given our criticism of some of the calibration points used in molecular clock studies, we present a more conservative, and better justified, set of points (Table 3). The first four calibrations are preferred because they are based on fossil taxa that have had their relationships to extant taxa determined by computer-assisted phylogenetic analyses of datasets that include molecular and morphological data. The phylogenetic positions of *Kentriodon pernix *and *Simocetus rayi*, which provide minimum ages for Delphinida and Neoceti respectively, are based on the current study, with ages supported by work listed in Table S2 (Additional file 2). A minimum age for Plicogulae is provided by "*Megaptera" miocaena *[43], which is at least 7.2 Ma in age [63], and we agree with van Tuinen and Hadley [86] in considering Pakicetidae as a good calibration point for the clade referred to as Whippomorpha or Cetancodonta [87,88], which is constrained to be older than 47 Ma [89].

**Table 3:** Calibration points for molecular clock analyses.

Preferred Calibration Points		
Node	Taxon	Age Range of FAD

Plicogulae	*"Megaptera" miocaena*	7.2-11.6 Ma

Delphinida	*Kentriodon pernix*	18.5-19.5 Ma

Neoceti	*Simocetus rayi*	30.5-32.3 Ma

Cetancodonta	Pakicetidae	47-52 Ma

		

**Other Suggested Calibration Points**		

Monodontoidae	*Salumiphocaena stocktoni*	7.5-9.5 Ma

Crown Ziphiidae	*Archaeoziphius microglenoideus*	13.2-15 Ma

Synrhina	*Notocetus vanbenedeni*	20-23 Ma

Crown Mysticeti	*Morenocetus parvus*	20-23 Ma

Neoceti	*Llanocetus denticrenatus*	34-35 Ma

Relationships of taxa in the "preferred calibration points" are based on phylogenetic analyses that combine molecular and morphological data. Taxa in "other suggested calibration points" either vary in their position among combined analyses of molecules and morphology or have been placed based on phylogenetic analysis of morphological data only. Nodes refer to node-based definitions of these taxa. FAD: first appearance datum. See text for supporting references.

We are less confident about the last five calibration points (Table 3). Regarding a minimum age for Synrhina, placement of *Notocetus *within Platanistoidea is supported by our analysis of the morphological partition with the Bayesian/ML constraint but not our parsimony analysis of the supermatrix. If this taxon is a platanistoid, then it constrains Synrhina to be at least 20 Ma [90]. The remaining four calibration points are deemed less reliable because they are based on phylogenetic analyses of morphological data only. As described in the results section, morphological and molecular data are at odds with respect to the phylogenetic positions of some extant taxa, thus it is unclear if the positions of extinct taxa would be stable to the addition of molecular data. *Morenocetus parvus*, the earliest described balaenid [82], is from deposits of the same age as *Notocetus *and, if accurately placed, implies that crown mysticetes emerged in the earliest Miocene. Also among the last five calibration points is *Llanocetus denticrenatus*, the earliest stem mysticete [42,45]. *Llanocetus *provides an older minimum age of 34 Ma [91,92] for Neoceti relative to our more conservative estimate based on the odontocete *Simocetus rayi*. Finally, the phocoenid *Salumiphocaena stocktoni *[93] and the ziphiid *Archaeoziphius microglenoideus *[54,94] potentially provide minimum ages of 7.5 Ma [95] and 13.2 Ma for Monodontoidae and the ziphiid crown group, respectively.

Our phylogenetic analyses support the exclusion of Oligocene cetaceans from crown Mysticeti and crown Odontoceti, however we used two statistical tests (Templeton/Wilcoxon rank sum and winning-sites tests) to determine whether our supermatrix strongly rejects hypotheses that include one or more Oligocene taxa in either of these clades. With one exception, we could reject all hypotheses that placed any of the Oligocene mysticetes we sampled in crown Mysticeti. The sole suboptimal topology that the Templeton test did not reject places Eomysticetidae as the sister-group to Balaenidae. Although a few characters do support a clade of eomysticetids and balaenids (absence of coracoid process of scapula, transverse groove on involucrum of bulla), the skull of eomysticetids is radically different from that of balaenids. Unlike the Templeton test, the balaenid + eomysticetid hypothesis was rejected by the winning-sites test (p = 0.017).

For odontocetes, positioning one or more of the Oligocene OTUs we sampled inside crown Odontoceti cost between 10.25 and 16.29 steps. One important exception was a tree that placed the undescribed OTU ChM PV4802 as the sister-group to Synrhina (5.27 steps longer). If this suboptimal tree is accurate, then at the end of the Oligocene there would have been at least three separate lineages of odontocetes that have extant descendants. ChM PV4802 is more like extant odontocetes than other Oligocene taxa we sampled in having extreme polydonty, probable homodonty (dentition is not completely preserved), near absence of the parietals from the skull roof, and development of a fossa on the palatine for the pterygoid sinus. It also shares with eurhinodelphids, ziphiids, and *Squaloziphius *a massive postglenoid process of the squamosal. Although inclusion of ChM PV4802 in crown Odontoceti is not supported by the present study, future analyses should include multiple Miocene ziphiids, eurhinodelphids, and platanistoids to determine whether these taxa "pull" ChM PV4802 into the crown group.

Most trees that position Oligocene taxa inside crown Odontoceti require many more steps than the minimum length trees, but the suboptimal topologies could not be rejected at p ≤ 0.05 using the Templeton or winning-sites tests. Thus, it is possible that the morphological data are simply not robust enough to determine, with confidence, whether certain Oligocene fossils are stem or crown odontocetes. However, detailed inspection of many of these trees reveals problematic patterns. The shortest tree that includes *Agorophius *in crown Odontoceti also places *Patriocetus*, *Simocetus*, *Prosqualodon davidis*, and *Waipatia *in the crown group (10.25 steps longer). This suboptimal tree requires the Oligocene odontocetes to exhibit step-wise reversals of cranial telescoping, the evolutionary process by which the external nares and rostral bones shifted posteriorly [96]. This tree also places the above Oligocene taxa inside the crown group in reverse stratigraphic order; taxa that branch from the most apical nodes are early Oligocene, and taxa that branch from the most basal nodes are late Oligocene. Similar inconsistencies between cranial telescoping and stratigraphy occur for the shortest trees that include *Waipatia *inside Platanistoidea (13.83 steps longer).

### Evolution of Riverine Odontocetes

**Figure 8:**
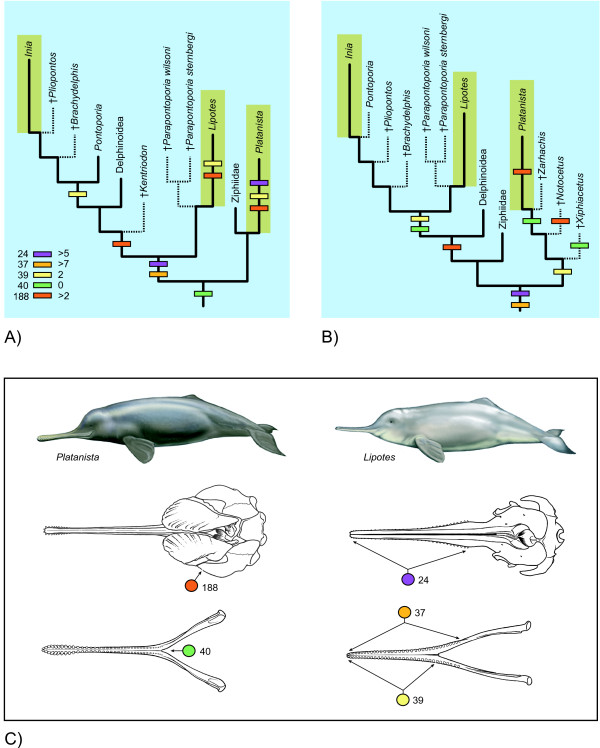
**The evolution of shared character states of river dolphins that are linked to prey capture**. Five characters (#s 24, 37, 39, 40, 188) are mapped onto the parsimony supermatrix tree (A; see Fig. 5) and the ML/Bayesian constrained tree (B; see Fig. 6) using delayed transformation optimization. Changes to the states shared by river dolphins are marked by colored bars on branches; character reversals in some taxa are not shown for simplicity. Marine (blue background) versus riverine (brown background) habitat also is optimized onto the two trees. In C, arrows point to characters 24, 37, 39, 40, and 188 in *Platanista *(left) and *Lipotes *(right). Dorsal views of skulls and mandibles are shown, and colors for characters are as in A and B.

The current consensus among morphologists is that *Platanista *(Indus and Ganges River dolphins) is not closely related to the two other extant river dolphins (*Inia *and *Lipotes*) or to the coastal dolphin *Pontoporia *[1,4,8,10]. The grouping that included all four genera was originally named Platanistoidea [56], although given that the group apparently is not monophyletic, Platanistoidea is now used instead to refer to *Platanista *and its extinct relatives [97]. One of the intriguing questions raised by non-monophyly of river dolphins is, how did they obtain such a disjunct distribution? By placing various marine odontocetes as the respective sister-groups to each extant river dolphin genus (Figures 5, 6, 7, 8), the current study supports suggestions that odontocetes invaded river systems on at least three occasions [13]. Hamilton et al. [14] expanded upon the hypothesis of separate, freshwater invasions by speculating that the ancestors of extant river dolphins remained in river systems after sea level regressed from its middle Miocene highs. In citing evidence for this hypothesis, they summarized published geologic evidence for a large epicontinental sea, called the Paranense Sea, in South America in the Miocene. They further suggested that the divergence of riverine *Inia *from marine *Pontoporia *was caused by the regression of the Paranense Sea. In criticizing this scenario, Steeman et al. [21] noted that their molecular clock estimate placed the divergence between these species at 20 Ma, well before the middle Miocene regressions mentioned by Hamilton et al. [14]. However, in calibrating their molecular clock, Steeman et al. [21] used the putative pontoporiid *Brachydelphis *to place a minimum age of 12 Ma on the *Inia/Pontoporia *divergence. In our Bayesian constraint trees, *Brachydelphis *is placed outside of the *Inia *and *Pontoporia *clade. Although the phylogeny of Inioidea varied among our analyses (Figures 5, 6), this does raise doubts about the appropriateness of *Brachydelphis *as a calibration point, and we consider the Hamilton et al. [14] scenario as worthy of further investigation in combined phylogenetic analyses of Cetacea.

Cassens et al. [13] also speculated that river dolphins persisted in river systems, but not in marine environments, either because 1) they did not have to compete with delphinoids for prey or 2) they were less affected by changes in ocean temperature or circulation. Although we are not able to test these hypotheses directly, current evidence casts doubt on both of them. Regarding competition, in the Yangtze River the range of *Lipotes *overlapped with the delphinoid *Neophocaena phocaenoides *and in the Amazonian River Basin, there is significant range overlap between *Inia *and the delphinoid *Sotalia fluviatilis *[98]. In the case of *Lipotes*, human activities, not competition with *Neophocaena*, have been implicated in its demise [99]. Similarly human activities are the primary threat to *Inia *as well [100]. Although positive evidence for the competitive exclusion hypothesis is wanting, river dolphins are sympatric with relatively few species of delphinoids in rivers. Thus it is possible that the intensity of competition, not the simple presence or absence of a single delphinoid species, could explain the absence of close relatives to *Lipotes *and *Platanista *in modern marine environments. Cassens et al. [13] cite the simultaneous decline of extinct, marine relatives of extant river dolphins with the increase of delphinoids as evidence to support their hypothesis. However, until now, none of the extinct taxa included in their diversity estimates had been analyzed explicitly in the context of molecular data, and most had not been included in computer-assisted phylogenetic analyses of morphology alone. As described above, we found one supposed platanistoid (*Waipatia*) to instead be outside of the odontocete crown group in two of our analyses (Figures 5, 6), along with several more putative platanistoids that branch from the stem lineage of Odontoceti in our parsimony analysis of the supermatrix (Figure 5). Thus we encourage future studies to include more putative, extinct relatives of river dolphins in total evidence analyses to test whether the simultaneous changes in delphinoid and river dolphin diversity are supported. Regarding the second hypothesis of Cassens et al. [13] that fluvial environments provided a refuge from changes in oceanic temperature and circulation changes, we note that a marine environment generally experiences less variability in temperature than a river due to a smaller surface area to volume ratio.

Now that there is a consensus that river dolphins do not form a natural group, it is useful to revisit the morphological evidence that initially led to the hypothesis that these taxa are very closely related. The most obvious morphological feature shared by *Pontoporia *and extant river dolphins is a narrow and elongate rostrum [6], although several authors have suggested that this feature is symplesiomorphic [2,32]. Even so, other characters that river dolphins share have a more restricted distribution, and in the most comprehensive morphological analysis to date, Geisler and Sanders [2] listed seven unambiguous synapomorphies for the subclade delimited by all extant river dolphins. The present study incorporates a modified version of the Geisler and Sanders [2] matrix, and analyses of the expanded matrix agree with other morphological studies in excluding *Platanista *from a close relationship with *Lipotes*, *Inia*, and *Pontoporia *(Additional file 1: Figs. S1, S2). However, we changed very few individual character codings from Geisler and Sanders [2], thus the morphological support for river dolphin monophyly, although now in the minority, still remains. With the trees of extant and extinct cetaceans obtained in the current study, we are able to scrutinize the morphological characters that support river dolphin monophyly and test hypotheses that were developed to explain their homoplastic behavior.

River dolphin paraphyly/polyphyly implies several possible evolutionary explanations for morphological characters shared by extant river dolphins and *Pontoporia*: 1) the characters are convergent, 2) the similarities are symplesiomorphic, 3) similarities are due to reversals, or 4) various combinations of these effects. Complicating the evaluation of these hypotheses is the positioning of extant river dolphins as successive sister-groups to Delphinoidea in some phylogenetic hypotheses based solely on living species (Figures 1, 4); thus according to parsimony optimization, it is unclear whether these similarities are convergent or are homologs with subsequent reversals in Delphinoidea and/or Ziphiidae. Futhermore, the positions of *Platanista *and several extinct taxa, which vary between the parsimony analysis of the supermatrix and the trees constrained by the ML/Bayesian topology, have a major impact on whether morphological characters shared by river dolphins are reconstructed as convergent, reversed, or symplesiomorphic (see RESULTS).

The fact that odontocetes restricted to rivers share several morphological features has led some to hypothesize that these features are adaptations to fluvial environments [13,101]. Key among these possible adaptations are features related to prey capture (Figure 8): >25 maxillary teeth (character 24, state 6 or 7), >27 mandibular teeth (37, state 8 or 9), long mandibular symphysis (39, state 2), fused mandibular symphysis (40, state 0), and long zygomatic process of squamosal (188, > state 2). Although it is possible that these characters are functionally related, each has passed initial tests of logical independence (sensu [102]; see [2]) and were therefore optimized on our trees as separate characters. If these characters states are in fact adaptations to a riverine environment, we would expect them to have evolved convergently in independent lineages of river dolphins. One of these character states, a large number of mandibular teeth (37), does not appear to be an adaptation of this type (Figure 8) because it is a symplesiomorphy of river dolphins on both sets of trees (note: *Platanista *does not share the "river dolphin" state). However, convergence remains a possibility for the other four characters. For example, according to the ML/Bayesian constrained trees, the elongate zygomatic process (188) and the long mandibular symphysis (39) of *Platanista *are convergent with those traits in Inioidea plus *Lipotes*; however, symplesiomorphy is an equally efficient interpretation on the parsimony tree for these two characters. Conversely, although a fused mandibular symphysis (40) is a symplesiomorphy on the parsimony tree, convergence between *Platanista *and Inioidea plus *Lipotes *is equally parsimonious to symplesiomorphy on the ML/Bayesian constrained trees (Figures 7, 8).

Given the above discussion, the elongate zygomatic processes, long mandibular symphyses, and other traits shared by river dolphins may be convergent, particulary if we accept the ML/Bayesian constrained trees (see Figure 7), but could these characters be adaptations to life in a fluvial environment? Extant odontocetes use two major modes of prey capture: 1) raptorial feeding where prey are seized by the teeth, and 2) suction feeding where prey are sucked into the mouth and teeth play little to no role in prey acquisition [103,104]. The suite of prey capture features shared by extant river dolphins are all correlated with raptorial feeding; however, there is no reason to think raptorial feeding is more efficient in rivers than in the ocean. Even more revealing are the paleoenvironments where fossils of extinct "river dolphins" have been found. On the ML/Bayesian constrained trees (Figure 6), *Platanista *is closely related to the extinct taxa *Zarhachis*, *Notocetus*, and *Xiphiacetus*, all of which have been found in sediments that were clearly deposited in marine environments [105]. All three of those extinct marine odontocetes have a long mandibular symphysis, two have a fused symphysis, and one has an elongate zygomatic process of the squamosal. Similarly, *Pontoporia*, *Parapontoporia*, *Pliopontos*, and *Brachydelphis*, which are close relatives of *Lipotes *and *Inia*, also occur in marine environments [106] or were found in marine sediments [105,107]. Two of these marine odontocetes have a long and fused mandibular symphysis, and three have a long zygomatic process. Clearly there is not a simple one-to-one correlation between the presence of river dolphin characters involved in prey capture and a riverine habitat.

**Figure 9:**
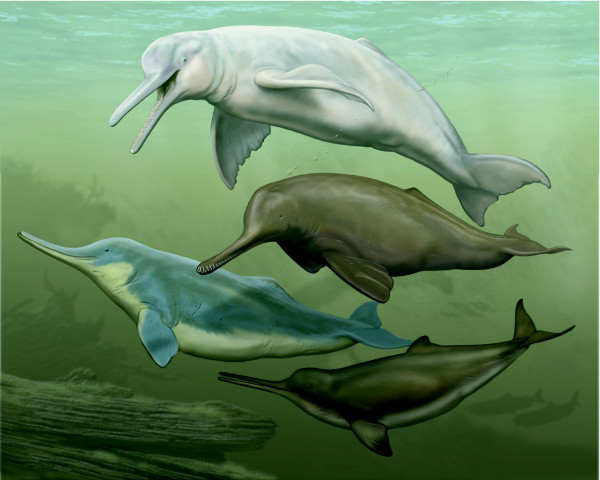
**External similarities among the three extant river dolphins and *Pontoporia***. The painting shows shared characteristics including long and narrow rostrum, small eyes, and broad forelimb flippers. A poorly-developed dorsal fin characterizes *Inia *(top), *Platanista *(second from top), and *Lipotes *(second from bottom) but is absent in the coastal *Pontoporia *(bottom). Note that the painting is for comparative purposes only; the geographic ranges of these species are disjunct.

If we optimize habitat on the parsimony trees (Figure 5) or the ML/Bayesian constrained trees (Figure 6), the simplest interpretation is that odontocetes switched from marine to riverine habitats three times on the terminal branches leading to *Platanista*, *Lipotes*, and *Inia *(Figure 8). To see if there is any support for the hypothesis that river dolphin characters related to feeding are adaptations to river environments, we optimized these characters using delayed transformation (DELTRAN) optimization (Figure 8). DELTRAN optimizations were employed because this procedure shifts as many character changes as possible to apical branches, where invasions of freshwater habitats occurred. If no pattern is found with DELTRAN optimizations, then these characters should not be interpreted as adaptations to riverine environments. The character mappings suggest that half (Figure 8A) to nearly all (Figure 8B) of the river dolphin character states involved in prey capture evolved on internal branches that are optimized as marine - five out of ten state changes for the parsimony trees and nine out of ten on the ML/Bayesian constrained trees. Focusing on the parsimony trees, those changes that may have occurred in freshwater cetaceans are not evenly distributed; three occur on the terminal branch leading to *Platanista *and two others are positioned on the branch leading to *Lipotes*. Those same character states evolved in marine relatives of *Inia*, so even here the adaptation hypothesis is contradicted in part. Futhermore, one of the characters that is placed on the branch leading to *Lipotes*, a long mandibular symphysis, may have evolved earlier because an elongate symphysis occurs in the unsampled, extinct *Parapontoporia pacifica *[50], possibly a lipotid. To summarize, we find meager support for the hypothesis that prey capture features shared by river dolphins are adaptations to freshwater environments. A better understanding of the evolution of prey capture features in river dolphins will require observational data on the function(s) of these features in extant taxa, which is generally lacking, as well as paleobiological studies on extinct taxa to infer their diets and behaviors. Until this is done, such discussions will be largely speculative, and existing data cannot discriminate between the hypothesis that these prey-capture features are exaptations (sensu [108]) in extant river dolphins or are simply feeding adaptations that are equally effective in marine and fluvial environments. Characteristics of the soft anatomy shared by extant river dolphins (e.g., small eye size, broad forelimb flippers; Figure 9) may represent adaptations to life in a riverine habitat, but this hypothesis is difficult to test using fossil data and is again contradicted, or at least complicated, by the presence of these features in the coastal marine genus, *Pontoporia*.

## Conclusions

1. The relationships among extant lineages of Cetacea were investigated through phylogenetic analyses of diverse molecular data and parsimony analysis of the entire supermatrix, which included molecules, morphology, and extinct taxa. Of the 26 nodes defining relationships among extant taxa, support for one third of them is overwhelming, with ML bootstrap of 100%, PP of 1.0, and ddBS > 100 steps: Cetacea plus Hippopotamidae, Cetacea, Mysticeti, Balaenopteroidea, Balaenopteridae, Physeteroidea, Delphinida, Inioidea, and Phocoenidae. Another seven clades have very strong support, with ML bootstrap of 100%, PP of 1.0, and ddBS values between 100 and 20 steps: Odontoceti, Synrhina, Ziphiidae, Delphinoidea, Monodontoidae, Delphinidae, and Delphininae. Although there was broad congruence among these analyses, *Lipotes *and *Platanista*, two of the river dolphin genera, were inconsistently positioned in parsimony and explicitly model-based searches. Therefore, the tree supported by Bayesian and ML analyses of the molecular partition was used as a topological scaffold in an additional analysis of the morphological partition to determine how this underlying topology influences phylogenetic interpretations of extinct taxa. The ML/Bayesian constrained tree and the parsimony supermatrix tree suggest that the phylogenetic relationships of many extinct OTUs are unstable due to missing character data and to the density of taxonomic sampling, but several fossils were consistently positioned relative to extant taxa.

2. All trees with extinct taxa had a statistically significant fit with the fossil record, suggesting that the fossil record of crown Cetacea is quite good. Of the 25 described and undescribed Oligocene cetaceans included in our analyses, all were positioned outside of crown Odontoceti and crown Mysticeti. Thus our results do not support the hypothesis that the basal splits in both crown groups occurred in the late Eocene to early Oligocene as proposed by several molecular clock studies. However, we could not statistically reject some suboptimal topologies that placed Oligocene odontocetes within crown Odontoceti. Futhermore, additional Oligocene taxa need to be included in future combined phylogenetic analyses of molecular and morphological data - most notably *Ferecetotherium kelloggi*, *Notocetus marplesi*, *Oligodelphis azerbajdzanicus*, *Mauicetus parki*, and several undescribed specimens.

3. By allocating multiple, extinct, marine odontocetes to clades that include extant river dolphins, our analyses support the hypothesis that marine odontocetes invaded river systems on multiple occasions. Extant river dolphins share a suite of morphological features associated with raptorial prey capture. Some of these character states are symplesiomorphic whereas others may be convergent, depending upon the topology accepted and on alternative equally parsimonious optimizations. On the ML/Bayesian constrained trees, most if not all of these states evolved in marine lineages, whereas on the trees derived from a parsimony analysis of the supermatrix, at least half of these characters evolved in marine taxa. Even on the latter trees, no river dolphin character associated with prey capture is interpreted to have evolved separately on the three terminal branches leading to extant freshwater taxa. Thus we find little support for the hypothesis that these characters are adaptations to river environments.

## Methods

### Morphological Data

The morphological dataset incorporated in the present study is an expanded version of that published by Geisler and Sanders [2] and is composed of 304 phenotypic characters (Figure 3; Additional file 3) scored from 29 extant and 45 extinct operational taxonomic units (OTUs; Table 1). The primary difference in the new version is the addition of 17 taxa, including seven extant delphinids (*Delphinus delphis*, *Leucopleurus acutus*. *Globicephala macrorhynchus*, *Grampus griseus*, *Pseudorca crassidens*, *Orcinus orca*, *Orcaella brevirostris*), two extinct toothed mysticetes (*Mammalodon colliveri*, *Janjucetus hunderi*), the extant balaenopterid mysticete *Megaptera novaeangliae*, the archaic odontocete *Simocetus rayi*, the extinct ziphiid *Ninoziphius platyrostris*, the extant phocoenid *Phocoenoides dalli*, the extinct inioid *Pliopontos littoralis*, the kentriodontid *Atocetus nasalis*, and the extinct delphinoid *Albireo whistleri*. *Parapontoporia*, a fossil delphinidan that has been interpreted as a lipotid [2,8] or a pontoporiid [50], was scored at the genus level in a previous matrix [2], but was coded as two separate species, *P. wilsoni *and *P. sternbergi *in the present study. Character codings for *Mammalodon colliveri *and *Janjucetus hunderi *came directly from Fitzgerald [46]. Scorings for *Megaptera novaeangliae *were based on a mixture of published data and new observations, whereas the vast majority of scorings for the other added taxa were based on observations made directly from specimens. In addition, several codings were modified for the extinct odontocete *Brachydelphis mazeasi*. In Geisler and Sanders [2] this taxon was coded from a published description [109], but here, direct observations of the holotype and referred specimens were recorded (Additional file 3).

### Compilation and Alignment of Molecular Data

Sampling of molecular data was guided by the set of extant taxa coded for morphology (above). Molecular data were compiled for 20 of the extant species in the morphology dataset: *Bos taurus*, *Sus scrofa*, *Tursiops truncatus*, *Leucopleurus acutus*, *Grampus griseus*, *Pseudorca crassidens*, *Orcinus orca*, *Orcaella brevirostris*, *Phocoena phocoena*, *Phocoenoides dalli*, *Inia geoffrensis*, *Pontoporia blainvillei*, *Lipotes vexillifer*, *Tasmacetus shepherdi*, *Ziphius cavirostris*, *Physeter macrocephalus*, *Megaptera novaeangliae*, *Balaenoptera physalus*, *Eschrichtius robustus*, and *Caperea marginata *(Figure 3). To reduce missing data in the molecular matrix for the nine remaining species coded for morphology (*Hippopotamus amphibius*, *Delphinus delphis*, *Globicephala macrorhynchus*, *Delphinapterus leucas*, *Mesoplodon europaeus*, *Berardius bairdii*, *Platanista gangetica*, *Kogia breviceps*, *Eubalaena glacialis*), we made assumptions of monophyly and combined sequences from several species for a particular OTU. For example, *Hippopotamus amphibius *was coded for morphological characters, but for many genes that we sampled, molecular data have not been generated for this hippopotamid species. However, a close relative in the family Hippopotamidae, *Choeropsis liberiensis*, has been sequenced for some of these genes. So, we assumed the monophyly of Hippopotamidae in our molecular sampling and included some genes that have been sequenced from *Choeropsis *and other genes that have been sequenced from *Hippopotamus *in a single, composite OTU - Hippopotamidae. The nine groups that were assumed to be monophyletic in the molecular matrix were: Hippopotamidae (*Hippopotamus *+ *Choeropsis*), *Delphinus*, *Globicephala*, Monodontidae (*Monodon *+ *Delphinapterus*), *Mesoplodon*, *Berardius*, *Kogia*, *Platanista*, and Balaenidae (*Balaena *+ *Eubalaena*) (Table 1).

For compilation of molecular data, our starting point was a recently published supermatrix for Cetacea that includes transposon insertion events, mt genome data, and information from 45 nu loci that had been published prior to 8/2008 [20]. For the 29 extant taxa included here (Table 1), McGowen et al.'s [20] matrix of 42,335 characters was augmented by adding subsequently published mt and nu DNA sequences as well as newly generated DNA sequence data (Figure 3). The cutoff for inclusion of information from Genbank was 9/2009. Genes added to the supermatrix included segments of *BGN*, *CSN3*, *GZMA*, *HLA-DQA1*, *HOXC8*, *MC1R*, *MOS*, *RHO*, *RNASE1*, *UBE1Y7*, *ZP3*, 11 olfactory receptor loci, two anonymous Y chromosome loci, and mt tRNA genes. Seventy-two new sequences from 14 nu genes (*AMBN*, *ATP7A*, *BDNF*, *BTN1A1*, *CSN2*, *CSN3*, *ENAM*, *FGG*, *OR1I1*, *PRM1*, *RAG1*, *RNASE1*, *SRY*, *ZP3*) were generated in our labs for this study using the general PCR, cloning, and sequencing methods described in Gatesy et al. [110] and O'Leary and Gatesy [111]. Six new nu gene fragments from *Lipotes *were amplified and sequenced here (*ATP7A*, *CSN2*, *PRM1*, *RAG1*, *RNASE1*, *SRY*). Published primers were used for the *AMBN*, *ATP7A*, *BDNF*, *BTN1A1, CSN2*, *CSN3*, *ENAM*, *FGG*, *PRM1*, *RAG1*, *RNASE1*, and *ZP3 *genes [44,88,110-116]. PCR/sequencing primers for the *OR1I1 *gene were (5' to 3'): ORTHOCF - CAACCTGTCCCTGGTCGACG and ORTHOCR - CATTTGACCTGAGCAGAAAGG. PCR/sequencing primers for the *SRY *gene were (5' to 3'): TGAAGCGACCCATGAACG and TCGACGAGGTCGATACTT. Cetacean genomic DNA samples were provided by P. Morin, A. Dizon, and K. Robertson (SWFSC: Southwest Fisheries Science Center, NOAA, La Jolla, CA), G. Braulik (World Wildlife Fund), H. Rosenbaum (NYZS: New York Zoological Society), M. Milinkovitch (Free University of Brussels), M. Heide-Jørgensen (Greenland Institute of Natural Resources), The Marine Mammal Center - Sausalito, Smithsonian Institution - Division of Mammals, South Australian Museum. Donating institutions/persons and sample reference numbers for SWFSC are listed after each species in Supplementrary Table 1; all newly generated sequences were deposited in Genbank (Accession #s JF504739, JF504761, JF504780, JF504809, JF504952, JF504967, JF504975, AY442934, AY954636, AY954640, AY954641, AY954643, AY954645, AY954646, JF701623-JF701674). *AMBN *exon 6 data (five new sequences) were below the length limit accepted by Genbank (131 nucleotides), but these data can be retrieved from our supermatrix that is stored at MorphoBank.

Recently deposited data from Genbank and new sequences from our lab generally were aligned by eye to the previously published matrix [20] with the introduction of very few new gaps. However, several gene segments present in the McGowen et al. [20] matrix were re-aligned after addition of our newly generated data using CLUSTAL [117] with a gap opening penalty of ten and a gap extension cost of one; some adjacent gaps in the resulting multiple-sequence alignments were consolidated using SeqApp 1.9a [118] as in Gatesy et al. [110], O'Leary and Gatesy [111], and Spaulding et al. [88]. All newly-incorporated loci (e.g, *CSN3 *and *HOXC8 *that were not present in the matrix of McGowen et al. [20]) also were aligned using CLUSTAL and SeqApp. Indels (insertions or deletions) were coded for each gene in SeqState [119] using the simple gap-coding method of Simmons and Ochoterena [120]. The final molecular dataset was composed of 60,851 characters (transposons = 101, mtDNA = 15,587, nu DNA = 44,224, indels = 939) and exceeded that of McGowen et al. [20] by 18,516 characters. In sum, the matrix included segments of 69 nu loci (Figure 3); the genomic position of each nu sequence was determined by BLAST searches against the *Bos taurus *genome (version 4.0) and additional sequences in Genbank.

### Compilation of Supermatrix (Combined Morphology and Molecules)

The molecular dataset (60,851 characters; 29 extant taxa) was merged with the morphology dataset (304 characters; 29 extant taxa and 45 extinct taxa) into a single, concatenated supermatrix. Extinct taxa were coded as missing (?) for all molecular characters. The final combined dataset was submitted to Morphobank [121]; Genbank numbers for published DNA sequence data are recorded in this archived matrix. Additionally, species representatives for taxa assumed to be monophyletic are given for each data partition in the Morphobank supermatrix.

### Phylogenetic Analyses of Morphological Data

Parsimony analyses were conducted using the computer application TNT [122], with search parameters defined by the defaults under "New Technology Search" except that the number of times that minimum length is recovered was set at 1000. Observed similarities among states within single multistate characters were included as data in our analyses by ordering those characters, as suggested by Wilkinson [123]. If all character states were equally similar/dissimilar, then the multistate character was treated as unordered. Characters were assigned weights following a recent analysis that combined morphological and molecular data [124]. As in that study, between-character scaling (sensu Wiens [125]) was achieved by down-weighting ordered, multistate, morphological characters so that they have the same minimum length as a binary character, one step. If this is not done, then ordered characters with a large number of states would have a disproportionate influence on phylogenetic results by strongly penalizing trees that place taxa with disparate character states adjacent to one another [125]. Unordered multistate characters do not exhibit this behavior, thus they were given the same weight as binary characters. Although parsimony does not account for homoplasy on long branches when evaluating phylogenetic hypotheses, the parsimony method of implied weighting adjusts for homoplastic characters by down-weighting them dynamically during analysis [40]. Thus the morphology partition also was analyzed with implied weights as implemented in TNT, with the constant of the weighting function set to the default value (k = 3). A Bayesian analysis of the morphological partition also was executed using MrBayes 3.1.2 [126]. Unlike our parsimony analyses, we were unable to incorporate ordered characters in our Bayesian analysis because MrBayes was not able to accomodate the large number of states for some ordered characters. Instead, the Mk model with gamma rate variation was implemented, and all characters were treated as unordered. The Bayesian analysis of morphology was run as described below for the Bayesian analyses of molecular data.

Morphological characters were optimized onto trees by parsimony to diagnose clades and to track the evolution of characters shared by extant river dolphins and *Pontoporia*. PAUP 3.1.1 [127], PAUP* 4.0 [128], and MacClade [129] were used to map characters onto our trees and to distinguish equivocal versus unequivocal character transformations. All most parsimonious state reconstructions were estimated using MacClade.

### Phylogenetic Analyses of Molecular Data

Parsimony searches of the molecular data were done in PAUP* [128]. Two subsets of characters were analyzed in order to assess the influence of the large, rapidly evolving mt DNA partition on phylogenetic results: 1) nu data - including nu DNA sequences and insertions of transposons, and 2) all nu and mt characters combined. Each matrix was analyzed with and without indel characters; all character state transformations were given equal weight. Searches were heuristic with 1000 random taxon addition replicates and TBR branch swapping. Bootstrapping of characters was used to summarize support; 1000 replicates were executed for each bootstrap analysis, and each replicate included a heuristic search with 10 random taxon additions and tree bisection reconnection (TBR) branchswapping.

Bayesian analyses were run in MrBayes 3.1.2 [126] using the Cyberinfrastructure for Phylogenetic Research (CIPRES) Portal 2.0 [130]. Data were partitioned as in the above parsimony searches (nu, mt + nu), and analyses were run with and without indel characters. The binary model was used for transposon insertion events and indel characters. All sequence partitions were executed with a GTR + I + Γ model of evolution, as determined by the Akaike Information Criterion (AIC) via MrModeltest 2.2 [131]. Mt and nu sequence data were partitioned in the analysis of mt + nu data (separate models with a rate multiplier for branch lengths) as in McGowen et al. [20]. For each Bayesian analysis, two concurrent runs of 20 million generations were conducted with trees sampled every 1000 generations. Stationarity of likelihood scores was assessed using Tracer v1.04 [132]; split frequencies of runs were evaluated with "Are We There Yet?" (AWTY [133]). Using these assessments, the first 10% of trees was discarded as "burn-in." A 50% majority-rule consensus of post "burn-in" trees from concurrent runs was erected to summarize PPs for all clades. An additional Bayesian analysis was executed in which each "gene" in the overall molecular dataset was permitted to have a unique model of evolution; in this run, mtDNA was treated as a single linkage group. Results for this more finely partitioned and parameter-rich analysis were identical to those for our Bayesian run in which the data were divided into mt and nu partitions.

Maximum-likelihood analyses (ML) were conducted in RAxML 7.2.3 [134,135] using CIPRES. The following analyses were done: nu DNA sequences and all DNA sequences combined. For the ML analysis in which all sequence data were included, mt and nu data were separated to permit independent modeling of nucleotide evolution. Searches were conducted using standard default parameters of the GTRMIX option, which uses a GTR + Γ model of evolution. To assess nodal support, 200 bootstrap replicates were simultaneously executed in RAxML [135].

A division of the molecular dataset into more subpartitions (codon positions, exons, introns, etc.) was not attempted. The overall molecular supermatrix assembled for this study is quite complex and includes a wide array of molecular data, including mt 1^st ^codons, mt 2^nd ^codons, mt 3^rd ^codons, mt stop codons, mt intergenic regions, mt rDNA stems, mt rDNA loops, mt tRNA stems, mt tRNA loops, mt regions where two protein coding genes overlap (and where codons cannot be classified as 1^st^, 2^nd^, or 3^rd ^because a particular site might be a 1^st ^codon for one mt gene and a 2^nd ^codon for another mt gene), mt regions that are protein coding in some taxa but not in others (because the position of the stop codon has shifted during evolutionary history), nu 1^st ^codons, nu 2^nd ^codons, nu 3^rd ^codons, nu stop codons, nu introns, splice sites in nu introns, nu pseudogenes, nu regions that are protein coding in some taxa and are pseudogenic in others (e.g., enamel specific genes in toothed and toothless whales; some of the olfactory receptor loci), nu 5' noncoding regions, nu 3' noncoding regions, multiple members of particular nu gene families, positively selected genes (e.g., *PRM1*, *MCPH1*, and milk caseins), negatively selected genes (most mt and nu genes), and so on. Previous efforts at resolving cetacean phylogeny (Figures 1, 2) have not been nearly as inclusive as our supermatrix, and this separates our analysis from previous studies. Rather than excluding much molecular data so that fewer, well-defined molecular partitions remain (e.g., previous analyses of mt genomes that have limited analysis to only protein coding regions that do not overlap with each other and are encoded by the same DNA strand), we attempted to include as much of the available systematic data for Cetacea as possible (including morphological and paleontological information) and have not finely partitioned our matrix. Instead, we divided the data into mt and nu datasets or by gene, and have used a rate multiplier and the gamma distribution to account for rate variation among data partitions and sites within a given partition. Our approach to resolving cetacean phylogeny resulted in a very long DNA sequence alignment with much missing data (see Figure 3), but we feel that this framework is the best way to summarize all of the available character evidence that is relevant to relationships among cetaceans. Studies that fill missing data entries in our supermatrix, that attempt more complicated partitioning schemes, or take a coalescence approach [136] will provide future tests of the phylogenetic hypotheses presented in our study.

### Simultaneous Analyses of Molecular and Morphological Data

A Bayesian analysis of the complete supermatrix was attempted. Unfortunately the search could not be completed, likely due to the difficulty in placing relatively incomplete fossil taxa, the very large size of the supermatrix, missing molecular data (Figure 3), and/or difficulties in optimization of branch lengths. After 30 million generations, concurrent runs had failed to converge, and there was little resolution within each run. As an alternative to a Bayesian analysis of the combined matrix, the morphological partition was analyzed using parsimony but with relationships among extant taxa constrained to fit the topology obtained by ML/Bayesian analyses of the molecular partition (also known as a "backbone constraint" or "molecular scaffold;" see [137]). Homoplastic morphological characters were down-weighted in the constrained analysis using implied weighting as implemented in TNT (k = 3) [40].

Parsimony was utilized as the primary method of analysis for the supermatrix of morphological and molecular data. Analyses were executed in TNT and checked using PAUP* (see above). Characters were weighted as in the combined analysis of Seiffert [124]; character state transformations in ordered, multistate characters were downweighted so that these characters had the same minimum length as binary characters. For both molecular and morphological partitions, character state changes in unordered multistate characters were given the same weight as transformations in binary characters. Indels were coded as described above.

To quantify the character evidence for particular clades supported by parsimony analysis of the supermatrix, branch support (BS) [48] and double decay branch support (ddBS) [47] were estimated. Calculation of these indices involves finding the shortest trees that lack a particular clade of interest. To recover these suboptimal trees, a TNT module written by P. Goloboff was utilized http://tnt.insectmuseum.org/index.php/Scripts; this procedure automatically calculates BS using constrained searches. Defaults of the module were employed, except that 100 replicates for each constraint search were conducted.

For the supermatrix of extant and extinct taxa (74 OTUs), BS was first calculated for all nodes supported by the combined parsimony analysis. The supermatrix is characterized by extensive missing data, especially for fossils that can only be coded for a subset of the morphological partition and for none of the molecular characters. Therefore, we executed additional analyses and focused our assessments of character support on relationships among extant cetacean taxa. To measure BS for relationships among living species within the context of the fossil data, ddBS analyses [47] were executed. Backbone constraint trees [127] that defined relationships among extant taxa were used to estimate the minimum number of extra character steps required to disrupt these relationships. The constraint trees did not include any of the extinct taxa, but all extinct taxa were utilized in analysis. The phylogenetic placements of extinct taxa relative to the extant taxa were not fixed, so fossils were allowed to "float" in constrained tree searches [47]. Only the differential costs of contrasting relationships among extant taxa, irrespective of the positions of extinct taxa, were noted. These length differences, ddBS, were calculated for all monophyletic groupings of extant taxa supported by the 74-OTU supermatrix.

### Testing Temporal Implications of Phylogenetic Hypotheses

Analyses were conducted to determine the fits between trees supported by the complete supermatrix and the geologic record. The geologic ranges of extinct taxa sampled in the morphology partition are listed in Table 2 (also see Additional file 1: Table S2). To compile this table, records of sampled species were downloaded from the paleobiology database http://paleodb.org/. Many of the cetacean records on this database were entered as a result of previous studies on changes in cetacean diversity [138,139]. The records were then culled to remove fossil remains of questionable identity (described as "aff.", "cf.", or "?"). The stratigraphic ranges of undescribed OTU's from the Charleston Museum vertebrate paleontology collection are from Geisler and Sanders [2]. Focusing on species records is a conservative approach, which in some cases may lead to an underestimate for the range and first appearance datums (FADs) of higher-level clades. However, we were unwilling to assume monophyly of extinct clades that were not explicitly tested in our phylogenetic analyses.

The highest temporal resolution available for many records downloaded from the paleobiology database is stage, although stratigraphic provenance is usually reported as well. In some cases (e.g. Calvert Group), subsequent geologic studies have provided better constraints on the ages of cetacean-bearing geologic units (e.g, [140]), and these improved age estimates were used when available. Estimates for FADs and LADs (last appearance datums) are averages of the oldest and youngest ages for the smallest reported stratigraphic interval (Table 2; Additional file 2: Table S2). In cases where the duration of a stratigraphic unit is unknown, the age of the OTU is the same as the average of the uncertainty for the age of that unit. Although some extant species have been found in sediments of Pleistocene age, given the uncertainty of the ages of these sediments and the magnitude of the 41 million year record covered by the extinct taxa sampled, the ages of all extant taxa were set to the present day.

To determine the degree of fit between the geologic record, as listed in Table 2, and phylogenetic hypotheses, the modified Manhattan stratigraphic measure (MSM*) [141] and the gap excess ratio (GER) were calculated [142]. Both measures reflect required ghost lineages, which is the amount of extra time a lineage is assumed to have existed based on an earlier appearance of its sister-group [143]. Each measure treats time as an irreversible character, in the current case, a 17 state character for each unique FAD. MSM* is comparable to the consistency index of the stratigraphic character whereas the GER is comparable to its retention index [144]. As with those indices, the MSM* and GER are scaled to range between 0 and 1, with higher scores indicating fewer ghost lineages and a better fit between phylogeny and the geologic record. The significance of this fit was assessed using the method of Siddall [145]. The GER, MSM*, and the significance of the latter were calculated using TNT [122] with a script provided by D. Pol.

In all phylogenetic analyses of the supermatrix, Oligocene OTU's were excluded from crown Odontoceti and from crown Mysticeti in all minimum length trees (see Results). To assess the significance of these results, sequential ddBS analyses were conducted on two nodes, crown Odontoceti and crown Mysticeti. A total of 25 separate analyses were run, one for each extinct Oligocene OTU, using the supermatrix with character weights as described above. In each analysis, the backbone constraint tree included all extant taxa and one extinct taxon. If an extinct stem odontocete was included, then a search was conducted for the shortest tree that did not include a monophyletic crown Odontoceti. Similarly, if an extinct stem mysticete was included, then a search was conducted for the shortest tree that did not include a monophyletic crown Mysticeti. Using this procedure, the shortest trees that placed Oligocene OTU's inside these crown groups were recovered. To determine the ddBS, the length of minimum length trees (which had Oligocene taxa outside of these crown groups) was subtracted from the length of the suboptimal trees that had Oligocene taxa within the crown clade. The magnitude of ddBS scores provides a measure of the degree to which the supermatrix contradicts these suboptimal topologies; following Lee [146], Templeton/Wilcoxon rank sum tests [147] were conducted on each pair-wise comparison between a suboptimal topology and each minimum length tree to assess the significance of ddBS values. Winning-sites test were also conducted on the same pairwise comparisons [148]; both statistical tests were performed in PAUP* [128].

## Authors' contributions

All authors contributed to the research plan executed in this collaborative research. JHG collected all new morphological data, analyzed the morphological partition, executed most of the parsimony analyses of the supermatrix including all branch support calculations and character optimizations, conducted all analyses that tested temporal implications, and took the lead in writing the manuscript. MM collected new molecular sequences, compiled molecular data from Genbank, assisted in sequence alignment, and conducted all Bayesian/ML analyses. GY collected all new nu DNA sequences from the Yangtze River dolphin, as well as additional DNA sequence data. JG wrote several sections of the text, generated new DNA sequences, compiled molecular data from Genbank, executed sequence alignments, was responsible for final integration of data into the supermatrix, conducted parsimony analyses of molecular data and the supermatrix, and drafted the figures. All authors contributed to the text of the manuscript, have read it in its entirety, and approved its final version.

## Appendix

Appendix 1. Definitions, etymology, and morphological diagnoses for new clade names.

Plicogulae, new clade name, unranked.

Definition: Plicogulae refers to the least inclusive clade within Mysticeti that includes the most recent common ancestor of *Caperea marginata*, *Balaenoptera physalus*, and *Eschrichtius robustus*. This is a node-based definition that can be abbreviated as <*Caperea marginata *&*Balaenoptera physalus & Eschrichtius robustus*.

Etymology: Derived from Latin for "throats with grooves," referring to the grooves on the ventral side of the head and neck. In Balaenopteridae, these grooves allow for great expansion of the oral cavity during filter feeding [149].

Reference phylogeny: Figure 5 of the present study.

Composition: Based on Figure 5, Plicogulae includes Balaenopteridae, Eschrichtiidae, and *Caperea*. It specifically excludes Balaenidae, as well as the extinct mysticetes *Pelocetus *and *Diorocetus*, although we recognize that the exclusion of these two extinct genera is not strongly supported.

Morphological diagnosis: Plicogulae is diagnosed by the following features: zygomatic processes of the squamosal directed anteriorly (char. 142, state 0); lambdoidal crests of the occiput overhang the temporal fossa (153, 0); dorsal edge of tegmen tympani (i.e. superior process) is indistinct (232, 3); and longitudinal, external grooves on ventral side of head and neck (301, 1). All of these characters exhibit some homoplasy, and the diagnosis presented here is based on unequivocal synapomorphies that are shared between the tree in Figure 5 and the trees summarized in Figure 6.

Synrhina, new clade name, unranked.

Definition: Synrhina refers to the least inclusive clade within Odontoceti that includes the most recent common ancestor of *Platanista gangetica*, *Ziphius cavirostris*, and *Tursiops truncatus*. This is a node-based definition that can be abbreviated as <*Platanista gangetica *&*Ziphius cavirostris *&*Tursiops truncatus*.

Etymology: Derived from Classical Greek for "together nose", referring to the fact that the soft tissue nasal passages distal to the external bony nares are joined for nearly their entire lengths [4].

Reference phylogeny: Figure 5 of the present study.

Composition: Based on Figure 5, Synrhina includes Delphinidae, Phocoenidae, Monodontidae, Inioidea, Lipotidae, Ziphiidae, Platanistidae, Squalodelphinidae, Eurhinodelphinidae, *Kentriodon*, *Atocetus*, and *Albireo*. Although not included in our phylogenetic analysis, following the hypothesis of Muizon [8], this clade likely includes other "kentriodontids." Specifically excluded from this clade are Physeteridae, Kogiidae, Xenorophidae, *Simocetus*, and *Agorophius*.

Morphological diagnosis: Synrhina is diagnosed by the following characters: proximal ethmoid region exposed in dorsal view (char. 92, state 1); nasal passages are confluent immediately distal to external bony nares (95, 2); right soft tissue nasal passage is oriented dorsoventrally (96, 1); presence of blowhole ligament (101, 1), and presence of premaxillary sacs (105, 1).

Monodontoidae, new clade name, unranked.

Definition: Monodontoidae refers to the least inclusive clade within Odontoceti that includes the most recent common ancestor of *Monodon monoceros *and *Phocoena phocoena*. This is a node-based definition that can be abbreviated as <*Monodon monoceros *&*Phocoena phocoena*

Etymology: Derived from Classical Greek for "one tooth", referring to the single large tusk in male members of the species *Monodon monoceros*.

Reference phylogeny: Figure 2 of McGowen et al. [20]. The tree from that study is suggested as the reference phylogeny because members of Monodontidae were sampled at the species level, thus making the above definition easier to apply. For reasons described in the *Materials and Methods*, we included Monodontidae as a single OTU. Regardless, the minimum length trees for our supermatrix as well as those supported by McGowen et al. [20] yield the same composition for Monodontoidae.

Composition: Monodontoidae includes the families Monodontidae and Phocoenidae. According to Muizon et al. [150], this clade may also include *Odobenocetops*, but this hypothesis needs to be tested with computer-assisted phylogenetic analyses. Monodontoidae excludes Delphinidae and Inioidea.

Morphological diagnosis: Monodontoidae is diagnosed by the following characters: large exposure of fused lacrimal and jugal on roof of orbit (char. 55, 2); nasal bears fossa for posterior nasal sac (i.e. caudal sac) (117, 2); low lambdoidal crests of the occiput (153, 2); short anterior sinus, which is an extension of the pterygoid sinus system (157, 1); tympanosquamosal recess borders only part of the glenoid fossa (178, 2); and sternum composed of a single bone (290, 1). This list includes unambiguous, Monodontoidae synapomorphies that are shared by the parsimony and Bayesian constraint trees that we recovered in the present study (Figures 5, 6).

## Supplementary Material

Additional file 1 **Supplementary Figures**. Includes supplementary figs. 1, 2, and 3, which depict parsimony, implied weighting, and Bayesian trees for the morphological partition. Click here for file

Additional file 2 **Supplementary Tables**. Includes supplementary tables 1, 2, 3, and 4. These tables list sources of DNA and tissue samples, ages of all OTUs, results of analyses that measure the stratigraphic fits of the phylogenetic hypotheses we obtained, and details on some suboptimal trees. Click here for file

Additional file 3 **Morphological Character List and Observations**. Includes the list of 304 morphological characters, specimen examined and references consulted for the coding those characters, and comments on individual character codings. Click here for file
