# Supplementary material for: A supermatrix analysis of genomic, morphological, and paleontological data from crown Cetacea
Source: BMC Evol Biol. 2011 Apr 25;11:112. doi: 10.1186/1471-2148-11-112 (PMC3114740; doi:10.1186/1471-2148-11-112)
Supplement: Additional file 1 — Supplementary Figures. Includes supplementary figs. 1, 2, and 3, which depict parsimony, implied weighting, and Bayesian trees for the morphological partition. [file 1471-2148-11-112-S1.DOC]

Supplementary Material for:

A Supermatrix Analysis of Genomic, Morphological, and Paleontological Data from Crown Cetacea

Jonathan Geisler, Michael McGowen, Guang Yang, and John Gatesy

Includes:

Supplementary Figure 1 (Fig. S1) pg. 2

Supplementary Figure 2 (Fig. S2) pg. 3

Supplementary Figure 3 (Fig. S3) pg. 4

References for Supplementary Table 2. pg. 5

Supplementary Figure 1: Strict consensus of four minimum length trees, each 1743.78 steps in length, for the morphological partition of our dataset. This parsimony analysis did not employ implied weighting. Branch support scores are shown at internodes.

Supplementary Figure 2: Single optimal tree, score of 15019.78, obtained when the morphological partition of our dataset is analyzed under parsimony with implied weighting (k =3). Branch support scores are shown at internodes.

Supplementary Figure 3: 50% majority rule consensus of trees from Bayesian analysis of the morphological partition. Posterior probabilities are shown at internodes.

References Cited in Supplementary Table 2

Amiot R, Göhlich UB, Lécuyer C, de Muizon C, Cappetta H, Fourel F, Héran M-A, Martineau F: **Oxygen isotope compositions of phosphate from Middle Miocene–Early Pliocene marine vertebrates of Peru**. *Palaeogeography, Palaeoclimatology, Palaeoecology* 2008, **264**: 85–92.

Browning JV, Miller KG, McLaughlin PP, Kominz MA, Sugarman PJ, Monteverde D, Feigenson MD, Hernández JC: **Quantification of the effects of eustasy, subsidence, and sediment supply on Miocene sequences, mid-Atlantic margin of the United States**. *GSA Bulletin* 2006, **118**(5/6):567-588.

Daly E: **A middle Eocene *Zygorhiza* specimen from Mississippi (Cetacea, Archaeoceti)**. *Mississippi Geology* 1999, **20**(2):21-31.

del Rio CJ: **Tertiary marine molluscan assemblages of eastern Patagonia (Argentina): a biostratigraphic analysis**. *J Paleont* 2004, **78**(6):1097-1122.

Dockery, DT III: **Molluscan faunas across the Paleocene/Eocene series boundary in the North American Gulf Coastal Plain**. In *Late Paleocene-Early Eocene Climatic and Biotic Events in the Marine and Terrestrial Records*. Edited by Aubry M-P, Lucas SG, Berggren WA. New York: Columbia Univ. Press; 1998:296-322.

Dubrovo IA, Sanders AE: **A New Species of *Patriocetus* (Mammalia, Cetacea) from the Late Oligocene of Kazakhstan**. *Journal of Vertebrate Paleontology* 2005, **20**(3):577-590.

Fitzgerald EMG: **A review of the Tertiary fossil Cetacea (Mammalia) localities in Australia.** *Memoirs of Museum Victoria* 2004, **61**(2):183-208.

Fitzgerald EMG: **A bizarre new toothed mysticete (Cetacea) from Australia and the early evolution of baleen whales.** *Proc R Soc B* 2006, **273:**2955-2963.

Fitzgerald EMG: **The morphology and systematics of *Mammalodon colliveri* (Cetacea: Mysticeti), a toothed mysticete from the Oligocene of Australia**. *Zoological Journal of the Linnean Society* 2010, **158**:367-476.

Fordyce RE: ***Waipatia maerewhenua*, new genus and new species (Waipatiidae, new family), an archaic late Oligocene dolphin (Cetacea: Odontoceti: Platanistoidea) from New Zealand.** In *Contributions in Marine Mammal Paleontology Honoring Frank Whitmore Jr*. Edited by Berta A, Deméré TA. Proc. San Diego Soc. Nat. Hist., 1994, **29**:147-176.

Gradstein FM, Ogg JG, Smith AG: *A Geologic Time Scale 2004*. New York: Cambridge Univ. Press; 2005.

Harris WB, De Man E, VanSimaeys S, Vandenberghe N, Wampler, JM: **A comparison of glauconite K-Ar dates and ^87^Sr/^86^Sr dates from Rupelian and Chattian strata, Campine area, Belgium**. Abstracts of the International Geological Congress, 2008 [http://www.cprm.gov.br/33IGC/1314622.html]

Hulbert RC, Petkewich RM, Bishop GA, Bukry D, Aleshire DP: **A new middle Eocene protocetid whale (Mammalia: Cetacea: Archaeoceti) and** **associated biota from Georgia**. *Journal of Paleontology* 1998, **72**(5):907-927.

Kidwell SK: **Anatomy of extremely thin marine sequences landward of a passive-margin hinge zone: Neogene Calvert Cliffs succession, Maryland, U.S.A***. Journal of Sedimentary Research* 1997, **67**:322-340.

Lambert O: **Phylogenetic affinities of the long-snouted dolphin *Eurhinodelphis* (Cetacea, Odontoceti) from the Miocene of Antwerp, Belgium**. *Palaeontology* 2005, **48**(3):653-679.

Lambert O: **Sperm whales from the Miocene of the North Sea: a re-appraisal**. *Bulletin Institut royal des sciences naturelles de Belgique. Sciences de la terre* 2008, **78**:277-316.

Miller KG, Browning JV, Aubry M-P, Wade BS, Katz ME, Kulpecz AA, Wright JD: **Eocene-Oligocene global climate and sea-level changes: St. Stephens Quarry, Alabama**. *GSA Bulletin* 2008, **120**(1/2):34-53.

**Paleobiology Database** [http://paleodb.org/cgi-bin/bridge.pl]

Powell CL II, Barron JA, Sarna-Wojcicki AM, Clark JC, Perry FA, Brabb EE, Fleck RJ: **Age, Stratigraphy, and Correlations of the Late Neogene Purisima Formation, Central California Coast Ranges.** *U.S. Geological Survey Professional Paper* 2007, **1740**.

Prothero DR: **Pacific Coast Eocene-Oligocene marine chronostratigraphy: A review and an update**. In *From Greenhouse to Icehouse: The Marine Eocene-Oligocene Transition*. Edited by Prothero DR, Ivany LC, Nesbitt EA. New York: Columbia Univ. Press; 2003:1-13.

Prothero DR, Draus E, Cockburn TC, Nesbitt EA: **Paleomagnetism and counterclockwise tectonic rotation of the Upper Oligocene Sooke Formation, southern Vancouver Island, British Columbia**. *Can. J. Earth Sci.* 2008, **45**:499-507.

Sanders AE, Barnes LG: **Paleontology of the Late Oligocene Ashley and Chandler Bridge Formations of South Carolina, 3: Eomysticetidae, a new family of primitive mysticetes (Mammalia: Cetacea)**. In *Cenozoic mammals of land and sea: Tributes to the Career of Clayton E. Ray*. Edited by Emry RJ. *Smithsonian Contributions to Paleobiology* 2002, **93**:313-356.

Uhen MD: **New protocetid whales from Alabama and Mississippi, and a new cetacean clade, Pelagiceti**. *Journal of Vertebrate Paleontology* 2008, **28**(3):589-593.

Uhen MD, Fordyce RE, and Barnes LG: **Odontoceti**. In *Evolution of Tertiary Mammals of North America II*. Edited by Janis CM, Scott KM, and Jacobs LL. New York: Cambridge Univ. Press; 2008:566-606.

Weems R, Harris WB: **Major change in depositional style and paleoclimate in the Southeastern United States at or very near the Oligocene-Miocene boundary**. Abstracts of the International Geological Congress, 2008 [http://www.cprm.gov.br/33IGC/1323940.html]

Zeigler CV, Chan GL, Barnes LG: **A new late Miocene balaenopterid whale (Cetacea: Mysticeti), *Parabalaenoptera baulinensis* (new genus and species) from the Santa Cruz Mudstone, Point Reyes Peninsula, California.** *Proceedings of the California Academy of Sciences*1997, **50**(4):115-138.
