# Supplementary material for: A supermatrix analysis of genomic, morphological, and paleontological data from crown Cetacea
Source: BMC Evol Biol. 2011 Apr 25;11:112. doi: 10.1186/1471-2148-11-112 (PMC3114740; doi:10.1186/1471-2148-11-112)
Supplement: Additional file 3 — Morphological Character List and Observations. Includes the list of 304 morphological characters, specimen examined and references consulted for the coding those characters, and comments on individual character codings. [file 1471-2148-11-112-S3.DOC]

Supplementary Material for:

A Supermatrix Analysis of Genomic, Morphological, and Paleontological Data from Crown Cetacea

Jonathan Geisler, Michael McGowen, Guang Yang, and John Gatesy

Includes the following:

Specimens Examined and References Consulted pg.2

Character Descriptions pg. 6

References for the Above Sections pg. 31

Unedited Taxon Comments pg. 38

Unedited Character Comments pg. 41

SPECIMENS EXAMINED AND REFERENCES CONSULTED

Specimens and references were used to code three artiodactyl and 71 cetacean taxa for 304 morphological characters. In bold are taxa and specimens examined in the present study, whereas all other taxa and specimens were originally studied by and are listed in the appendix 1 of Geisler and Sanders (2003). For institutional abbreviations, see Materials and Methods. Insitutional abrreviations are as follows: **AMNH**, Department of Mammalogy and Division of Paleontology, American Museum of Natural History, New York, New York; **ChM**, Charleston Museum, Charleston, South Carolina; **GCM**, Georgia College and State University Museum, Milledgeville, Georgia; **GSM**, Georgia Southern Museum (vertebrate collection), Statesboro, Georgia; **LACM**, Natural History Museum of Los Angeles County, Los Angeles, California; **MNHN,** Muséum national d'Histoire naturelle, Paris, France; **USNM**, United States National Museum of Natural History, Departments of Vertebrate Zoology and Paleobiology, Washington, District of Columbia; **UCR**, Department of Geological Sciences, University of California Riverside;

*Terrestrial Artiodactyls*

*Hippopotamus amphibius*: AMNH 10668, 24282, 24289, 53774, 54248, 70019, 90302, 99637, 130247, 163821

*Sus scrofa*: AMNH 54507, 54508, 54638, 69422, 100260, 236144

*“Archaeoceti”*

*Georgiacetus vogtlensis*: GSM 350; Hulbert et al. (1998); Hulbert (1998)

*Zygorhiza kochii*: USNM 11962, 13773; Kellogg (1936)

*Mysticeti*

*Aetiocetus cotylalveus*: cast of USNM 25210 (DP); Emlong (1966); Barnes et al. (1994)

*Balaenoptera physalus*: USNM 16039, 504243, 504258, 504344; AMNH 35026; True (1904); Gambell (1985)

*Caperea marginata*: USNM 550146; AMNH 36692; Baker (1985)

*Chonecetus goedertorum*: cast of LACM 131146, Barnes et al. (1994)

*Diorocetus hiatus*: USNM 16783, 23100, 23494; Kellogg (1968)

*Eomysticetus whitmorei*: ChM PV4253; Sanders and Barnes (2002). Chandler Bridge Formation (Lower Chattian, Late Oligocene).

*Eschrichtius robustus*: USNM 364970, 571931; AMNH 34260, 181374; Andrews (1914); Wolman (1985)

*Eubalaena glacialis*: USNM 16435, 23077, 500860, 504886; AMNH 42752; True (1904); Omura et al. (1969); Cummings (1985)

***Janjucetus hunderi*: Fitzgerald (2006).**

***Mammalodon colliveri*: Fitzgerald (2006)**

***Megaptera novaeangliae*: USNM 21492; True 1904.**

*Micromysticetus rothauseni*: ChM PV4844; Sanders and Barnes (2002b). Ashley

Formation (Lower Chattian, Late Oligocene).

*Parabalaenoptera baulinensis*: Zeigler et al. (1997)

*Parietobalaena palmeri*: AMNH 128885

*Pelocetus calvertensis*: USNM 11976; Kellogg (1965)

Undescribed species: ChM PV5720; Barnes and Sanders (1996). Chandler Bridge

Formation (Lower Chattian, Late Oligocene).

Undescribed species: ChM PV2778; Barnes and Sanders (1996). Chandler Bridge

Formation (Lower Chattian, Late Oligocene).

*Odontoceti*

*Archaeodelphis patrius*: Allen (1921); R.E. Fordyce (pers. comm.)

*Agorophius* *pygmaeus*: ChM PV4256; Fordyce (1981)

***Albireo whistleri*: UCR 14589.**

*Atocetus nasalis*: LACM 63335, 30093.

*Berardius bairdii*: AMNH 331977; USNM 49726, 49727, 142118, 550153; True (1910b); Balcomb (1989)

*Brachydelphis mazeasi*: **MNHN PPI 121, 124;** Muizon (1988b)

*Delphinapterus leucas*: AMNH 10181, 34869, 34937, 34944, 77789, 173583, 180017, 185308, 207120, 212157, 212184, 212551, 212552, 212553; **USNM 16485, 22433, 23208, 275075;** Brodie (1989)

***Delphinus delphis:* get AMNH specimens; USNM 550228, 550240, 550305**

***Globicephala macrorhynchus*: USNM 22570, 22571, 22572, 37261, 500213, 550797; ChM 412**

***Grampus griseus*: USNM 24224, 504328, 550308, 550393, 550437, 550936**, **ChM 414**

*Inia geoffrensis*: AMNH 93412, 93413, 93414, **93415**, 93416, 95753, 98695, 209101, 209102, 209103, 209104, 209106; **USNM 395415**; Best and Silva (1989)

*Kentriodon pernix*: USNM 8060, 10670; Kellogg (1927). Geisler and Sanders (2003) included several additional specimens in their codings for this taxon; however, further study strongly suggests that some of the specimens in the USNM referred to *K. pernix* actually represent a different and undescribed species. Thus the codings here are restricted to two specimens that can confidently be assigned to *Kentriodon pernix*.

*Kogia breviceps*: AMNH 34867, 35680, 36595, 139684, 365912, 215172; Schulte (1917); Caldwell and Caldwell (1989)

***Leucopleurus acutus*: USNM 14243, 14279, 484916, 504196, 571390, 571447; GCM 936**

*Lipotes vexillifer*: AMNH 57333; Miller (1918); Peixun (1989)

*Mesoplodon europaeus*: AMNH 90051, 121894, 135639, 182649; USNM 23346, 360854; True (1910b); Mead (1989b)

***Ninoziphius platyrostris*: MNHN SAS 941; Muizon (1988b) CHECK**

*Notocetus vanbenedeni*: AMNH 9485; True (1910a)

***Orcaella brevirostris*: USNM 199743, 284429, 486170; Arnold and Heinsohn (1996).**

***Orcinus orca*: USNM 11980, 16488, 22068, 23004, 37166, 219326, 238112, 294406, 504925, 571360; ChM 433**

*Orycterocetus crocodilinus*: USNM 14730, 22926, 22930, 22931, 22953; Kellogg (1965)

*Patriocetus kazakhstanicus*: ChM PV4862 (cast); Dubrovo and Sanders (2000).

Karaginskaya Formation (Middle Chattian, Late Oligocene).

***Parapontoporia sternbergi*: LACM 6238; Gregory and Kellogg (1927); Barnes (1985)**

***Parapontoporia wilsoni*: LACM 125352; Barnes (1985)**

*Phocoena phocoena*: AMNH 10182, 10183, 10185, 10200, 10201, 36449, 36450, 134972, 185105, 185260, 212161, 212162; **USNM 218738, 218739, 572018, 572174, 572297, 572300**

***Phocoenoides dalli*: AMNH 12103, 90802; GCM 349; USNM 396304, 504969**

*Physeter macrocephalus*: AMNH 34872, 80206; USNM 395398, 550876; Flower (1869); Omura et al. (1962)

*Platanista gangetica*: AMNH 8461; **USNM 23456, 172409** (DM); Pilleri and Gihr (1976); Reeves and Brownell (1989)

***Pliopontos littoralis*: MNHN SAS 193, 931, 953.**

*Pontoporia blainvillei*: AMNH 205922, 235271, 235274, 254553, 254555, 254557, 254558, 254560; **USNM 48254, 49432, 482733, 482747**; Brownell (1989)

*Prosqualodon davidis*: AMNH 18601 (cast); Flynn (1948)

***Pseudorca crassidens:* USNM 11320, 20932, 23283, 218360, 484982, 501200; ChM 199**

***Simocetus rayi*: USNM 256517; Forydce (2002).**

*Squalodon calvertensis*: USNM 10484, 10949, 20902, 23537, 25910, 183055, 328343, 498743; Kellogg (1923b)

*Squaloziphius emlongi*: USNM 181528; Muizon (1991)

*Tasmacetus shepherdi*: USNM 484878; Mead (1989a)

*Tursiops truncatus*: AMNH 1774, 14485, 24111, 34994, 35418, 35419, 35424, 35427, 35547, 74885, 115648, 120920, 180808, 184930, 212554, 217685, 217686, 249111; Rommel (1990)

Undescribed species: ChM PV4746. Chandler Bridge Formation (Lower Chattian, Late Oligocene).

Undescribed species: ChM PV2758. Chandler Bridge Formation (Lower Chattian, Late Oligocene).

Undescribed species: ChM PV4834. Chandler Bridge Formation (Lower Chattian, Late Oligocene).

Undescribed species: ChM PV4834, PV5711. Chandler Bridge Formation (Lower

Chattian, Late Oligocene).

Undescribed species: ChM PV5852. Ashley Formation (Lower Chattian, Late

Oligocene).

Undescribed species: ChM PV2764. Chandler Bridge Formation (Lower

Chattian, Late Oligocene).

Undescribed species: ChM PV2761. Chandler Bridge Formation (Lower

Chattian, Late Oligocene).

Undescribed species: ChM PV4961. Chandler Bridge Formation (Lower

Chattian, Late Oligocene).

Undescribed species: ChM PV4802. Chandler Bridge Formation (Lower

Chattian, Late Oligocene).

*Waipatia maerewhenua*: Fordyce (1994)

*Xenorophus sloanii*: USNM 11049; Kellogg (1923a). Ashley Formation (Lower

Chattian, Late Oligocene).

*Xenorophus* sp.: ChM PV4823. Chandler Bridge Formation (Lower

Chattian, Late Oligocene).

*Xiphiacetus bossi*: USNM 8842, 10464, 10714; Kellogg (1925)

*Xiphiacetus* sp.: USNM 2361, 167629

*Zarhachis flagellator*: USNM 10485, 13768, 16633, 24868, 26134, 206006, 214759; Kellogg (1926)

*Ziphius cavirostris*: AMNH 244110, 40015, 40016, 70378; True (1910b); Heyning (1989b)

CHARACTER DESCRIPTIONS

(taken from Geisler and Sanders, 2003 with minor modifications)

*Rostrum, Dental, and Mandibular*

1. *Baleen*.—Absent (0); present (1) (Miller, 1923).

2. *Rostrum*.—Narrows in width anteriorly or anterior half approximately the same width as posterior half (0); anterior part widened transversely (1) (Muizon, 1994).

3. *Length of rostral portion of maxilla* (ordered).—Short, rostral portion of maxilla < 43% of condylobasal length excluding the premaxillae (0); intermediate, rostral portion between 48 and 70% of modified condylobasal length (1); elongate, rostral portion > 73% modified condylobasal length (2) (modified from Barnes, 1985).

4. *Anterior half of maxilla*.—Its lateral edge in cross section forms an angle of 60° to 45° (0); highly acute angle with flattened maxilla (1) (modified from Barnes, 1990).

5. *Vomerine trough, or mesorostral canal*.—Open, vomer in cross section is V-shaped or U-shaped (0); partially or completely filled in with bone, becomes solid rod of bone (1) (Moore, 1968).

6. *Rostral constriction well anterior to antorbital notch*.—Absent (0); present (1) (Barnes, 1985).

7. *Width of rostrum at antorbital notch* (ordered).—Wide, rostral width > 92% the width across of middle of orbits (0); fairly wide, between 82% and 72% the width across orbits (1); narrow, between 68% and 46% the orbital width (2); very narrow, between 32% and 29% the orbital width (3).

8. *Premaxilla in dorsal view*.—Portion adjacent to and anterior to nasal opening narrows or remains the same width anteriorly (0); widens at anterior end (1).

9. *Premaxillae on anterior two thirds of rostrum* (ordered).—With skull in dorsal view, contact along midline for most or entire length (0); sporadic contact along rostrum (1); separated by a narrow fissure for entire length (2); clear separation although mesorostral gutter still has a partial roof (3); very wide separation, mesorostral gutter is completely open along entire length (4) (modified from Muizon, 1988a; Fordyce, 1994; Messenger and McGuire, 1998).

10. *Suture between maxilla and premaxilla on rostrum* (ordered).—Suture fused along most of rostrum (0); anterior quarter of rostrum fused with remaining portions unfused (1); unfused along entire rostrum but articulation tight (2); suture is unfused and marked by a deep grooved (3) (modified from Fordyce, 1994; Messenger and McGuire, 1998).

11. *Posterior region of rostral edge* (ordered).—Lateral margin is straight or gently concave with skull in dorsal view (0); slightly bowed outward causing a V-shaped antorbital notch (1); bowed far outward forming a deep U-shaped antorbital notch (2); lateral margin of maxilla nearly contact lacrimal and jugal resulting in the opening of the notch being a narrow slit (3).

12. *Posterodorsal portion of maxilla*.—Sutured to frontal (0); not sutured, separated from frontal by a distinct gap, which is situated between the maxilla anterodorsal and the frontal ventrally (1) (McLeod et al., 1993).

13. *Steep face on anterolateral edge of zygomatic process of maxilla clearly separating it from rostral portion of maxilla* (ordered).—Absent (0); present but low (1); present and well developed (2).

14. *Posterior end of ascending process of maxilla*.—Tapers to a point (0); end is squared-off (1). Cannot be scored for taxa with maxilla covering most of supraorbital processes of the frontal.

15. *Posterior wall of antorbital notch*.—Maxilla (0); lacrimal and jugal (1).

16. *Palatal surface of rostrum*.—Flat or gently concave (0); bears pronounced longitudinal keel along the midline of the rostrum (1) (McLeod et al., 1993). The keel is formed by vomer and medial edges of maxillae

17. *Palatal surface of maxilla*.—Bears few vascular foramina, those that are present are small (0); bears many, large vascular foramina that open laterally and anterolaterally into long sulci (1); bears numerous small vascular foramina that lack sulci (2).

18. *Posterior end of palatal surface of rostrum at the suture between palatine and maxilla* (ordered).—Concave to flat, depth of rostrum, measured as the dorsoventral distance from the level of the lateral edge of rostrum to the ventral-most part of rostrum, is < 8% the width of rostrum at antorbital notches (0); convex, depth between 11% and 25% the rostral width (1); highly convex, rostral depth > 27% the rostral width (2). The convexity of the rostrum is a part of characters included by Barnes (1985) and Fordyce (1994).

19. *Palatine*.—Sutured to maxilla and suture visible (0); fused to maxilla (1) (Messenger and McGuire, 1998).

20. *Palatine/Maxilla suture*.—In ventral view, suture between both palatines and both maxillae is straight transversely or bowed anteriorly (0); maxillae have posterior processes that separate palatines anteriorly, suture around midline is V-shaped and points posteriorly (1).

21. *Teeth in females*.—Erupt in adulthood (0); do not erupt in adulthood but remain in the crypt (1) (Moore, 1968).

22. *Tooth rows*.—Separated and diverge posteriorly (0); left and right sides adjacent to the midline and thus close together, are nearly parallel (1) (Zhou, 1982).

23. *Number of double-rooted teeth in maxilla* (ordered).—None (0); 1 or 2 (1); 4 (2); 5 (3); 6 (4); 7 (5); 8 or more (6). The primitive state for Cetacea and Artiodactyla is probably state “3”; however, the character was coded with no teeth as state “0” in order to implement the ordering of states in the phylogenetic analyses. Muizon (1987, 1991, 1994) noted that the absence of all double-rooted teeth is a synapomorphy of Platanistidae plus Squalodelphinidae.

24. *Number of teeth with alveoli completely enclosed in the maxilla* (ordered).—None, 1, 2, or 3 (0); 7 to 8 (1); 9 to 10 (2); 11 to 13 (3); 15 to 17 (4); 21 to 23 (5); 26 to 29 (6); 32 to 39 (7); 50 to 60 (8) (modified from Messenger and McGuire, 1998). State “1” is probably the primitive condition for Cetacea and Artiodactyla; however, the character was coded with no teeth as state “0” in order to implement the ordering of states in the phylogenetic analyses. Barnes (1985) listed extreme polydonty as a synapomorphy of the genus *Parapontoporia*.

25. *Large diastemata between posterior buccal teeth*.—Absent (0); present (1).

*Teeth Morphology*

26. *Tooth enamel*.—Smooth (0); bears reticulating striae (1); nodular (2) (Zhou, 1982).

27. *Lower anterior mandibular teeth*.—Conical (0); spatulate (1); laterally compressed (2) (Messenger and McGuire, 1998). Heyning (1989b) described state “1” and Moore (1968) described state “2”.

28. *Lower anterior teeth*.—Deeply rooted with at least half of tooth forming root (0); not deeply rooted (1) (Flower, 1872; Moore, 1968).

29. *Accessory shelf on posterior teeth*.—Present (0); absent (1) (Flower, 1867; Messenger and McGuire, 1998).

30. *Posterior buccal teeth* (ordered).—High peg-shaped teeth, crown base is < 37% the crown height (0); nearly an equilateral triangle, crown base is between 100% to 148% the crown height (1); wide low teeth, crown base is > 180% the crown height (2).

31. *Buccal teeth ectocingulum*.—Absent (0); present (1).

32. *Buccal teeth entocingulum*.—Present (0); absent (1).

33. *Buccal teeth*.—Bear accessory cusps (0); cusps absent (1) (Kellogg, 1923b).

34. *Central cusp as compared to denticles*.—Much larger (0); subequal (1). Cannot be scored for taxa that lack denticles.

*Mandibular*

35. *Anterior-most mandibular teeth* (ordered).—Oriented anteriorly (0); vertical (1); inclined posteriorly (2) (Moore, 1968; Messenger and McGuire, 1998).

36. *Anterior-most mandibular teeth* (ordered).—Smaller than posterior teeth (0); approximately same size as posterior teeth (1); greatly enlarged (2) (modified from Flower, 1872; Heyning, 1989; Muizon, 1991; Messenger and McGuire, 1998).

37. *Number of* *teeth number in lower jaw* (ordered).—None (0); 1 (1); 2 (2); 8 to 9 (3); 11 to 12 (4); 13 to 14 (5); 20 to 23 (6); 24 to 27 (7); 28 to 34 (8); more than 40 teeth (9) (modified from Messenger and McGuire, 1998). State “3” is probably the primitive condition for Cetacea and Artiodactyla; however, the character was coded with no teeth as state “0” in order to implement the ordering of states in the phylogenetic analyses.

38. *Mandible in lateral view*.—Straight (0); arched dorsally (1) (McLeod et al., 1993).

39. *Length of mandibular symphysis* (ordered).—Short, mandibular symphysis forms less than 28% of the total mandibular length (0); long, symphysis length between 33% and 40% of the mandibular length (1); very long, symphysis forms more than 48% of the length of the mandibles (2) (modified from Heyning, 1989; Barnes, 1990).

40. *Mandibular symphysis* (ordered).—Fused (0); sutured but unfused (1); not sutured, connected by ligaments (2) (Fordyce, 1994). Barnes (1990) listed a loose mandibular symphysis as a synapomorphy of Mysticeti. State “1” is probably the primitive condition for Cetacea and Artiodactyla; however, the fused condition was designated as state “0” in order to implement the ordering of states in the phylogenetic analyses.

41. *Longitudinal groove on underside of mandible*.—Absent (0); present (1) (Miller, 1923).

42. *Mandible* (ordered).—Bowed medially (0); straight (1); slightly bowed laterally, a line drawn from the posteriormost to anteriormost points stays within body of mandible (2); strongly bowed outward, line from anterior to posterior points does not entirely lie within body of mandible (3) (Miller, 1923; Sanders and Barnes, 2002).

43. *Mandibular fossa*.—Small or absent (0); present and large, forms a large cavity posterior to mandibular foramen (1) (Barnes, 1990).

44. *Shape of coronoid process* (ordered).—Long and low, height of mandible at coronoid process < 89% the length of coronoid (0); height of mandible between 100% and 177% the length of coronoid (1); short and high, height between 203% and 300% coronoid length (2); very high and short, height > 450% the length of coronoid (3).

45. *Dorsal surface of condyle*.—Elevated above dorsal edge of the rest of mandible, not counting coronoid process (0); at same level as rest of mandible (1) (Sanders and Barnes, 2002).

*Orbit*

46. *Supraorbital processes of frontal*.—Are horizontal or gradually slope lateroventrally away from vertex of skull (0); abruptly depressed at base to a level noticeably below that of dorsal surface of interorbital region (1); slope laterodorsally away from vertex (2) (Miller, 1923; Messenger and McGuire, 1998).

47. *Dorsal edge of orbit relative to lateral edge of rostrum* (ordered).—Below the level of the edge of rostrum (0); orbit low, either in line with edge of rostrum or slightly above it, height of orbit < 46% the height of rostral base, both heights measured relative to the lateral edge of rostrum (1); orbit low, height of dorsal edge of orbit between 50% and 92% the rostral height (2); orbit high, height between 100% and 128% the rostral height (3); orbit elevated well above rostrum, orbital height > 163% the rostral height (4).

48. *Frontal/Maxilla suture*.—With skull in lateral view, suture is approximately horizontal, and lateral exposure of frontal over the orbit does not thicken posteriorly (0); angled posterodorsally at and angle of 50° to 70° from axis of rostrum, lateral exposure of frontal thickens posteriorly (1) (Miller, 1923). This character cannot be scored for taxa that lack overlap of the maxilla onto the frontal.

49. *Anterior edge of the supraorbital process* (ordered).—Oriented anteromedially (0); oriented slightly anterolaterally, forms an angle < 30° with sagittal plane (1); oriented anterolaterally, forms an angle between 35° and 60° (2); oriented anterolaterally or laterally, forms and angle between 68° and 90° degrees (3); oriented posterolaterally, forms an angle between 107° and 120° (4); oriented posterolaterally, forms an angle > 142° (5). The anterior edge of the supraorbital process participates in the formation of the antorbital notch. An antorbital notch opening anteriorly was listed by Barnes (1990) as a synapomorphy of Odontoceti.

50. *Lacrimal*.—Forms small bone on anterior edge of orbit with small orbital portion (0); enlarged both posteromedially and anterolaterally paralleling anterior edge of supraorbital process of frontal, shaped like a thick rod (1).

51. *Lacrimal* (ordered).—Restricted below supraorbital process of frontal (0); wraps around anterior edge of supraorbital process of frontal and slightly overlies its anterior end (1); greatly expanded posterodorsally and covering much of lateral side of supraorbital process of frontal (2) (modified from Miller, 1923; Kellogg, 1923a).

52. *Lacrimal foramen or groove*.—Present (0); absent (1).

53. *Lacrimal and jugal*.—Separate (0); fused (1) (Miller, 1923; Heyning, 1989).

54. *Jugal and lacrimal*.—Jugal and lacrimal contact each other externally (0); lacrimal excluded from edge of skull, jugal directly contacts anterior edge of frontal (1) (modified from Miller, 1923).

55. *Combined anteroposterior length of the lacrimal and jugal exposure that is posterior to antorbital notch* (ordered).—With skull in ventral view, exposure is small and combined length forms < 31% of anteroposterior distance from antorbital notch to postorbital ridge (0); intermediate, forms between 50% and 92% of that distance (1); large, forms between 62% and 69% that distance (2); very large, forms > 77% of that distance (3). The postorbital ridge is a curved ridge that extends from the postorbital process to the orbital foramen (Fordyce, 2002). It follows much of the course of the orbital nerve and demarcates the separation between the orbital and postorbital regions of the skull.

56. *Jugal*.—Thick and sturdy (0); thin splint or incomplete or absent (1) (Miller, 1923).

57. *Dorsolateral edge of internal opening of infraorbital foramen*.—Formed by maxilla (0); formed by maxilla and lacrimal and/or jugal (1); formed by lacrimal and/or jugal (2); formed by frontal (3) (modified from Miller, 1923).

58. *Ventromedial edge of internal opening of infraorbital foramen* (ordered).—Formed by maxilla (0); formed by maxilla and palatine and/or pterygoid (1); formed by palatine and/or pterygoid (2) (modified from Miller, 1923).

59. *Maxillary infraorbital plate*.—Absent (0); present but small (1), present and large (2) (Miller, 1923). The infraorbital plate of the maxillary is the posterior part of the maxilla underlying the orbit.

60. *Anteriormost point on the posterior edge of the supraorbital process* (ordered).—The anteriormost point is at the lateral edge of postorbital process (0); located laterally, between 70% and 74% of transverse distance from sagittal plane to the lateral edge of postorbital process (1); positioned approximately midway, located between 42% and 61% of that distance (2); medially positioned, located at a point < 34% of that distance (3).

61. *Postorbital process*.—Long and projects posterolaterally and slightly ventrally (0); short and directed ventrally (1).

62. *Postorbital ridge*.—Present, forms well-defined curved ridge on posterior edge of sulcus for optic nerve (0); no well-defined ridge, region is gently convex (1).

*Facial Region*

63. *Facial region of skull, skull in lateral view* (ordered).—Concave (0); flat (1); moderately arched dorsally (2); greatly arched dorsally (3) (Miller, 1923; Heyning, 1989; Messenger and McGuire, 1998).

64. *Infraorbital foramina* (ordered).—Single (0); two (1); three or more (2) (Barnes, 1984).

65. *Rostral basin*.—Absent or poorly defined (0); present, situated medial to antorbital notch and anterior to supraorbital process of frontal, best developed medially and ventrally where lateral edge of maxilla is very thin (1).

66. *Transverse distance between lateral edges of right and left premaxillae at antorbital notches* (ordered).—Small, distance < 48% the width of rostrum at antorbital notches (0); intermediate, distance between 52% and 64% the antorbital width (1); wide, distance > than 78% the antorbital width (2).

67. *Premaxillae immediately anterior to external bony nares* (ordered).—Widely separate with skull in dorsal view, gap between medial edges of premaxillae > 63% the maximum width of external bony nares (0); narrow separation, gap between premaxillae between 56% and 32% the width of external nares (1); separation absent or nearly so, gap < 28% the nares width (2).

68. *Premaxillae anterior to nasal openings* (ordered).—Are flat or concave, form a premaxillary sac fossa (spiracular plate) (0); convex transversely (1); form distinct bosses or “premaxillary eminencies” with steep posterior faces on anterior edges of nasal openings (2) (Muizon, 1988a; Barnes, 1990). This character was split into two characters by Messenger and McGuire (1998). They are combined here because a taxon with bosses cannot simultaneously have a fossa.

69. *Premaxillary foramina* (ordered).—Absent (0); present and one on right side (1); two on right side (2); three on right side (3) (modified from Barnes, 1990).

70. *Premaxillary foramen size* (ordered).—Right and left subequal (0); left larger than right (1); left much larger than right (2) (modified from Messenger and McGuire, 1998).

71. *Position of premaxillary foramen*.—Far anterior of antorbital notch and anterior edge of supraorbital process (0); approximately medial to or posterior to antorbital notch region, which is at the junction of supraorbital process with rostrum (1).

72. *Posterolateral sulcus from premaxillary foramen* (ordered).—Sulcus very short or absent (0); present and short (1); present and extends to level equivalent to middle of nasal openings (2) (modified from Muizon, 1988a).

73. *Premaxillae*.—Restricted to medial position adjacent to mesorostral canal and nasal opening (0); extended laterally covering much of the supraorbital process (1). The maxilla in turn overlies the premaxilla (Kellogg, 1923a).

74. *Posteriormost end of ascending process of premaxilla* (ordered).—Located just anterior to or in a transverse line with anterior edge of supraorbital process of the frontal (0); in line with anterior half of supraorbital process of frontal or halfway point, anteroposteriorly, of supraorbital process (1); in line with posterior half of supraorbital process or postorbital process of frontal (2); in line with gap between postorbital process and anterior tip of zygomatic process of the squamosal or in line with anterior tip of the latter process (3); in line with space between anterior tip of zygomatic process of squamosal and anterior edge of floor of the squamosal fossa or in line with anterior edge of floor of the squamosal fossa (4); located posterior to anterior edge of floor of the squamosal fossa (5).

75. *Maxillary foramen*.—Absent (0); present and one, situated over supraorbital process of frontal (1); two (2); foramina absent because roof of canal that carries posterior branches of internal maxillary artery and the maxillary division of infraorbital nerve is unossified (3) (modified from Barnes, 1990). The maxillary foramen (or foramina) is distinct from the facial opening(s) of the infraorbital canal.

76. *Maxilla* (ordered).—Abuts anterior edge of supraorbital process of frontal (0); partially covers supraorbital process (1); covers almost entire surface (2) (Miller, 1923; Fordyce, 1994). Fordyce erected state “1” for *Archaeodelphis* (Allen, 1921).

77. *Posteriormost edge of the ascending process of maxilla* (ordered).—Situated well anterior to anterior edge of orbit (0); in transverse line with anterior half of supraorbital process of frontal or in line with the halfway point, anteroposteriorly, of supraorbital process (1); in line with posterior half of supraorbital process or in line with postorbital process of frontal (2); in line with gap between postorbital process and the anterior tip of zygomatic process of squamosal or in line with anterior tip of the latter process (3); in line with space between anterior tip of zygomatic process of squamosal and anterior edge of floor of squamosal fossa or in line with anterior edge of the floor of squamosal fossa (4); posterior to anterior edge of floor of squamosal fossa (5).

78. *Anterolateral corner of maxilla overlying supraorbital process of frontal*.—Thin and equal in thickness to parts posteromedial (0); thickened with thinner maxilla in posteromedial direction (1). The thickened maxilla plus other nearby elevated structures (e.g. nasal opening, supraoccipital), delimit the edges of a broad fossa for the insertion of the maxillonasolabialis muscle.

79. *Maxillary ridge* (ordered).—Absent (0); present (1); form transversely compressed and high crest (2); crest arches over and encloses a cavity for the melon (3) (modified from Miller, 1923; Muizon, 1987). Messenger and McGuire (1998) split this into two characters; however, this results in scoring the absence of the crests twice.

80. *Anterior edge of nasals* (ordered).—In transverse line with incisors, canines, or intervening diastema (0); in line with P1 (1); in line with P2 or about 18% of the total rostral length towards anterior edge of rostrum (2); just anterior to or in line with anterior edge of supraorbital process of frontal (3); in line with anterior half of supraorbital process of frontal or in line with the halfway point, anteroposteriorly, of supraorbital process (4); in line with posterior half of supraorbital process or in line with postorbital process of frontal (5); in line with gap between postorbital process and the anterior tip of zygomatic process of squamosal or in line with the anterior tip of the latter process (6); in line with space between the anterior tip of zygomatic process of squamosal and anterior edge of the floor of squamosal fossa or in line with anterior edge of the floor of squamosal fossa (7); posterior to the anterior edge of the floor of the squamosal fossa (8).

81. *Anterior edge of nasal openings*.—V-shaped, premaxillae gradually converge anteriorly to the midline (0); U-shaped, premaxillae abruptly converge anteriorly to the midline (1) (Muizon, 1988a). Muizon (1988a) described state “0” as heart-shaped.

82. *Maxillae* (ordered).—In region anterior to nasal openings, maxillae are exposed lateral to premaxillae (0); maxillae are exposed at posterior end of roof of mesorostral gutter, medial to the premaxillae and nearly converge on midline (1); same as 1 except maxilla also exposed on anterior edge of nasal openings (2) (Muizon, 1988a).

83. *Ossicles*.—Absent (0); present, occur in anteromedial corners of nasal openings, probably a derivative of the maxilla (1) (Muizon, 1988a).

84. *Right premaxilla* (ordered).—Posterior edge approximately in line with posterior edge of left premaxilla (0); right premaxilla extended distinctly farther than left (1); right extended much farther than left (2) (modified from Barnes, 1990; Messenger and McGuire, 1998).

85. *Transverse width of* *right premaxilla immediately anterior to external bony nares* (ordered).—Distinctly narrower than left premaxilla (0); subequal, width of right premaxilla within 10% of the width of left premaxilla (1); right wider, width is between 130% and 145% the width of the left (2); right much wider, width > 167% the width of the left (3)..

86. *Right premaxilla*.—Portion posterior to nasal openings wider than portion anterior to opening, with nasal septum angled anteriorly and to the right (0); portion anterior wider than portion posterior to nasal opening, septum angled anteriorly and to the left (1). Cannot be scored for taxa that lack extension of the premaxilla posterior to the nasal openings or those that lack asymmetrical widening of the right premaxilla.

87. *Osseous external nasal openings*.—Left and right are the same size (0); left is twice or more the size of the right (1) (Barnes, 1990).

88. *Supracranial basin*.—Absent (0); present (1) (Heyning, 1989).

89. *Posterior end of premaxilla*.—Posterior end adjacent to lateral edge of nasal opening (0); angled slightly laterally resulting in the following sequence, from lateral to medial, in one transverse plane: premaxilla, maxilla, anterior edge of nasals or mesethmoid (1) (Muizon, 1988a; Heyning, 1989).

90. *Angle of premaxillae anterior to external bony nares, skull in lateral view* (ordered).—Low angle, premaxillae form an angle < 28° with the lateral edge of rostrum (0); intermediate angle, form an angle between 30° and 40° (1); high angle, form an angle > 45° (2) (modified from Moore, 1968).

91. *Premaxillae adjacent to nasal opening* (ordered).—Thin dorsoventrally and porous internally (0); pachyostotic, in direction perpendicular to face, and pachyosteosclerotic but nasals and premaxillae equally project dorsally and anteriorly (1); extreme pachyostosis, premaxillae adjacent to nasals project farther outward, anteriorly and dorsally (2) (modified from Moore, 1968).

92. *Proximal ethmoid region*.—Not visible in dorsal view, roofed over by nasals (0); exposed dorsally (1) (Miller, 1923).

93. *Mesethmoid*.—Forms T-shaped bone with median plate separating right and left nasal passages, not all of the dorsal part is divided by median plate (0); bears expanded posterodorsal plate which is not divided by median plate, median plate situated more ventrally (1) (modified from Muizon, 1984; Muizon, 1988a). This bone may include parts of the cribiform and perpendicular plates of the ethmoid.

*Facial Soft Tissue*

94. *Shape of soft tissue external nares*.—Crescent with apices pointed anteriorly (0); crescent with apices pointed posteriorly, might be skewed (1); rectangular (2); a longitudinal slit, might be slightly sigmoidal or angled (3); comma-shaped (4) (modified from Messenger and McGuire, 1998).

95. *Soft tissue nasal passages distal to bony external nares* (ordered).—Separate with two separate soft tissue external nares (0); separate for most of length but confluent just proximal to blowhole (1); confluent (2) (Heyning, 1989; Messenger and McGuire, 1998).

96. *Orientation of right soft tissue nasal passages*.—Oriented anterodorsally (0); oriented dorsally (1) (Messenger and McGuire, 1998). Cannot be scored for taxa that have little soft tissue in the facial area. In those taxa the bony and soft tissue external nares are in the same position.

97. *Right posterior dorsal bursa*.—Small (0); hypertrophied to form the spermaceti organ (1) (Cranford et al. 1996).

98. *Melon* (ordered).—Absent (0); small (1); hypertrophied (2) (Heyning and Mead, 1990).

99. *Distal sacs*.—Absent (0); present, situated immediately distal to museau de singe (1) (modified from Heyning, 1989). The vestibular sacs are considered homologous to the distal sacs of the right nasal passage of physeterids.

100. *Left and right distal sacs*.—Equal in size (0); right larger, includes physeterid condition of no distal sac on the left nasal passage (1) (Mead, 1975; Heyning, 1989).

101. *Blowhole ligament*.—Absent (0); present (1) (Heyning, 1989).

102. *Right posterior nasal sac* (ordered).—Present and elongate, in some cases it reaches osteological vertex (0); reduced and short, never reaches osteological vertex (1); absent (2). This is the same as the frontal sac of physeterids.

103. *Nasofrontal sacs* (ordered).—Absent (0); portions posterior to nasal passage present (1); same as previous state except that sacs extend anterodorsally around, and then in front of, the nasal passages; in dorsal view both nasofrontal sacs form a horseshoe shape (2) (Heyning, 1989).

104. *Inferior vestibule*.—Absent (0); present, forms a diverticulum of nasal passages posterior to blowhole ligament (1) (Heyning, 1989).

105. *Premaxillary sacs*.—Absent (0); present, form the most proximal diverticulum of nasal passages; they extend anteriorly adjacent to dorsal surfaces of premaxillae (1) (Heyning, 1989).

106. *Accessory sac*.—Absent (0); present, forms a small diverticulum of inferior vestibule and extends anterolaterally around the attachment of blowhole ligament to premaxilla (1) (Mead, 1975).

*Vertex and Area Adjacent to Nares*

107. *Inflection of ascending process of premaxilla*.—Gradual with premaxilla in dorsal view smoothly tapering as premaxilla shifts from a horizontal to a mostly vertical position (0); abrupt with an anterior splint of maxilla that emarginates the posterior edge of premaxilla and splits it into a posterolateral plate and posteromedial splint (1) . The inflection point is where the premaxilla shifts from a primarily horizontal position, mainly on the rostrum, to a more vertical position, mainly adjacent and posterior to the nasal openings (derived from Fordyce, 1994).

108. *Position of inflection of premaxilla* (ordered).—In transverse line with P1 (0); in line with P2 or about 18% of total rostral length towards anterior edge of rostrum (1); just anterior to or in line with anterior edge of supraorbital process of frontal (2); in line with anterior half of supraorbital process of frontal or in line with the halfway point, anteroposteriorly, of supraorbital process (3); in line with posterior half of supraorbital process or in line with postorbital process of frontal (4); in line with gap between postorbital process and the anterior tip of zygomatic process of squamosal or in line with the anterior tip of the latter process, space is absent in some taxa (5); in line with space between the anterior tip of zygomatic process of squamosal and anterior edge of the floor of squamosal fossa or in line with anterior edge of the floor of squamosal fossa (6); posterior to anterior edge of the floor of squamosal fossa (7).

109. *Premaxillary cleft*.—Absent (0); present, posterior part of ascending process of premaxilla bears a distinct cleft that originates from at posterior edge of the premaxilla and continues anteriorly dividing the premaxilla in two (1); present but cleft is shallow (2). In odontocetes, the cleft begins at the anterior splint of the maxilla and helps divide the posterolateral plate from the posteromedial splint.

110. *Premaxillae adjacent to and at posterior edge of the nasal opening* (ordered).—Do not clearly overhang maxillae (0); premaxillae overhang maxillae (1); premaxillae greatly enlarged laterally, region between lateral edge of the right premaxilla and supraoccipital is partially enclosed (2) (Muizon, 1991).

111. *Narial pit* (new term).—Absent (0); present, an anterior extension of nasal passage and forms a blind pocket in the maxilla just dorsal to maxilla/palatine suture; medial wall of pocket is formed by vomer (1). This character is usually not visible in complete skulls.

112. *Posterior end of ascending process of premaxillae* (ordered).—Face anterolaterally (0); face anteriorly (1); face anteromedially (2) (modified from Moore, 1968).

113. *Nasal bones* (ordered).—Two (0); one (1); none (2) (Heyning, 1989).

114. *Suture between right and left nasals and right and left frontals* (ordered).—Shifted towards right side (0); situated on midline (1); shifted towards left side (2) (Barnes, 1985). Character state “1” is probably the primitive condition for Cetacea and Artiodactyla; however, the character was coded with suture on the right side as state “0” in order to implement the ordering of states in the phylogenetic analyses.

115. *Dorsoventral thickness of anterior edge of nasal* (ordered).—Very thin, nasal thickness < 82% of the anterior nasal width (0); thick, nasal thickness between 100% and 173% of the nasal width (1); very thick, > 200% the nasal width (2). Measurement taken approximately five millimeters from anterior edge.

116. *Both nasals in dorsal view*.—Anterior edges are straight in one transverse plane (0); with point on midline and a gap on each side between premaxilla and nasal (1) (Moore, 1968). The shape of the nasals is based on the dorsal surface. In some cases the nasals may come to a point medially but at a more ventral position adjacent to the mesethmoid plate (state 0)(e.g. *Inia* AMNH 93412).

117. *Nasal* (ordered).—Elongate anteroposterior plate or blocky (0); anteroposteriorly compressed into a nearly vertical plate with fossa on ventrolateral surface for posterior nasal sac (caudal sac of Cranford et al., 1996) (1); fossa well excavated with boss delimiting dorsal edge (2) (modified from Muizon, 1988a; Messenger and McGuire, 1998).

118. *Nasals*.—Medial portions roughly in same horizontal plane as lateral portions or higher (0); medial portions greatly depressed forming a median trough immediately posterior to nasal openings (1) (Muizon, 1988a, 1991). This character appears in some ziphiids and also some kentriodontids where it is called the internasal fossa by Muizon (1988a).

119. *Maximum transverse width of both nasals* (ordered).—Very narrow, < 37% of the maximum width of the external bony nares (0); narrow, width between 55% and 89% of the nares width (1); within 10% of the nares width (2); wide, width between 123% and 140% the nares width (3); very wide, between 152% and 160% the nares width (4); extremely wide, width > 188% the width of external bony nares (5).

120. *Combined width of posterior edge of nasals* (ordered).—Wide, width > 150% the maximum width of external bony nares (0); subequal to external nares, width between 85% and 135% the nares width (1); narrow, width between 79% and 50% the nares width (2); very narrow, width between 44% and 39% the nares width (3); extremely narrow, width < 31% the nares width (4). The combined width can only be measured where the nasals meet on the midline. In some taxa there is a more posterior point where the width is wider, but the nasals, in such cases, are separated medially by the frontals.

121. *Nasal/Frontal suture*.—Approximately straight transversely (0); frontal has anterior wedge between posterior ends of nasals (1) (Muizon, 1988a).

122. *Position of posteriormost edge of nasals* (ordered).—Just anterior to or in a transverse line with anterior edge of supraorbital process of frontal (0); in line with anterior half of supraorbital process of frontal or in line with the halfway point, anteroposteriorly, of supraorbital process (1); in line with posterior half of supraorbital process or in line with postorbital process of frontal (2); in line with gap between postorbital process and the anterior tip of zygomatic process of squamosal or in line with the anterior tip of the latter process (3); between anterior the tip of zygomatic process of squamosal and anterior edge of the floor of squamosal fossa or in line with anterior edge of the floor of squamosal fossa (4); posterior to the anterior edge of the floor of the squamosal fossa (5). The length of the nasal is determined by the positions of its anterior and posterior edges; therefore, its length is not scored as a separate character.

123. *Height of posterior portions of nasals relative to lateral edge of maxilla* (ordered).—Approximately equal to height of the base of rostrum (see character 58 for a description of measurement used); nasal height between 92% and 139% the rostral height (0); elevated above rostrum, height of nasals between 156% and 203% the rostral height (1); very elevated, height of nasals between 229% and 282% the rostral height (2); extremely elevated, height of nasals between 354% and 420% the rostral height (3); nasals tower above facial part of skull, height of nasals > 548% the rostral height (4) (modified from Heyning, 1989).

124. *Frontals* (ordered).—Lower than nasals (0); same height as nasals (1); higher than nasals (2) (Muizon, 1988a). Messenger and McGuire (1998) coded the presence of the frontal protuberance as a separate character; however, taxa with a frontal protuberance always have the frontals higher than the nasals.

125. *Frontals posterior to nasals* *and between the premaxillae* (ordered).—Wider than maximum transverse width across nasals (0); same width as nasals (1); narrower than nasals, maxillae expanded medially posterior to the nasals (2) (Muizon, 1988a). Cannot be scored for taxa that lack maxillae posterior to the nasal bone.

126. *Dorsal exposure of frontals*.—Fairly flat with separation between right and left frontal obscure (0); frontals are nodular with distinct separating sulcus on midline (1) (Fordyce, 1994).

127. *Anterodorsal wall of braincase*.—Formed by frontal (0); mostly formed by maxilla (1) (Schulte, 1917; Miller, 1923).

128. *Supraoccipital* (ordered).—Below frontals and/or nasals, whichever is higher (0); at same level as frontals and/or nasals (1); higher than frontals and/or nasals (2) (modified from Moore, 1968).

129. *Maxilla on dorsal surface of skull*.—Does not contact supraoccipital posteriorly, maxilla separated by frontal and/or parietal (0); contact present (1) (Muizon, 1991, 1994).

*Temporal Fossae, Zygoma, Occiput*

130. *Temporal crest*.—Dorsal surface adjacent to crest is nearly horizontal, crest appears to be directed laterally (0); dorsal surface is concave and surface of temporal fossa below crest faces almost entirely laterally, crest appears to be oriented dorsolaterally (1) (Muizon, 1988a).

131. *Temporal crest* (ordered).—In posterior position, frontal roofs over the anterior third or more of temporal fossa (0); on posterior edge of supraorbital process of frontal (1); lateral end of temporal crest on dorsal surface of supraorbital process (2); entire crest on dorsal surface of supraorbital process (3) (modified from Heyning, 1989).

132. *Roof of temporal fossa*.—Frontal (0); frontal but with large opening through which maxilla and/or premaxilla is exposed, margins of the window are formed by a frontal ring (1). This can only be scored in taxa that have a posterior position of the temporal crest.

133. *Frontal/parietal suture in lateral view*.—Vertical or slightly angled posteroventrally (0); dorsal portion of suture pointed and extended far anterior so that the anteriormost point of parietal is anterior to the posteriormost point of premaxilla (1) (Miller, 1923).

134. *Parietals in dorsal view*.—Contact each other on the midline or are separated by an interparietal (0); are in place in the skull roof but are visible only as small triangular areas at edges of the intertemporal constriction, supraoccipital overlaps median portions of parietals and obscures them (1); are completely absent in the skull roof (2); visible only as triangular areas dorsolateral to supraoccipital, supraoccipital does not overlap parietals but separates and contacts them along an irregular suture (3) (Whitmore and Sanders, 1977; Barnes 1990).

135. *Interparietal*.—Present (0); absent or fused so that it is not distinguishable from parietals and frontals (1).

136. *Cross-section through intertemporal region, including parietals* (ordered).—Ovoid cross-section with sagittal crest (0); ovoid but sagittal crest absent (1); pinched ventrally and dorsal part expanded laterally, expanded part is rounded-over in cross-section (2); dorsal part is greatly expanded, overhangs more ventral portions, and lateral edge of dorsal surface is a sharp ridge (3).

137. *Length of intertemporal region, ventral view of skull roof with basicranium removed* (ordered).—Intertemporal region absent or short, canal in frontals that contained olfactory stem and bulbs is < 10% of the length from posterior edge of dorsal nasal sinuses to posterior edge of skull (0); long, canal is between 18% and 35% of that length (1); very long, canal > 44% of that length (2).

138. *Dorsoventral thickness of intertemporal region* (ordered).—Thin, thickness is < 25% the maximum height of skull, as measured from intercondylar notch to dorsal-most point of the supraoccipital (0); thick, thickness between 30% and 43% of the skull height (1); very thick, thickness > 54% of the skull height (2).

139. *Anteriormost point of the supraoccipital, in dorsal view* (ordered).—In transverse line with space between posterior edge of skull and anterior edge of the floor of squamosal fossa (0); in line with space between anterior edge of the floor of squamosal fossa and the anterior tip of zygomatic process of squamosal (1); in line with gap between anterior edge of zygomatic process of squamosal and the anteriormost point along posterior edge of the supraorbital process of frontal (2); in line with supraorbital process of frontal (3); in line with or anterior to anterior edge of supraorbital process of frontal; anterior edge of supraorbital process is taken at its medialmost point (4) (modified from Miller, 1923).

140. *Pronounced bulge anterior to alisphenoid exposure in temporal fossa*.—Absent (0); present (1).

141. *Alisphenoid*.—Broadly exposed laterally in temporal fossa (0); lateral surface is broadly overlapped by parietal so that only a narrow strip on the ventral edge of temporal fossa is visible in lateral view (1). This character is not applicable for taxa that lack a dorsally expanded alisphenoid.

142. *Zygomatic process of squamosal*.—Directed anteriorly (0); directed anterolaterally (1) (Sanders and Barnes, 2002).

143. *Dorsal edge of zygomatic process, skull in lateral view*.—Gently convex dorsally (0); near anterior end there is a distinct dorsal flange or process , flange usually articulates with frontal (1); concave dorsally (2).

144. *Emargination of posterior edge of zygomatic process by sternomastoid muscle fossa, skull in lateral view* (ordered).—Absent, posterior edge forms nearly a right angle with dorsal edge of zygomatic process of squamosal (0); slight emargination (1); deep emargination (2).

145. *Width of squamosal lateral to exoccipital, skull in posterior view* (ordered).— Narrow, exposed portion of squamosal < 14% the distance between sagittal plane and lateral edge of exoccipital (0); intermediate width, width between 16% and 35% of that distance (1); wide, width between 40% and 55% of that distance (2); very wide, width > 129% the distance between sagittal plane and lateral edge of exoccipital (3).

146. *Depth of squamosal fossa* (ordered).—Absent or very shallow, depth of fossa < 52% the horizontal distance from dorsal edge of zygoma to the point above deepest part of squamosal fossa (0); shallow, depth between 55% and 91% that distance (1); deep, depth between 98% and 168% that distance (2); very deep, depth greater than 180% that distance (3) (derived from Barnes, 1985). The depth of the fossa is measured relative to the dorsal edge of the zygomatic process, which forms the lateral border of the fossa.

147. *Longitudinal profile of floor of squamosal fossa*.—Highly sigmoidal, concave posteriorly in region of a secondary squamosal fossa (*sensu* Sanders and Barnes, 2002) but convex anteriorly (0); slightly sigmoidal, posterior part concave but does not form a discrete pit (1); flat (2); convex (3).

148. *Floor of squamosal fossa*.—Same dorsoventral thickness anteriorly and posteriorly (0); thickens posteriorly (1).

149. *Squamosal prominence*.—Absent (0); present, forms a medial projection on crest that forms the lateral edge of squamosal fossa, is continuous with a dorsoventral ridge on lateral wall of the squamosal fossa (1) (Sanders and Barnes, 2002).

150. *Ventral edge of zygomatic process of squamosal in lateral view* (ordered).—Concave ventrally (0); straight (1); convex ventrally (2). Geisler and Sanders (2003) treated this as an unordered character.

151. *Postglenoid process in lateral view* (ordered).—Tapers ventrally to a point (0); anterior and posterior sides nearly parallel with squared-off ventral end (1); same as state “1” except anteroposterior diameter of postglenoid process is very wide (2).

152. *Anterior edge of supraoccipital in dorsoposterior view* (ordered).—Triangular, pointed anteriorly (0); semicircular (1); rectangular (2) (Barnes, 1985).

153. *Lambdoidal crests of supraoccipital*.—Horizontal and directed laterally, overhanging temporal fossae (0); directed dorsolaterally, not or only slightly overhanging temporal fossae (1); very low and not directed either way (2).

154. *Posteromedial wall of temporal fossa*.—Visible in dorsal view (0); hidden in dorsal view by lateral edges of supraoccipital (1). Cannot be scored for taxa where the anteriormost point of supraoccipital is posterior to the level of the anterior edge of the floor for the squamosal fossa.

155. *Occipital shield*.—Smoothly convex or concave (0); bears distinct sagittal crest (1) (Sanders and Barnes, 2002).

156. *Dorsal condyloid fossa* (ordered).—Absent (0); present, situated anterodorsal to dorsal edge of condyle (1); present and forms a deep pit (2) (Sanders and Barnes, 2002).

*Anterior Basicranium*

157. *Anterior sinus* (ordered).—Absent (0); present but short (1); elongate with corresponding trough on maxilla (2) (Fraser and Purves, 1960). *Inia* has a very elongate anterior sinus; however, its corresponding trough on the maxilla is absent. All other taxa that have an elongate sinus also have a well-defined trough.

158. *Palatine*.—Relatively thin, floors posterior part of nasal cavity (0); thick, forms part of the anterior wall of the nasal cavities (1) (Miller, 1923).

159. *Palatines* (ordered).—Exposed ventrally (0); partially covered by pterygoid dividing it into medial and lateral exposures (1); ventral surfaces covered completely by pterygoids (2) (Miller, 1923; Muizon, 1987).

160. *Palatine* (ordered).—Ventral surface flat or convex (0); bears fossa for anterior end of pterygoid sinus (1); fossa well developed, divides palatine into medial and lateral laminae (2) (Muizon, 1988a, 1991).

161. *Lateral lamina of palatine*.—Free from or sutured to maxilla (0); fused to maxilla (1) (Muizon, 1988a).

162. *Pterygoid/palatine suture in ventral view* (ordered).—Angled anterolaterally (0); nearly transverse, pterygoid forms a substantial part of subtemporal crest (1); angled anteromedially (2).

163. *Pterygoid sinus fossa* (ordered).—Absent or cannot be distinguished from anterior part of fossa for cavum tympani (0); present, anterior edge approximately in line with anterior edge of foramen ovale (1); present and extended well anterior to foramen ovale (2); extended anterior to anterior edge of orbit (3) (modified from Fraser and Purves, 1960).

164. *Lateral lamina (outer plate or external duplication) of pterygoid*.—Present (0); partial, restricted to region lateral to the hamular process (1); absent (2) (Miller, 1923; Fraser and Purves 1960).

165. *Subtemporal crest*.—Present on alisphenoid and/or pterygoid, marks lateral edge of pterygoid fossa (0); subtemporal crest absent, pterygoid fossa extended laterally into orbital region (1) (derived from Fordyce, 1994).

166. *Inferior lamina of pterygoid*.—Absent or restricted to extreme anterior edge of pterygoid sinus cavity (0); present and floors most of sinus cavity (1) (modified from Fraser and Purves, 1960).

167. *Superior lamina of pterygoid* (ordered).—Present and covers most of ventral exposure of alisphenoid (0); absent from the sphenoidal region but present in orbital region (1); partially absent from orbital region (2); completely absent from orbital region (3) (Miller, 1923; Fraser and Purves, 1960).

168. *Posterior part of pterygoid sinus fossa, region immediately anterior to exit for mandibular branch of trigeminal nerve* (ordered).—One single fossa (0); split into a smaller and shallower posterior fossa and a much larger anteriorly extended deeper fossa by a low ridge (1); same as state “1” except divided by a high ridge (2).

169. *Preorbital lobe of pterygoid sinus* (ordered).—Absent (0); present but small (1); present and enlarged (2); enlarged and forms distinct excavation anterior to optic foramen (3); enlarged and extended posterodorsally over frontals to be roofed by maxilla (4) (Fraser and Purves, 1960).

170. *Postorbital lobe of pterygoid sinus* (ordered).—Absent (0); present but small (1); present and enlarged (2); enlarged and forms prominent fossa on ventral surface of supraorbital process of frontal posterior to optic foramen (3) (Fraser and Purves, 1960).

171. *Coalescence of pre-orbital and post-orbital lobes of pterygoid sinus dorsal to optic nerve*.—Absent (0); present (1) (Fraser and Purves, 1960).

172. *Hamular process of the pterygoid*.—Splint like (0); solid, long and subconical (1); hollow and excavated by pterygoid sinus, lateral side highly concave, may or may not have lateral and inferior laminae (2); form thin horizontal plates (3); absent (4) (modified from Fraser and Purves, 1960).

173. *Hamular processes of pterygoids*.—Rounded over in ventral view (0); bear anteroposterior keels (1) (Muizon, 1988a).

174. *Posteriormost point of* *hamular process of pterygoid, or medial part of pterygoid if the hamular process is absent* (ordered).—In transverse line with the middle of orbit (0); in line with postorbital process (1); in line with anterior edge of zygomatic process of squamosal (2); in line with middle of zygomatic process (3); in line with the postglenoid process (4).

*Posterior Basicranium*

175. *Fossa for pterygoid sinus on alisphenoid posterior to groove for mandibular branch of trigeminal nerve* (ordered).—Absent, bone is flat or not ossified because of enlarged internal foramen ovale (0); shallow fossa (1); deep subcircular fossa (2) (modified from Fordyce, 1994).

176. *Falciform process of squamosal*.—Plate-like with a wide, anteroposteriorly, base (0); rod-like with narrow base (1); poorly-developed or absent (2).

177. *Falciform process of the squamosal*.—Medial surface sutured to lateral lamina of pterygoid (0); not sutured (1). Cannot be scored for taxa that lack a lateral lamina of the pterygoid.

178. *Tympanosquamosal recess* (ordered).—Absent (0); absent but a small rectangular fossa for sigmoid process of the tympanic present, its long axis is transverse, and it is located medial to postglenoid process of squamosal (1); present and enlarged, forms a triangular fossa medial and anteromedial to postglenoid process (2); very large, forms large fossa that borders entire medial edge of glenoid fossa (3) (derived from Fraser and Purves, 1960).

179. *Lateral edge of middle sinus*.—Smooth (0); deckle-edged (1) (derived from Fraser and Purves, 1960).

180. *Position of alisphenoid/squamosal suture, skull in ventral view* (ordered).—Anterior to external foramen of foramen ovale or homologous groove (0); courses along groove for mandibular branch of trigeminal nerve, or just posterior to it (1); just medial to anterior edge of floor of squamosal fossa, foramen ovale, and/or groove situated entirely on alisphenoid (2).

181. *Groove for mandibular branch of trigeminal nerve*.—Directed laterally and is entirely posterior to pterygoid sinus fossa (0); lateral end of groove wraps laterally around the posterior end of the pterygoid sinus fossa and opens primarily anteriorly (1).

182. *Ventral part of squamosal posterior to postmeatic process* (ordered).—Large area of laminated bone, appears externally as multiple sutures (0); small area of laminated bone restricted to ventrolateral edge of squamosal (1); without laminated bone (2) (Kasuya, 1973).

183. *Cranial hiatus*.—Absent, petrosal contacts basioccipital medially or partially separated by narrow fissure (0); present but constricted, a medial projection of parietal partially divides fenestra (1); absent, parietal contacts basisphenoid and/or basioccipital dividing fenestra in two (2); present, wide space between basioccipital and both petrosal and squamosal (3). The cranial hiatus is formed by a coalescence of the internal foramen ovale and the basicapsular fissure (modified from Heyning , 1989a and Luo and Gingerich, 1999).

184. *Periotic fossa*.—Bowl-shaped (0); has transverse ridge that divides it into anterior and posterior portions (1). Cannot be scored for taxa that lack a periotic fossa.

185. *Suprameatal pit of squamosal* (ordered).—Absent (0); present but shallow, situated dorsolateral to spiny process of squamosal (1); forms a deep dorsolateral excavation into squamosal (2).

186. *Foramen spinosum*.—Absent (0); present, located in anteromedial corner of anterior part of periotic fossa near or on squamosal/parietal suture (1) (Muizon, 1994).

187. *Posterior portion of periotic fossa*.—Posteromedial part contains a deep, large fossa (0); fossa present but shallow (1); fossa is highly compressed and forms a narrow slit or a small blind foramen (2); fossa absent, posterior portion of the periotic fossa is of uniform depth (3).

188. *Zygomatic process of squamosal* (ordered).—Very short, length of process < 92% of the maximum width of glenoid fossa (0); short, length between 103% and 162% of glenoid width (1); intermediate length, length between 171% and 189% of the glenoid width (2); long, length between 198% and 271% of glenoid width (3); very long, length > 300% of the width of glenoid fossa (4). State “3” or state “4” is probably the primitive condition for Cetacea and Artiodactyla; however, the character was coded with state “0” (short zygomatic processes) in order to implement the ordering of states in the phylogenetic analyses.

189. *External auditory meatus*.—Wide (0); narrow (1) (Fordyce, 1994).

190. *Vomer*.—Posterior edge terminates on or at anterior edge of basisphenoid (0); terminates on basioccipital covering basioccipital/basisphenoid suture ventrally (1) (Barnes, 1984).

191. *Basioccipital crest*.—Narrow transversely (0); wide and bulbous (1) (Sanders and Barnes, 2002).

192. *Rectus capitus anticus muscle fossa*.—Absent or poorly developed (0); present with a well-defined anterior edge (1). The anterior edge forms a curved ridge that joins the basioccipital crest laterally and curves posteromedially to join its counterpart at the sagittal plane.

193. *Posteroventralmost point of basioccipital crest*.—Rounded over (0); forms a closely appressed separate flange, a narrow crease separates it dorsally from rest of basioccipital crest (1); distinct flange that projects posteriorly (2); distinct but separated by a pronounced notch that interrupts basioccipital crest (3).

194. *Angle formed by the basioccipital crests in ventral view* (ordered).—Parallel with no angle formed (0); 15° to 40° (1); 45° to 68° (2); 74° to 90° (3); > 100° (4) (modified from Muizon, 1991).

195. *Hypoglossal foramen*.—Thick bone separating it from jugular foramen, or jugular notch (0); separating bone very thin or absent, in the latter case hypoglossal foramen becomes confluent with jugular foramen (1).

196. *Jugular notch, gap between paroccipital process and basioccipital crest*. — Open notch, opening and depth of the notch are roughly equal (0); narrow and almost slit-like, depth is much greater than width of opening (1).

197. *Paroccipital process, skull in ventral view* (ordered).—Angled posterolaterally, extends posterior to posteriormost edge of condyle (0); posterior edge in transverse line with posterior edge of condyle (1); posterior edge is well anterior to posterior edge of condyle (2).

*Malleus*

198. *Tuberculum of the malleus*.—Unreduced (0); highly reduced, almost indistinguishable from articular head (1) (Doran, 1876).

199. *Processus muscularis of malleus* (ordered).—Processus muscularis shorter than manubrium of malleus (0); subequal (1); processes muscularis longer than manubrium (2) (modified from Muizon, 1988a).

*Petrosal (Periotic)*

200. *Apex of anterior process of the petrosal*.—In ventral or dorsal view blunt or pointed (0); bears tubercle (1) (Luo and Marsh, 1996).

201. *Anterior process in lateral view*.—Anterior edge of anterior process squared-off (0); comes to a blunt apex (1); comes to a slender point (2) (modified from Muizon, 1988a).

202. *Apex of anterior process of the petrosal*.—At same level or dorsal to ventral edge of pars cochlearis (0); well ventral to ventral edge of the pars cochlearis, process appears to be ventrally deflected (1) (modified from Fordyce, 1994).

203. *Length of anterior process of the petrosal* (ordered).—Absent (0); present but very short, length < 36% of the length of pars cochlearis (1); short, length between 59% and 94% of the promontorial length (2); nearly the same as length of pars cochlearis, length between 100% and 134% of the promontorial length (3); long, length between 141% and 174% of the promontorial length (4); very long, length > 212% of the length of the pars cochlearis (5) (modified from the following: Muizon, 1988; Luo and Marsh, 1996; Geisler and Luo, 1996). The length of the pars cochlearis was measured from its anterior edge to the ventral edge of the fenestra rotunda.

204. *Anterior process in lateral view*.—Ventral edge convex ventrally or nearly flat (0); ventral edge clearly concave (1) (Muizon, 1988a). The fovea epitubaria, which articulates with the accessory ossicle of the tympanic, is greatly expanded anteriorly in state “1”.

205. *Anteroexternal sulcus*.—Absent (0); present on lateral surface of anterior process of petrosal, oriented primarily anteroposteriorly but bowed ventrally (1) (modified from Fordyce, 1994).

206. *Sulcus for capsuloparietal emissary vein*.—Present, forms a dorsoventral groove on lateral side of anterior process immediately anterior to lateral tuberosity (0); absent (1) (derived from Geisler and Luo, 1998).

207. *Articulation of anterior process with squamosal* (ordered).—Extensive, most of lateral side contacts squamosal (0); large centrally-oriented ovoid region contacts squamosal, free around the edges (1); contact is very small (2); contact is absent, articulates via ligaments (3).

208. *Shape of cross section through anterior process at midlength* (ordered).—Highly elliptical, transverse diameter is < 36% the dorsoventral diameter (0); ovoid, transverse diameter is between 51% and 78% of the dorsoventral diameter (1); approximately circular, transverse diameter between 85% and 134% of the dorsoventral diameter (2); bulbous, transverse diameter > 141% of the dorsoventral diameter (3) (modified from Fordyce, 1994 and Luo and Marsh, 1996).

209. *Contact of anterior process of petrosal with portion of ectotympanic bulla anterior to accessory ossicle* (ordered).—Absent (0); present but no clear fossa for articulation on petrosal (1); anterior bullar facet present but shallow with poorly defined medial edge (2); present with well defined medial and lateral edges (3) (Fordyce, 1994). Cannot be scored for taxa in which the bulla is completely fused to the anterior process of the petrosal or in some taxa with very short anterior processes.

210. *Flange of anterior process of petrosal*.—Absent (0); present (1) (Geisler and Luo, 1996; Luo and Marsh, 1996). The flange is here redefined as part of the lateral tuberosity, specifically a horizontal shelf on its lateral and anterior sides that overhangs the anterior process.

211. *Lateral tuberosity* (ordered).—Absent (0); present, forms a bulbous prominence lateral to fossa for malleus (1); present and elongate, forms a lateral process that articulates dorsally with squamosal (2) (Muizon, 1991; Luo and Marsh, 1996; Geisler and Luo, 1996).

212. *Emargination of lateral edge of petrosal by hiatus epitympanicus*.—With petrosal in ventral view, emargination is narrow and is situated slightly posterior to base of posterior process (0); emargination is wide and is approximately in line with gap between fenestrae ovalis and rotunda (1).

213. *Fossa incudis* (ordered).—Poorly defined or cannot be differentiated from rest of epitympanic recess (0); forms a clear circular fossa (1); circular fossa present on a short pedestal, the incudal process (2) (Luo and Marsh, 1996).

214. *Fossa for malleus*.—Present (0); absent or poorly developed (1) (Geisler and Luo, 1996).

215. *Ventrolateral ridge of petrosal* (ordered).—Absent (0); present (1); present and expanded (2) (Geisler and Luo, 1996).

216. *Lateral side of petrosal* (ordered).—Entire side of petrosal contains pitted and rugose bone (0); all but anterior process is rugose (1); lateral side of posterior process of petrosal is pitted and rugose, remaining portions are smooth (2); entire side of petrosal is smooth (3).

217. *Origin of tensor tympani muscle* (ordered).—Deep, pocket-like fossa with anterior groove (0); anterior groove only (1); broad, poorly defined origin without a clear groove (2) (Luo and Marsh, 1996; Geisler and Luo, 1998).

218. *Angle between anterior process of petrosal and anterior edge of pars cochlearis* (ordered).—Obtuse, pars cochlearis appears transversely compressed (0); nearly 90 degrees, pars cochlearis looks rectangular or semicircular in ventral view (1); acute, pars cochlearis looks globular (2). Covers part of character 18 of Luo and Marsh (1996).

219. *Anteromedial corner of pars cochlearis*.—Rounded (0); angular (1) (Muizon, 1987; Fordyce, 1994).

220. *Pars cochlearis*.—Most convex part is on ventrolateral surface (0); most convex part is on medial surface. Area of greatest convexity begins anteromedial to fenestra rotunda and extends anterodorsally on the medial face. With petrosal in dorsal view, there is a wide expanse of bone medial to internal acoustic meatus (1).

221. *Ridge on anterolateral side of pars cochlearis, petrosal in ventral view* (ordered).— Present and high, forms an anteroposterior ridge that also forms the medial edge of a trough for tensor tympani muscle (0); present and low (1); absent (2) (Luo and Marsh, 1996).

222. *Fenestra rotunda*.—Oval (0); shaped like a teardrop with a fissure directed towards the perilymphatic foramen (1) (Fordyce, 1994).

223. *Posterodorsal edge of stapedial muscle fossa*.—Ventral to or in line with dorsal edge of fenestra rotunda (0); well dorsal to fenestra rotunda (1).

224. *Stylomastoid fossa* (ordered).—Absent (0); present, situated on posterior face of pars cochlearis posterodorsal to stapedial muscle fossa (1); enlarged dorsally and medially, covers much of posterior face of pars cochlearis (2); enlarged posterolaterally onto posterior process of the petrosal (3) (Geisler and Luo, 1996).

225. *Caudal tympanic process of the petrosal*.—Prominent, its ventral and posterior edges form a right angle in medial view (0); low, its ventral and posterior edges are joined by a smooth curve (1).

226. *Caudal tympanic process of petrosal in posteromedial view*.—Well separated from crista parotica, no division between stapedial muscle fossa and stylomastoid foramen (0); narrow separation or contact, clear separation of stapedial muscle fossa and stylomastoid foramen (1).

227. *Perilymphatic foramen* (ordered).—Smaller than endolymphatic foramen (0); approximately the same size (1); much larger with narrow posterior edge (2) (modified from Muizon, 1987 and Fordyce, 1994).

228. *Distance between perilymphatic foramen and fenestra rotunda* (ordered).—No distance, both apertures are confluent (0); narrow, distance < 89% of the distance between fenestra ovalis and fenestra rotunda (1); wide, distance between 96% and 122% of the space between fenestrae ovalis and rotunda (2); very wide, distance > 146% (3) (modified from Geisler and Luo, 1996).

229. *Distance between endolymphatic foramen and fenestra rotunda* (ordered).—Very narrow, distance < 112% of the distance between fenestra ovalis and fenestra rotunda (0); narrow, distance between 121% and 185% (1); wide, distance between 192% and 211% (2); very wide, distance > 222% the distance between fenestra ovalis and fenestra rotunda (3).

230. *Elongation of pars cochlearis towards cranial cavity, dorsally and medially*.—Absent (0); present, inner porous bone expanded towards cranial cavity (1); present, outer periosteal bone of pars cochlearis expanded towards cranial cavity (2) (Geisler and Luo, 1996).

231. *Excavation of tegmen tympani at base of anterior process*.—Absent (0); present, fossa on dorsolateral side of tegmen tympani (1).

232. *Dorsal edge of tegmen tympani dorsolateral to internal acoustic meatus and anterior process* (ordered).—Present and high, dorsoventral height > 114% the width of pars cochlearis (0); present, height between 58% and 34% of promontorial width (1); low, height between 23% and 11% (2); forms a low ridge or is absent, height < 4% of the width of pars cochlearis (3) (derived from Fordyce, 1994).

233. *Dorsal edge of tegmen tympani lateral to endolymphatic foramen* (ordered).—Present and very high, dorsoventral height > 112% of the width of pars cochlearis (0); high, height between 95% and 50% the promontorial width (1); low, height between 12% and 4% (2); faint ridge (3); absent (4).

234. *Fundus of internal acoustic meatus*.—Funnel-like, smaller at the blind end and wider near the rim (0); tubular (1) (Luo and Marsh, 1996).

235. *Lateral wall of internal acoustic meatus*.—Low, does not protrude noticeably from suprameatal fossa and surrounding bone (0); high, a wedge-shaped area of elevated bone occurs between dorsal edge of tegmen tympani and internal acoustic meatus, extending the latter ventrally and increasing its depth (1).

236. *Foramen singulare* (ordered).—In common recess with the tractus spiralis foraminosus, transverse septum separating the foramen singulare from endocranial aperture of facial nerve is well developed (0); in common recess with tractus spiralis foraminosus, transverse septum separating it from facial nerve foramen is low, and endocranial aperture of facial nerve canal within internal acoustic meatus (1); separated by partitions of equal height from tractus spiralis foraminosus and endocranial aperture of facial nerve canal (2); in common recess with endocranial aperture of facial nerve canal (3) (modified from Luo and Marsh, 1996; Geisler and Luo, 1996).

237. *Endocranial aperture of facial nerve canal* (ordered).—Anterior to tractus spiralis foraminosus (0); slightly anterior, posterior edge of aperture of facial nerve canal is lateral to center of tractus spiralis foraminosus (1); lateral to tractus spiralis foraminosus (2).

238. *Morphology of endocranial opening of facial nerve canal*.—Continuous with an anterior fissure (0); oval-shaped (1); circular (2) (Luo and Marsh, 1996).

239. *Articular rim* (ordered).—Absent (0); present but small, forms a ridge anterolateral to articulation surface of the posterior process of the petrosal and separated from it by a sulcus; the ridge fits into a corresponding cavity posterolateral and slightly dorsal to spiny process of squamosal (1); present, long, oriented posterodorsally, and posterior end intersects dorsal margin of petrosal; in lateral view has sigmoidal shape (2); present with sigmoidal shape and laterally elongate with hook-like process (3) (modified from Muizon, 1987).

240. *Contact of petrosal, not including anterior process, with skull* (ordered).—Distal end of posterior process of petrosal, lateral surface of posterior process of the petrosal, and entire dorsal edge of tegmen tympani (or homologous bone) contact squamosal and possibly parietal (0); same as state “0” except dorsal edge contacts from posterior end of posterior process of petrosal to region just lateral to endolymphatic foramen (1); only dorsal and lateral sides of posterior process articulate with squamosal (2); petrosal articulates with squamosal along hiatus epitympanicus and adjacent regions on the posterior process (3); petrosal only articulates with skull via ligaments (4).

241. *Articulation surfaces on posterior processes of ectotympanic and petrosal* (ordered).—Surfaces smooth (0); bear complimentary longitudinal grooves and ridges (1); fused in adults (2) (Kasuya, 1973; Geisler and Luo, 1996).

242. *Ventral surface of posterior process of petrosal, along a straight path perpendicular to its long axis* (ordered).—Concave (0); flat (1); convex (2).

243. *Bullar facet on posterior process of petrosal*.—Restricted to ventral surface (0); extends dorsally onto posteromedial face of posterior process (1) (Fordyce, 1994).

244. *Facial nerve sulcus*.—Long sulcus on posterior process of petrosal or the compound petrosal/tympanic posterior process of most mysticetes (0); short, no sulcus posterior to stylomastoid notch (1) (Luo and Marsh, 1996; Geisler and Luo, 1996).

245. *Length of posterior process of petrosal* (ordered).—Absent or very short, length < 47% the length of pars cochlearis (0); short, length between 88% and 119% of the promontorial length (1); slightly longer than pars cochlearis, length between 131% and 153% (2); long, length between 191% and 404% (3); very long, length > 613% of the length of pars cochlearis (4) (modified from the following: Kasuya, 1973 ; Barnes, 1990; Luo and Marsh, 1996). The length of the pars cochlearis is measured as discussed in character 194. The length of the posterior process is measured along its long axis which is usually directed posterolaterally.

246. *Orientation of posterior process of petrosal* (ordered).—Forms an angle < 130° with the long axis of tegmen tympani (0); forms an angle between 135° and 165° (1); directed nearly posteriorly, forms an 180° with tegmen tympani (2) (Kasuya, 1973; Geisler and Luo, 1996).

247. *Dorsal edge of posterior process, petrosal in lateral or medial view*.—Straight or convex ventrally (0); concave ventrally, helps to form the neck of posterior process of petrosal (1) (modified from Geisler and Luo, 1996).

248. *Posterior process of petrosal*.—Robust (0); horizontal plate and very thin for most of its length (1) (Luo and Marsh, 1996).

249. *Mastoid exposure of posterior process of petrosal on the outside of skull*.—Exposed externally (0); not exposed, enclosed by the exoccipital and squamosal (1) (Luo and Marsh, 1996; Geisler and Luo, 1996).

*Ectotympanic*

250. *Anterior spine, or conical anterior tip, of ectotympanic bulla* (ordered).—Absent (0); present but small (1); present and long (2) (Kasuya, 1973; Muizon, 1987, 1994).

251. *Shape of ectotympanic bulla*.—Narrow and long, width of bulla at sigmoid process is < 64% the length of bulla along its long axis (0); wide, width of bulla > 65% of its long axis (1) (Kasuya, 1973). Following Kasuya (1973) this is determined by comparing the width of the bulla at the sigmoid process to the length of the bulla, measured from anterior end to posterior edge of involucrum.

252. *Posterior end of ventromedial keel*.—Forms a smooth curve around posterior part of involucrum (0); protrudes and points medially (1).

253. *Accessory ossicle*.—Absent (0); present (1) (Luo and Marsh, 1996; Luo, 1998).

254. *Accessory ossicle*.—Small and oblong (0); large and subspherical (1) (Fordyce, 1994; Luo and Marsh, 1996). Cannot be scored for taxa that lack an accessory ossicle.

255. *Accessory ossicle or homologous region on lip of bulla*.—Fused to anterior process of petrosal (0); not fused (1) (Barnes, 1990; Fordyce, 1994; Luo and Marsh, 1996).

256. *Lateral furrow of ectotympanic bulla*.—Present (0); absent (1) (Kasuya, 1973; Messenger and McGuire, 1998).

257. *Lateral furrow*.—Broad sulcus (0); narrow crease (1). Cannot be scored for taxa that lack a lateral furrow.

258. *Sigmoid process*.—Forms a straight transverse plate that is directed perpendicular to long axis of bulla (0); forms a curved plate; proximal part is directed posterolaterally while the distal end curves to point laterally (1) (Kasuya, 1973).

259. *Ventral margin of sigmoid process*.—Present (0); absent; in its place the lateral margin of sigmoid process smoothly turns into a sulcus on lateral side of bulla (1).

260. *Dorsal edge of sigmoid process*.—Contacts sigmoid fossa of squamosal (0); distal end expanded anteriorly to articulate with lateral tuberosity of petrosal only (1); does not articulate with squamosal or petrosal (2) (modified from Luo and Marsh, 1996). This character is related to character eight of Muizon (1988a).

261. *Elliptical foramen of ectotympanic bulla*.—Present, connection between ectotympanic bulla and its posterior process is split into two pedicles (0); absent (1) (Kasuya, 1973).

262. *Anterior edge of posterior process of ectotympanic bulla*.—Contacts postmeatic process of squamosal (0); contact absence (1) (Kasuya, 1973).

263. *Posterior process of tympanic*.—Contains sporadic areas of laminated bone at posterodorsal end (0); almost entire process is laminated bone (1) (Kasuya, 1973).

264. *Thickness of posterior process of ectotympanic*.—Thick in region ventral to articulating surface with petrosal (0); forms a thin lamina (1).

265. *Distal end of posterior process of ectotympanic* (ordered).—Thinner or approximately the same thickness as more proximal portions (0); distal end thicker but not hypertrophied (1); hypertrophied in size, forms large nodular mass (2) (Flower, 1872; Kasuya, 1973).

266. *Median furrow of ectotympanic bulla* (ordered).—Absent (0); forms notch on posterior edge of bulla between medial and lateral prominences (1); forms continuous anteroposterior groove on ventral surface of bulla (2) (Kasuya, 1973).

267. *Median furrow*.—On posterior side of bulla, it is divided by a transverse ridge originating from the involucrum (0); transverse ridge is absent (1).

268. *Profile of ectotympanic bulla in lateral view*.—Ventral edge convex or flat (0); concave because of posteroventral expansion of lateral prominence (1).

269. *Medial prominence of involucrum*.—Posterior edge approximately in line with posterior edge of lateral prominence (0); posterior edge distinctly anterior to posterior edge of lateral prominence (1) (Kasuya, 1973; Muizon, 1987). Degree of posterior extension is determined relative to the long axis of the bulla, not the orientation of the bulla in the skull. This alleviates problems associated with the reorientation of the bulla in the skull.

270. *Involucrum*.—In medial view, dorsal and ventral borders converge anteriorly (0); excavated anterior to base of posterior process so that dorsal and ventral sides are parallel (1) (Fordyce, 1994).

271. *Involucrum*.—Bears prominent transverse groove on dorsal surface that divides involucrum into a thicker posterior part and thinner anterior part (0); groove absent (1).

272. *Ridge on inside of bulla*.—Present, transverse ridge extends laterally from involucrum and partially divides cavum tympani into anterior and posterior portions (0); absent (1).

273. *Ventromedial keel of the ectotympanic bulla*.—Present along entire length (0); terminates approximately at level of lateral furrow (1); poorly defined along entire length (2) (Kasuya, 1973).

274. *Shape of ventromedial keel, bulla in dorsomedial view*.—Nearly straight (0); bowed medially (1).

275. *Region on dorsomedial side of ventromedial keel*.—Flat or convex (0); gently concave (1).

*Vertebral*

276. *Length of prelumbar vertebral column* (ordered).—Very short, length of cervical plus thoracic portions of vertebral column from 77% to 55% condylobasal length of skull (0); short, length of cervical plus thoracic portions of vertebral column from 100 to 83% skull length (1); long, from 123% to 114% skull length (2); very long, > 135% skull length (3) (derived from Miller, 1923).

277. *Atlas* (ordered).—Ventral process larger than dorsal process (0); both processes are subequal (1); dorsal process larger (2) (modified from Muizon, 1987,1988a).

278. *Atlas and axis vertebrae*.—Unfused (0); fused together (1).

279. *Cervical vertebrae posterior to atlas* (ordered).—All are separate (0); only 2nd and 3rd are fused together (1); 2nd through 4th are fused together (2); 2nd through 5th are fused (3); 2nd through 6th are fused (4); 2nd through 7th are fused (5) (modified from Miller, 1923). Fusion of the cervical vertebrae is related to extreme shortening of the neck.

280. *Number of thoracic vertebrae* (ordered).—18 to 17 (0); 16 to 15 (1); 14 (2); 13 (3); 12 (4); 11 (5); 10 or less (6).

281. *Number of thoracic vertebrae with capitular articulations* (ordered).—11 (0); 10 (1); 9 (2); 8 (3); 7 (4); 6 (5); 4 to 5 (6); 3 or less (7) (Sanders and Barnes, 2002).

282. *Capitular articulation facets of the posterior vertebrae*.—Facets gradually shift downward on sequential vertebrae to fuse with the tubercular facets (0); facets abruptly shift from a position on the neural arch to a pedestal that originates from the centrum on the subsequent vertebra (1) (Flower, 1869; Miller, 1923). Coding modified based on observations in Heyning (1989a).

283. *Lateral edge of transverse processes of lumbar vertebrae*.—Oriented anteroposteriorly (0); angled anteromedially 45° or more, relative to a parasagittal plane (1) (Muizon, 1988a).

284. *Transverse processes of lumbar vertebrae*.—Oriented ventrolaterally (0); oriented laterally and horizontally (1) (Sanders and Barnes, 2002).

285. *Lumbar vertebrae*.—Transverse processes narrow distally or are approximately the same anteroposterior width as their bases (0); transverse processes bear greatly expanded distal ends (1) (Muizon, 1988a).

286. *Centrum of anterior lumbar vertebrae* (ordered).—Short, length < 63% the width (0); long, length between 79% and 136% the width (1); very long, length > 147% the width (2) (Muizon, 1988a; Barnes, 1990). The width is measured across the anterior face of the centrum.

287. *Number of lumbar vertebrae* (ordered).—3 or less (0); 4 (1); 6 (2); 7 to 8 (3); 9 to 10 (4); 11 to 12 (5); 13 to 16 (6); 16 to 19 (7). Geisler and Sanders (2003) had 8 states for this character but no taxon had their state 0. This state is combined here with their state 1 yielding a total of 8 distinct character states.

288. *Number of caudal vertebrae* (ordered).— 13 to 15 (0); 16 to 19 (1); 20 to 23 (2); 24 to 27 (3); 27 to 30 (4); 30 to 33 (5); 34 to 60 (6).

*Forelimb and Pectoral Girdle*

289. *Ventrolateral processes on manubrium of sternum*.—Absent (0); present but small, occurs ventral to articulation surface for first costal cartilage or rib (1) (Muizon, 1988).

290. *Sternum*.—Comprised of several bones (0); comprised of one bone (1) (Yablokov, 1964).

291. *Sternum*.—Several ribs attach to sternum (0); one rib attaches to sternum (1) (Messenger and McGuire, 1998).

292. *Coracoid process of scapula*.—Present (0); absent or barely distinguishable from edge of glenoid fossa (1) (Muizon, 1987, 1994).

293. *Supraspinous fossa of scapula*.—Present (0); absent or nearly absent, acromion process on anterior edge of scapula (1) (Muizon, 1987, 1994).

294. *Prominent deltoid crest on anterior edge of humerus*.—Present, forms greatest anteroposterior diameter along shaft (0); forms a knob-like tuberosity (1); tuberosity and crest absent (2) (Sanders and Barnes, 2002).

295. *Delto-pectoral tuberosity or farthest anterior point of crest* (ordered).—Closer to proximal head of humerus (0); approximately centered, proximodistally, on shaft (1); closer to distal end of humerus (2) (Muizon, 1988a).

296. *Radial and ulnar facets of humerus*.—Forms one articulation surface that is semicircular in lateral view (0); two distinct facets that in lateral view form an obtuse angle (1) (Barnes, 1990).

297. *Humerus* (ordered).—Longer than radius and ulna (0); approximately the same length as radius and ulna (1); shorter than radius and ulna (2) (Sanders and Barnes, 2002).

298. *Olecranon process* (ordered).—Present as a distinct process (0); present as slightly raised proximal posterior edge (1); absent (2) (Barnes, 1990; Messenger and McGuire, 1998).

299. *Manus*.—Pentadactyl (0); tetradactyl (1) (Yablokov, 1964).

*Other*

300. *Esophageal forestomach*.—Present, epithelium is either completely devoid of glands or may contain a small patch (0); absent, first chamber of stomach has glandular epithelium (1) (derived from Mead, 1989a; Rice and Wolman, 1990).

301. *External throat grooves* (ordered).—Absent (0); one pair, one on each side of midline (1); one to five pairs of grooves (2); more than five pairs (3).

302. *Throat grooves*.—Converge anteriorly (0); parallel (1) (Flower, 1872).

303. *Sexual dimorphism* (ordered).—Males 45% larger than females (0); males 14% to 30% larger than females (1); females and males approximately the same size (2); females 5% to 10% larger than males (3); females 20% to 30% larger than males (4) (modified from Yablokov, 1964). Size is based on maximum anteroposterior length of the entire body. Although state 1 or 2 is probably the primitive state for Cetacea, the assignment of states used here simplifies the inclusion of the between state homology assumptions (i.e. ordering the states) in the phylogenetic analyses.

304. *Dorsal Fin* (ordered).—Absent (0); dorsal hump (1); present (2) (Messenger and McGuire, 1998).

REFERENCES

Allen GM: **A new fossil cetacean.** *Harv Univ Bull Mus Comp Zool* 1921, **65**:3-15.

Andrews RC: **The California gray whale (*Rhachianectes glaucas* Cope).** *Mem Am Mus Nat Hist* 1914, **1**:227-287.

Arnold PW, Heinsohn GE: **Phylogenetic status of the Irrawaddy dolphin *Orcaella brevirostris* (Owen in Gray): a cladistic analysis.** *Mem. Queensl. Mus. 1996*, **39**:141-204.

Baker AN: **Pygmy right whale *Caperea marginata* (Gray, 1846).** In *Handbook of Marine Mammals, Volume 3*. Edited by Ridgway SH, Harrison, R. New York: Academic Press; 1985:345-354.

Balcomb KC III: **Baird’s beaked whale *Berardius bairdii* Stejneger, 1883: Arnoux’s beaked whale *Berardius arnuxii* Duvernoy, 1851**. In *Handbook of Marine Mammals, Volume 4*. Edited by Ridgway SH, Harrison, R. New York: Academic Press; 1989:261-288.

Barnes LG: **Whales, dolphins and porpoises: origin and evolution of the Cetacea.** In *Mammals. Notes for a Short Course Organized by P. D. Gingerich and C. E. Badgely*. Edited by Broadhead TW. *Studies in Geology* 1984, **8** (1-4). University of Tennessee Department of Geological Sciences, Knoxville.

Barnes LG: **Fossil pontoporiid dolphins (Mammalia: Cetacea) from the Pacific coast of North America.** *Nat Hist Mus Los Angel County Contrib Sci* 1985, **363**: 1-34.

Barnes, LG: **The fossil record and evolutionary relationships of the genus *Tursiops*.** In *The Bottlenosed Dolphin*. Edited by Leatherwood S, Reeves RR. New York: Academic Press; 1990:3-26.

Barnes LG, Sanders, AE: The transition from archaeocetes to mysticetes: late Oligocene toothed mysticetes from near Charleston, South Carolina. In: *Sixth North American paleontological convention abstracts of papers*. Edited by Repetski JE. Paleontological Society Special Publication No. 8, 1996:24.

Barnes LG, Kimura M, Furusawa H, Sawamura H: **Classification and distribution of Oligocene Aetiocetidae (Mammalia; Cetacea; Mysticeti) from western North America and Japan**. *The Island Arc* 1994, **3**:392-431.

Best RC, da Silva VMF: **Amazon River Dolphin, Boto *Inia geoffrensis* (de Blainville, 1817)**. In *Handbook of Marine Mammals, Volume 4*. Edited by Ridgway SH, Harrison R. New York: Academic Press; 1989:1-23.

Brodie PF: **The white whale *Delphinapterus leucas* (Pallas, 1776)**. In *Handbook of Marine Mammals, Volume 4*. Edited by Ridgway SH, Harrison R. New York: Academic Press; 1989:119-144.

Brownell RL Jr: **Franciscana *Pontoporia blainvillei* (Gervais and d’Orbigny, 1844)**. In *Handbook of Marine Mammals, Volume 4*. Edited by Ridgway SH, Harrison R. New York: Academic Press; 1989:45-67.

Caldwell DK, Caldwell MC: **Pygmy sperm whale *Kogia breviceps* (de Blainville, 1838): dwarf sperm whale *Kogia simus* Owen, 1866**. In *Handbook of Marine Mammals, Volume 4*. Edited by Ridgway SH, Harrison R. New York: Academic Press; 1989:235-260.

Cranford TW, Amundin M, Norris KS: **Functional morphology and homology in the odontocete nasal complex: implications for sound generation.** *J Morphol* 1996, **228**:223-285.

Cummings WC: **Right whales *Eubalaena glacialis* (Müller, 1776) and *Eubalaena australis* (Desmoulins, 1822)**. In *Handbook of Marine Mammals, Volume 3*. Edited by Ridgway SH, Harrison, R. New York: Academic Press; 1985:275-304.

Doran AHG: **Morphology of the mammalian ossicula auditûs.** *Linn Soc Lond Trans 2nd Ser Zool* 1876, **1**:371-497.

Dubrovo IA, Sanders AE: **A new species of *Patriocetus* (Mammalia, Cetacea) from the Late Oligocene of Kazakhstan**. *J Vertebr Paleontol* 2000, **20**:577-590.

Emlong, D: **A new archaic cetacean from the Oligocene of Northwest Oregon**. *Univ Oregon Bull Mus Nat Hist* 1966, **3**: 1-51.

Fitzgerald EMG: **A bizarre new toothed mysticete (Cetacea) from Australia and the early evolution of baleen whales.** *Proc R Soc B* 2006, **273:**2955-2963.

Flower WH: **Description of the skeleton of *Inia geoffrensis* and of the skull of *Pontoporia blainvillii* with remarks on the systematic position of these animals in the Order Cetacea**. *Trans Zool Soc Lond* 1867, **6**:87-116.

Flower WH: **On the osteology of the cachalot or sperm-whale (*Physeter macrocephalus*).** *Trans Zool Soc Lond* 1869, **6**:309-372.

Flower WH: **On the recent ziphioid whales, with a description of the skeleton of *Berardius arnouxi***. *Trans Zool Soc Lond* 1872, **8**:203-234.

Flynn TT: **Description of *Prosqualodon davidi* Flynn, a fossil cetacean from Tasmania**. *Trans Zool Soc Lond* 1948, **26**:153-197.

Fordyce RE: **Systematics of the odontocete whale *Agorophius pygmaeus* and the family Agorophiidae (Mammalia, Cetacea)**. *J Paleontol* 1981, **55**:1028-1045.

Fordyce RE: ***Waipatia maerewhenua*, new genus and new species (Waipatiidae, new family), an archaic late Oligocene dolphin (Cetacea: Odontoceti: Platanistoidea) from New Zealand.** In *Contributions in Marine Mammal Paleontology Honoring Frank Whitmore Jr*. Edited by Berta A, Deméré TA. Proc. San Diego Soc. Nat. Hist., 1994, 29:147-176.

Fordyce RE: ***Simocetus rayi* (Odontoceti: Simocetidae, New Family): a bizarre new archaic Oligocene dolphin from the Eastern Pacific**. In *Cenozoic mammals of land and sea, tributes to the career of Clayton E. Ray*. Edited by Emry RE. *Smithson. Contrib. Paleobiology* 2002, **93**:185-222.

Fraser FC, Purves PE: **Hearing in cetaceans— evolution of the accessory air sacs and the structure and function of the outer and middle ear in recent cetaceans.** *Bull Br Mus (Nat Hist) Zool* 1960, **7**:1-140.

Gambell R: **Fin whale *Balaenoptera physalus* (Linnaeus, 1758)**. In *Handbook of Marine Mammals, Volume 3*. Edited by Ridgway SH, Harrison, R. New York: Academic Press; 1985:171-192.

Geisler JH, Luo Z-X: **The petrosal and inner ear of *Herpetocetus* sp. (Mammalia: Cetacea) and their implications for the phylogeny and hearing of archaic mysticetes**. *J Paleontol* 1996, **70**:1045-1066.

Geisler JH, Sanders AE: **Morphological Evidence for the Phylogeny of Cetacea.** *J Mamm Evol* 2003*,* **10**:23-129.

Gregory WK, Kellogg R: A fossil porpoise from California. *Amer. Mus. Novitates* 1927, **269**:1-7.

Heyning JE: **Comparative facial anatomy of beaked whales (Ziphiidae) and a systematic revision among the families of extant Odontoceti.** *Nat Hist Mus Los Angle Cty Contrib Sci* 1989a, **405**:1-64.

Heyning JE: **Cuvier’s beaked whale *Ziphius cavirostris* G. Cuvier**. In *Handbook of Marine Mammals, Volume 4*. Edited by Ridgway SH, Harrison R. New York: Academic Press; 1989b:289-308.

Heyning JE, Mead JG: **Evolution of the nasal anatomy of cetaceans**. In *Sensory Abilities of Cetaceans*. Edited by Thomas J, Kastelein R. New York: Plenum Press; 1990:67-79.

Hulbert RC Jr: **Postcranial osteology of the North American Middle Eocene protocetid *Georgiacetus***. In *The Emergence of Whales*. Edited by Thewissen, JGM. New York: Plenum Press; 1998:235-267.

Hulbert RC Jr, Petkewich RM, Bishop GA, Burky D, Aleshire DP: **A new middle Eocene protocetid whale (Mammalia: Cetacea: Archaeoceti) and associated biota from Georgia**. *J Paleontol* 1998, **72**:907-927.

Kasuya T: **Systematic consideration of recent toothed whales based on the morphology of tympano-periotic bone**. *Sci Rep Whales Res Inst* 1973, **25**: 1-103.

Kellogg AR: **Description of an apparently new toothed cetacean from South Carolina**. *Smithson Misc Coll* 1923a, **76**:1-7.

Kellogg AR: **Description of two squalodonts recently discovered in the Calvert Cliffs, Maryland; and notes on the shark-toothed cetaceans**. *Proc US Natl Mus* 1923b, **62**:1-69.

Kellogg AR: **On the occurrence of remains of fossil porpoises of the genus *Eurhinodelphis* in North America**. *Proc US Natl Mus* 1925, **66**:1-40.

Kellogg AR: **Supplementary observations on the skull of the fossil porpoise *Zarhachis flagellator* Cope**. *Proc US Natl Mus* 1926, **67**:1-18.

Kellogg AR: ***Kentriodon pernix,* a Miocene porpoise from Maryland**. *Proc US Natl Mus* 1927, **69**:1-55.

Kellogg AR: **A review of the Archaeoceti**. Carnegie Institution of Washington Publication 1936, **482**:1-366.

Kellogg R: **A new whalebone whale from the Miocene Calvert Formation**. *Bull US Natl Mus* 1965, **247**:1-45.

Kellogg R: **A hitherto unrecognised Calvert cetothere**. *Bull US Natl Mus* 1968, **247**:133-161.

Luo Z-X. **Homology and transformation of cetacean ectotympanic structures**. In *The Emergence of Whales*. Edited by Thewissen, JGM. New York: Plenum Press; 1998:269-301.

Luo Z-X, Gingerich PD: **Terrestrial Mesonychia to aquatic Cetacea: transformation of the basicranium and evolution of hearing in whales**. *University of Michigan Papers on Paleontology* 1999, **31**: 1-98.

Luo, Z-X, Marsh K: **Petrosal (periotic) and inner ear of a Pliocene kogiine whale (Kogiinae, Odontoceti): implications on relationships and hearing evolution of toothed whales**. *J Vertebr Paleontol* 1996, **16**: 328-348.

McLeod SA, Whitmore FC Jr, Barnes LG: **Evolutionary relationships and classification.** In *The Bowhead Whale*. Edited by Burns JJ, Montague, JJ, Cowles CJ. Society for Marine Mammalogy Special Publication No. 2, 1993:45-70.

Messenger SL, McGuire JA: **Morphology, molecules, and the phylogenetics of cetaceans.** *Syst Biol* 1998, **47**:90-124.

Mead JG: **Anatomy of the external nasal passage and facial complex in the Delphinidae (Mammalia: Cetacea)**. *Smithson Contrib Zool* 1975, **207**: 1-72.

Mead JG: **Shepherd’s beaked whale *Tasmacetus shepherdi* Oliver, 1937**. In *Handbook of Marine Mammals, Volume 4*. Edited by Ridgway SH, Harrison, R. New York: Academic Press; 1989:309-320.

Miller GS Jr: **A new river-dolphin from China**. *Smithson Misc Coll* 1918, **68**: 1-12.

Miller GS Jr: **The telescoping of the cetacean skull.** *Smithson Misc Coll* 1923, **75**:1-55.

Moore JC: **Relationships among the living genera of beaked whales.** *Fieldiana Zool* 1968, **53**: 209-298.

Muizon C de. **The affinities of *Notocetus vanbenedeni*, an early Miocene platanistoid (Cetacea, Mammalia) from Patagonia, southern Argentina**. *Am Mus Novit* 1987, **2904**: 1-27.

Muizon, C de. **Les vertébrés fossiles de la Formation Pisco (Pérou). deuxiéme partie: les Odontocétes (Cetacea, Mammalia) du Pliocéne inférieur de Sud-Sacaco**. *Trav Inst Fr Et Andines* 1984, **27**: 1-188.

Muizon C de: **Les relations phylogénétiques des Delphinida (Cetacea, Mammalia).** *Ann Paléontol* 1988a, **74**:159-227.

Muizon C de: **Les vertébrés fossiles de la Formation Pisco (Pérou) III: les odontocétes (Cetacea, Mammalia) du Miocéne**. *Rech sur Civ, mem* 1988b, **78**: 1-244.

Muizon C de: **A new Ziphiidae (Cetacea) from the early Miocene of Washington State (USA) and phylogenetic analysis of the major groups of odontocetes.** *Bull Mus Natn Hist Nat, Section C, 4ème série* 1991, **12**: 279-326.

Omura, H., Nishiwaki, M., Ichihara, T., and Kasuya, T. (1962). Osteological note of a sperm whale. *Sci Rep Whales Res Inst* **16**: 35-45.

Omura H, Ohsumi S, Nemoto T, Nasu K, Kasuya T: **Black right whales in the North Pacific**. *Sci Rep Whales Res Inst* 1969, **21**:1-78.

Peixun C: ***Lipotes vexillifer* Miller, 1918**. In *Handbook of Marine Mammals, Volume 4*. Edited by Ridgway SH, Harrison, R. New York: Academic Press; 1989:25-43.

Pilleri G, Gihr M: **The function and osteology of the manus of *Platanista gangetica* and *Platanista indi****.* *Invest. Cetacea* 1976, **7**: 109-118.

Reeves RR, Brownell RL Jr: ***Platanista gangetica* (Roxburgh, 1801) and *Platanista minor* Owev, 1853**. In *Handbook of Marine Mammals, Volume 4*. Edited by Ridgway SH, Harrison, R. New York: Academic Press; 1989:69-99

Rice DW, Wolman AW: **The stomach of *Kogia breviceps***. *J Mamm* 1990, **71**: 242-246.

Rommel, S. **Osteology of the bottlenose dolphin**. In *The bottlenose dolphin*. Edited by Leatherwood S, Reeves RR. San Diego, CA: Academic Press; 1990:29-49.

Sanders AE, Barnes LG: **Paleontology of the Late Oligocene Ashley and Chandler Bridge Formations of South Carolina, 3: Eomysticetidae, a new family of Oligocene mysticetes (Mammalia: Cetacea).** In *Cenozoic mammals of land and sea, tributes to the career of Clayton E. Ray*. Edited by Emry RE. *Smithson Contrib Paleobiology* 2002a, **93**:313-356.

Sanders AE, Barnes LG: **Paleontology of the Late Oligocene Ashley and Chandler Bridge Formations of South Carolina: *Micromysticetus rothauseni*, a primitive cetotheriid mysticete (Mammalia: Cetacea).** In *Cenozoic mammals of land and sea, tributes to the career of Clayton E. Ray*. Edited by Emry RE. *Smithson Contrib Paleobiology* 2002b, **93**:271-294.

Schulte H von W: **The skull of *Kogia breviceps* Blainv**. *Bull Am Mus Nat Hist* 1917, **37**:361-404.

True FW: **The whalebone whales of the North Atlantic compared with those occuring in European waters with some observations on the species of the North Pacific**. *Smithsonian Contributions to Knowledge* 1904, **33**: 297-332.

True FW: **An account of the beaked whales of the family Ziphiidae in the collection of the United States National Museum, with remarks on some specimens in other American museums.** *Bull US Natl Mus* 1910, **73**: 1-89.

Whitmore FC, Sanders AE: **Review of the Oligocene Cetacea.** *Syst Zool* 1977, **25**:304-320.

Wolman AA: **Gray whale *Eschrichtius robustus* (Lilljeborg, 1861).** In *Handbook of Marine Mammals, Volume 3*. Edited by Ridgway SH, Harrison, R. New York: Academic Press; 1985:67-90.

Yablokov AV: **Covergence or parallelism in the evolution of cetaceans**. *Int Geol Rev* 1964, **7**:1461-1468.

Zeigler CV, Chan GL, Barnes LG: **A new late Miocene balaenopterid whale (Cetacea: Mysticeti), *Parabalaenoptera baulinensis*; (new genus and species) from the Santa Cruz Mudstone, Point Reyes Peninsula, California**. *Proc Calif Acad Sci* 1997, **50**: 115-138.

Zhou, K: **Classification and phylogeny of the Superfamily Platanistoidea, with notes on evidence of the monophyly of the Cetacea.** *Sci Rep Whales Res Inst* 1982, **34**:93-108.

**Unedited Taxon Comments and Key for Character Comments, Generated from WinnClada Data File**

*Kentriodon pernix* : In July 2007, went back over this. It seems that I previously included some skulls that may represent a new species. Revised codings to be based on two specimens: specimen referred to this species by Kellogg (USNM 10670, referred to as A) and the type (USNM 8060, referred to as B). The type is in a plaster mount, which makes several codings difficult. Unless specified, all codings are based on A only, except for the petrotympanics and mandibles, which are based on B, the type.

*Leucopleurus acutus* : E= USNM 14243, F = USNM 14279, G = USNM 504196, H = USNM 571390, I= USNM 484916, J= USNM 571447. Each of these has a skull. F, G, and H have petrosals, although in H the tympanic covers most structures on the ventral side. Tympanics are in F and H only. Mandibles are in G and H only. These apply to characters higher than 328 unless otherwise stated. Skeletal features are based on H and I. J is used too but H and I take precedence because J is not an adult. E through H used to check codings based on GCM 936 through character 200.

*Grampus griseus* : E= USNM 550308, F = USNM 550393 (includes skeleton with scapula but no limbs), G = USNM 550437 (includes skeleton with scapula but no limbs), H = USNM 550936; I= USNM 24224 (used for number of digits, forelimb characters but not scapula); J= USNM 504328 (used for skeletal features only). Petrosal observations based on all three, but ventral side of petrosal only visible in F (right side), G (right side), and H (both sides).

*Orcaella brevirostris* : Based initially on published literature. Checked and modified codings on USNM specimens. A= USNM 199743 (skull, dentaries wo teeth and earbones), B= USNM 284429 (partial skull), C= USNM 486170 (immature skull including dentaries and earbones). Did not look at c except for some earbone features and immature skull features.

*Pseudorca crassidens* : A= USNM 11320 skull, no jaws, some teeth, petrotympanics (right side separated, left are still connected but bulla broken revealing some of ventral surface); B= USNM 484982 skull, jaws, no petrotympanics; C= USNM 218360 right separated petrosal and bulla; D= USNM 501200 skull, jaws. left petrotympanic joined and right petrosal. E= USNM 20932 (looked at skeleton only, includes most of the vertebral column), F= USNM 23283 (looked at skeleton only, includes most of vertebral column, left scapula and forelimb, but not manus); G= USNM 218360 (looked at sternum only).

*Platanista gangetica* : A=USNM 23456. B = USNM 172409 (left petrotympanic and righ tympanic).

*Orcinus orca* : Initially based on ChM 433, but then check based on larger sample size, which is available at the USNM. B= USNM 16488, C= USNM 11980, D= USNM 238112, E= USNM 239351 (looked and mandible only); F= USNM 22068 (looked at joined, right petrotympanic only); G= USNM 219326 (looked at separate right petrosal and tympanic); H= 49909 (looked at right petrosal only); I= USNM 504925 (joined right petrotympanic, tympanic broken to reveal most of petrosal ventral surface); J= USNM 23004 (looked at skeleton only, does not include humerus and more distal forelimb elements); K= USNM 37166 (looked at cervical complex only); L= USNM 219326 (juvenile with skull, was the only specimen examined that still retained the nasals, only examined for a few characters, which are specifically mentioned); M= USNM 294406 (worn and abraded skull, used for features of the occiput not visible on skulls in carriages); N= USNM 571360 (looked a forelimb only, mainly humerus). Mandibular characters observed in C and E.

*Globicephala macrorhynchus* : Originally based on ChM 412, then checked codings with more specimens at USNM. A=USNM 22571 (skull with jaw and separate petrotympanics), B= USNM 22572 (skull and jaw), C= USNM 37261 (skull and jaw, skeleton), D= USNM 550797 (looked at right petrosal and tympanic only, separated from each other); E= USNM 22570 (looked at skeleton only); F= USNM 500213 (looked at skeleton only).

*Phocoenoides dalli* : A= AMNH 90802, B = AMNH 12103, C= GCM 349, D= USNM 504969, E= USNM 396304

*Pontoporia blainvillei* : I= USNM 49432 skull and postcrania but petrotympanics still attached to skull, J= USNM 49442; K = USNM 48254 skull and separate petrotympanics; L = USNM 482747 skull and separate left petrotympanic but joined right; M = USNM 482733 skull and separate petrotympanics.

*Phocoena phocoena* : M=USNM 218739, N= USNM 218738 (used M and N for a few postcranial characters. Speficially noted for each character when these specimens examined). O = USNM 572018 (skull, jaw, separate petrotympanics); P = USNM 572174 (skull, jaw, separate petrotympanics); Q = 572297 (skull, jaw, separate petrotympanics); R = USNM 572300 (skull, jaw, separate petrotympanics).

*Tasmacetus shepherdi* : A = USNM 484878.

*Inia geoffrensis* : A= AMNH 93412, B= AMNH 93414, C= AMNH 93415, D= AMNH 209101, E= AMNH 209103 (each is a skull with lower jaws), I= 93413 (postcranial only), J = USNM 395415 (joined petrotympanics

*Delphinus delphis* : G = USNM 550228 skull with petrotympanics separate; H = USNM 550240 skull with petrotympanics separate; I = USNM 550305 skull right petrtympanic separate left is joined.

*Delphinapterus leucas* : H= USNM 16485 (skull jaws, petrotymapanics); I - USNM 22433 (skull with separate petrotympanics but no jaw); J = USNM 23208 (skull, jaws, petrotympanics still attached to skull); K = USNM 275075 (skull with separate petrotympanics and fragmentary jaws).

*Brachydelphis* holotype : MNHN PPI 121, Holotype. Includes partial skull, both petrosals, and partial tympanic bullae.

*Brachydelphis* 124 : MNHN PPI 124. Fragmentary skull including nearly all of the rostrum, much of the area around the internal nares, and anterior part of basicranial stem. Dorsal surface is highly corroded.

*Kentriodon pernix* : Fragmentary skeleton. Includes left petrosal and left posterior process of tympanic, some vertebrae, both humeri and partial ulna and part of left scapula that has glenoid fossa.

*Brachydelphis* uncat : Nearly complete, but not totally prepared skull. The ventral side in particular is not prepared. No #.

*Pliopontos littoralis* 953 : SAS 953. Includes partial skeleton with skull and right petrotympanics (separate). The rostrum on the skull is incomplete.

*Pliopontos* 193 : *Pliopontos*_*littoralis*. SAS 193. Partial skull.

*Pliopontos* 931 : *Pliopontos*_*littoralis* holotype. SAS 931. Partial skull with right bulla.

*Ninoziphius platyrostris* : MNHN SAS 941. Partial skull and skeleton including both petrosals and a right tympanic.

*Brachydelphis*_125 : Fragmentary skeleton. Includes left petrosal and left posterior process of tympanic, some vertebrae, both humeri and partial ulna and part of left scapula that has glenoid fossa.

*Brachydelphis*_230 : MNHN PPI 230. Very fragmentary skeleton. Includes most of left petrosal, both humeri, left radius and ulna, proximal end of right radius.

**Unedited Taxon Comments and Key for Character Comments, Generated from WinnClada Data File**

*Kentriodon pernix* : In July 2007, went back over this. It seems that I previously included some skulls that may represent a new species. Revised codings to be based on two specimens: specimen referred to this species by Kellogg (USNM 10670, referred to as A) and the type (USNM 8060, referred to as B). The type is in a plaster mount, which makes several codings difficult. Unless specified, all codings are based on A only, except for the petrotympanics and mandibles, which are based on B, the type.

*Leucopleurus acutus* : E= USNM 14243, F = USNM 14279, G = USNM 504196, H = USNM 571390, I= USNM 484916, J= USNM 571447. Each of these has a skull. F, G, and H have petrosals, although in H the tympanic covers most structures on the ventral side. Tympanics are in F and H only. Mandibles are in G and H only. Skeletal features are based on H and I. J is used too but H and I take precedence because J is not an adult. E through H used to check codings based on GCM 936 through character 200.

*Grampus griseus* : E= USNM 550308, F = USNM 550393 (includes skeleton with scapula but no limbs), G = USNM 550437 (includes skeleton with scapula but no limbs), H = USNM 550936; I= USNM 24224 (used for number of digits, forelimb characters but not scapula); J= USNM 504328 (used for skeletal features only). Petrosal observations based on all three, but ventral side of petrosal only visible in F (right side), G (right side), and H (both sides).

*Orcaella brevirostris* : Based initially on published literature. Checked and modified codings on USNM specimens. A= USNM 199743 (skull, dentaries wo teeth and earbones), B= USNM 284429 (partial skull), C= USNM 486170 (immature skull including dentaries and earbones). Did not look at C except for some earbone features and immature skull features.

*Pseudorca crassidens* : A= USNM 11320 skull, no jaws, some teeth, petrotympanics (right side separated, left are still connected but bulla broken revealing some of ventral surface); B= USNM 484982 skull, jaws, no petrotympanics; C= USNM 218360 right separated petrosal and bulla; D= USNM 501200 skull, jaws, left petrotympanic joined and right petrosal. E= USNM 20932 (looked at skeleton only, includes most of the vertebral column), F= USNM 23283 (looked at skeleton only, includes most of vertebral column, left scapula and forelimb, but not manus); G= USNM 218360 (looked at sternum only).

*Platanista gangetica* : A=USNM 23456. B = USNM 172409 (left petrotympanic and righ tympanic).

*Orcinus orca* : Initially based on ChM 433, but then check based on larger sample size, which is available at the USNM. B= USNM 16488, C= USNM 11980, D= USNM 238112, E= USNM 239351 (looked and mandible only); F= USNM 22068 (looked at joined, right petrotympanic only); G= USNM 219326 (looked at separate right petrosal and tympanic); H= 49909 (looked at right petrosal only); I= USNM 504925 (joined right petrotympanic, tympanic broken to reveal most of petrosal ventral surface); J= USNM 23004 (looked at skeleton only, does not include humerus and more distal forelimb elements); K= USNM 37166 (looked at cervical complex only); L= USNM 219326 (juvenile with skull, was the only specimen examined that still retained the nasals, only examined for a few characters, which are specifically mentioned); M= USNM 294406 (worn and abraded skull, used for features of the occiput not visible on skulls in carriages); N= USNM 571360 (looked a forelimb only, mainly humerus). Mandibular characters observed in C and E.

*Globicephala macrorhynchus* : Originally based on ChM 412, then checked codings with more specimens at USNM. A=USNM 22571 (skull with jaw and separate petrotympanics), B= USNM 22572 (skull and jaw), C= USNM 37261 (skull and jaw, skeleton), D= USNM 550797 (looked at right petrosal and tympanic only, separated from each other); E= USNM 22570 (looked at skeleton only); F= USNM 500213 (looked at skeleton only).

*Phocoenoides dalli* : A= AMNH 90802, B = AMNH 12103, C= GCM 349, D= USNM 504969, E= USNM 396304

*Pontoporia blainvillei* : I= USNM 49432 skull and postcrania but petrotympanics still attached to skull, J= USNM 49442; K = USNM 48254 skull and separate petrotympanics; L = USNM 482747 skull and separate left petrotympanic but joined right; M = USNM 482733 skull and separate petrotympanics.

*Phocoena phocoena* : M=USNM 218739, N= USNM 218738 (used M and N for a few postcranial characters. Speficially noted for each character when these specimens examined). O = USNM 572018 (skull, jaw, separate petrotympanics); P = USNM 572174 (skull, jaw, separate petrotympanics); Q = 572297 (skull, jaw, separate petrotympanics); R = USNM 572300 (skull, jaw, separate petrotympanics).

*Tasmacetus shepherdi* : A = USNM 484878.

*Inia geoffrensis* : A= AMNH 93412, B= AMNH 93414, C= AMNH 93415, D= AMNH 209101, E= AMNH 209103 (each is a skull with lower jaws), I= 93413 (postcranial only), J = USNM 395415 (joined petrotympanics

*Delphinus delphis* : G = USNM 550228 skull with petrotympanics separate; H = USNM 550240 skull with petrotympanics separate; I = USNM 550305 skull right petrtympanic separate left is joined.

*Delphinapterus leucas* : H= USNM 16485 (skull jaws, petrotymapanics); I - USNM 22433 (skull with separate petrotympanics but no jaw); J = USNM 23208 (skull, jaws, petrotympanics still attached to skull); K = USNM 275075 (skull with separate petrotympanics and fragmentary jaws).

*Brachydelphis* holotype : MNHN PPI 121, Holotype. Includes partial skull, both petrosals, and partial tympanic bullae.

*Brachydelphis* 124 : MNHN PPI 124. Fragmentary skull including nearly all of the rostrum, much of the area around the internal nares, and anterior part of basicranial stem. Dorsal surface is highly corroded.

*Kentriodon pernix* : Fragmentary skeleton. Includes left petrosal and left posterior process of tympanic, some vertebrae, both humeri and partial ulna and part of left scapula that has glenoid fossa.

*Brachydelphis* uncat : Nearly complete, but not totally prepared skull. The ventral side in particular is not prepared. No #.

*Pliopontos littoralis* 953 : SAS 953. Includes partial skeleton with skull and right petrotympanics (separate). The rostrum on the skull is incomplete.

*Pliopontos* 193 : *Pliopontos*_*littoralis*. SAS 193. Partial skull.

*Pliopontos* 931 : *Pliopontos*_*littoralis* holotype. SAS 931. Partial skull with right bulla.

*Ninoziphius platyrostris* : MNHN SAS 941. Partial skull and skeleton including both petrosals and a right tympanic.

*Brachydelphis*_125 : Fragmentary skeleton. Includes left petrosal and left posterior process of tympanic, some vertebrae, both humeri and partial ulna and part of left scapula that has glenoid fossa.

*Brachydelphis*_230 : MNHN PPI 230. Very fragmentary skeleton. Includes most of left petrosal, both humeri, left radius and ulna, proximal end of right radius.

**Unedited Character Comments Generated from WinClada Data File. Sorted first by character and then by taxon. Letters refer to individual specimens; see taxon comments above for key.**

**Character** 2 :

*Kentriodon pernix* : A and B

*Brachydelphis* holotype : Inferred, even though anterior end of rostrum is broken.

*Pliopontos* 193 : Inferred although rostrum incomplete.

**Character** 4 :

*Globicephala macrorhynchus* : Originally coded as 1but changed to 0 based on USNM specimens. Although the rostrum is flattened, the edge is abrupt and forms about a 90 degree angle with the palate.

**Character** 5 :

*Orcaella brevirostris* : Mesethmoid has filled in posterior third of canal. Not like ziphiid condition but coded the same here.

**Character** 6 :

*Pliopontos* 193 : Approaches state 1.

**Character** 8 :

*Pseudorca crassidens* : Same width in B but widens slightly in A and D.

*Orcinus orca* : True polymorphism. State 0 in B but state 1 in C and D. State 1 best developed in D.

*Brachydelphis* uncat : Narrow.

**Character** 9 :

*Globicephala macrorhynchus* : In A and B, for a short distance there is sporadic contact between the premaxillae.

**Character** 10 :

*Globicephala macrorhynchus* : State 1 in A, state 2 in B and C.

*Ninoziphius platyrostris* : Ambiguity.

*Kentriodon pernix* : In some areas suture approaches state 3, particularly on left side..

**Character** 11 :

*Orcinus orca* : Approaches state 2 in USNM specimens.

*Brachydelphis* 124 : Ambiguity.

**Character** 15 :

*Kentriodon pernix* : Visible on right side of A. Left side of A is broken.

*Orcaella brevirostris* : This is 0 based on A. Check lit for polymorphism.

*Globicephala macrorhynchus* : 1 in A and B but 0 in C.

*Orcinus orca* : Coded as state 1 although originally coded as 0 based on ChM specimen. Clearly 1 in A and B although less clear in C.

*Brachydelphis* holotype : Left side approaches state 0.

**Character** 17 :

*Globicephala macrorhynchus* : In A there are several foramina on the center of the palate just anterior to the max/palatine suture. These are absent are much fewer in B and C.

**Character** 19 :

*Kentriodon pernix* : A and B

*Leucopleurus acutus* : Changed from original codings. Current coding based on E through H.

**Character** 20 :

*Kentriodon pernix* : Changed to ?.

*Orcaella brevirostris* : Processes are very short and wide.

**Character** 22 :

*Pliopontos littoralis* 953 : Posterior part approaches state 1.

**Character** 24 :

*Kentriodon pernix* : Based on A and B appears to be about 31 teeth in max. Max/premax suture is unclear so there could be a few more.

*Grampus griseus* : Looks like no teeth in upper jaw.

*Orcaella brevirostris* : A= r-8, l-7; B= r- , 16 or 17, l-17. Based on alveoli. There is a big difference in these specimens.

*Pseudorca crassidens* : A= 8 both sides; B= 9 both sides, D= 9 right, 10 left.

*Leucopleurus acutus* : E= 32 right, 32 or 33 left; F=?; G= 31 right, 34 left; H= 34 right, 33 left.

*Orcinus orca* : C= 12 teeth, B and D= 13 teeth.

*Globicephala macrorhynchus* : A= 7 or 8 on both sides, although anterior alveolus is partially filled in or may not be an alveolus at all, making 7 teeth on each side; B=7 on both sides, there may have been an 8th much earlier on in the animals life; C=7 on both sides.

*Lipotes vexillifer* : 31 teeth in AMNH specimen. This is somewhat of a guess because the premax/max suture is fused. There are 34 teeth on each side and I estimate that the first three are all or partially in the premax.

*Inia geoffrensis* : Following based on estimate that first two teeth are in premax. A= 25 right, 26 left; B= 26 right, 25 left; C= 24 both; D= 29 right (counted presumed reabsorbed alveoli, actual teeth and alveoli is less), 30 left; E= 28 right, 27 left.

*Brachydelphis* holotype : Based on right side, at least 16 teeth.

*Brachydelphis* 124 : 20 or 21 teeth based on left side.

*Pliopontos* 193 : 26 alveoli preserved on right side of maxilla. Certainly more but depends on length of rostrum.

*Pliopontos littoralis* 953 : 10 alveoli preservd on left side. Certainly many more teeth originally.

**Character** 26 :

*Grampus griseus* : All tooth characters based on lower teeth since upper teeth are absent. Teeth preserved in E and H.

*Orcinus orca* : Not observed in D.

**Character** 27 :

*Pseudorca crassidens* : B only.

*Globicephala macrorhynchus* : A.

*Ninoziphius platyrostris* : Ambiguity. Based on alveoli.

**Character** 28 :

*Grampus griseus* : Based on 571350, which has one ant tooth in and another out.

*Pseudorca crassidens* : B only.

**Character** 31 :

*Orcinus orca* : Not observed in D.

**Character** 32 :

*Orcinus orca* : Not observed in D.

**Character** 33 :

*Orcinus orca* : Not observed in D.

**Character** 35 :

*Grampus griseus* : Based on 571350, which has one ant tooth in and another out. The anterior tooth is angled slightly anteriorly although mostly vertical.

*Orcaella brevirostris* : Changed from 0 based on alveoli orientation.

*Pseudorca crassidens* : Actually anterodorsally.

*Leucopleurus acutus* : In G and H these teeth are erupted. It seems like they would point anterodorsally but not sure.

*Globicephala macrorhynchus* : A.

*Ninoziphius platyrostris* : Ambiguity, based on alveoli.

**Character** 36 :

*Grampus griseus* : Based on 571350, which has one ant tooth in and another out.

*Globicephala macrorhynchus* : A.

**Character** 37 :

*Grampus griseus* : There are 9 isolated teeth in E and 10 in H, which leads to 5 teeth on each side.

*Orcaella brevirostris* : A has 10 on right and 13 on left. Missing teeth so probably old or pathologic.

S*imocetus* : 11 teeth.

*Pseudorca crassidens* : 9 in B (both sides) but 10 in D (both sides)

*Leucopleurus acutus* : G= 36 (2 unerupted) right, 35 (2 unerupted) left; H= 35 right (1 unerupted), 36 (3 unerupted) left.

*Orcinus orca* : C= 11 teeth, E = 12 teeth.

*Globicephala macrorhynchus* : A= 9 both sides, B=8 both sides; C=7 both sides.

*Lipotes vexillifer* : 34 on right 33 on left.

*Inia geoffrensis* : A= 28 right, 27 left; B= 28 both sides; C= 27 right, 26 left; D= 32 on both; E= 33 on both.

*Bos* : 10 teeth

*Pliopontos littoralis* 953 : Ambiguity. 11 teeth/alveoli preserved on fragment of left ramus. Another fragment shows at least 6 more teeth. Certainly there were many more. At the least double that number.

*Ninoziphius platyrostris* : Estimate about 39 teeth.

**Character** 39 :

*Pliopontos littoralis* 953 : Symphysis is long but length very uncertain.

**Character** 40 :

*Grampus griseus* : True polymorphism. Fused to varying degrees in F, G, and H but not in E. Condition in E shared with other USNM specimens.

*Pseudorca crassidens* : Maybe partially fused.

*Globicephala macrorhynchus* : Suture is still visible but clear areas where both sides are fused to each other.

*Ninoziphius platyrostris* : Not certain, could be sutured.

**Character** 42 :

*Ninoziphius platyrostris* : Approaches state 1.

**Character** 45 :

*Orcaella brevirostris* : Looks elevated.

*Globicephala macrorhynchus* : Changed from 1 to 0 based on USNM specimens.

**Character** 46 :

*Leucopleurus acutus* : Changed from original codings. Current coding based on E through H.

*Globicephala macrorhynchus* : More gradual in C.

**Character** 47 :

*Brachydelphis* holotype : Aligned with top of rostrum.

**Character** 51 :

*Pseudorca crassidens* : In ChM specimen and A have state 1 but state 0 in B and D.

**Character** 53 :

*Brachydelphis* holotype : Probably fused but there are symmetrical "cracks" on both sides that could be sutures.

**Character** 54 :

*Kentriodon pernix* : Changed to ?.

**Character** 56 :

*Orcinus orca* : Not observed in D.

**Character** 57 :

*Kentriodon pernix* : Changed to 2 based on A.

**Character** 58 :

*Grampus griseus* : Formed by maxilla and palatine.

*Pseudorca crassidens* : Palatine only.

*Leucopleurus acutus* : Max and palatine, E through H.

*Orcinus orca* : True polymorphism. B= 0, C= 1 (palatine), D= 0 on right side, 1 on left (max and palatine).

*Globicephala macrorhynchus* : The edge is formed nearly entirely of palatine.

*Simocetus* : Ambiguity.

**Character** 61 :

*Grampus griseus* : Tough call.

*Globicephala macrorhynchus* : Short in A and B but long in C.

**Character** 62 :

*Grampus griseus* : There is an incipient ridge on F. The orbital region in H is heavily pitted. Something wrong with this specimen on this side.

*Pseudorca crassidens* : Not sharp.

**Character** 64 :

*Grampus griseus* : This is complex, particularly since the maxillary and infraorbital foramina are close. In E r= 7, l = 9 (two are tiny); F r= 4, l= 5; G r= 4, l= 4; H r=5, l=5.

*Orcaella brevirostris* : In A there 4 on the right (two tiny and one that looks like two merged foramina) and 3 on left (but another is considered part of the maxillary complex of foramina).

*Simocetus* : Based on left side. Right seems to have 2 maybe more. Matrix filling the foramina makes it hard to say.

*Pseudorca crassidens* : A= r-5, l- 5; B= r-5, l-3 (plus one tiny one and another large one that is here interpreted as a maxillary foramen, not infraorbital); D= r-7 (4 very small), l-3 (there is another large one here interpreted as maxillary).

*Leucopleurus acutus* : E= 4 on right, 6 left; F= 4 on right, 6 on left (1 tiny); G= 5 on right, 4 on left; H= 4 on right, 6 on left (2 tiny).

*Globicephala macrorhynchus* : A= 4 on right (2 of them are tiny), 3 on left; B= 5 on right, 6 on left; C= 3 on right, 4 on left.

*Orcinus orca* : B= 5 on both sides, C = 2 on both sides, D = 5 on right and 4 on left, although left also has an additional tiny foramen.

*Brachydelphis* holotype : There are clearly two foramina on each side... could be more.

*Brachydelphis* uncat : Looks like 2 on each side, although there may be more.

*Pliopontos* 193 : Two on right side at least one foramen on left.

*Pliopontos* 931 : Three foramina on left side (posterior one could be considered an additional maxillary foramen). Right side at least two foramina.

*Pliopontos littoralis* 953 : Three foramina on right side, two or three on left.

**Character** 68 :

*Kentriodon pernix* : Are flat transversely in A.

*Grampus griseus* : Are inflated. Generally sigmoidal transversely, with convex and concave portions.

*Simocetus* : Flat.

*Leucopleurus acutus* : Flat transversely, E-H.

*Brachydelphis* holotype : The surfaces are quite weathered, thus coded as ambiguity.

*Ninoziphius platyrostris* : Not certain because dorsal surface is corroded.

**Character** 69 :

*Simocetus* : I am not sure if the anterior foramen is really homologous to other premaxillary foramina. Breakage also makes this unclear. So coded as one or two foramina.

*Orcinus orca* : B has one foramen on both sides, C has 1 on right and 2 on the left, D has 2 on both sides.

*Ninoziphius platyrostris* : Ambiguity.

**Character** 72 :

*Simocetus* : Very long but does not reach nares because of anterior position of foramen.

*Globicephala macrorhynchus* : Changed from 1 based on USNM specimens.

*Orcinus orca* : Short and shallow in ChM specimen and C but absent in B and D.

*Brachydelphis* holotype : Ambiguity.

**Character** 74 :

*Kentriodon pernix* : Changed from original coding. Based on right side of A. At level just posterior to postorbital process.

*Grampus griseus* : In all USNM specimens, the right premax is a little behind the ant edge of the squamosal floor.

*Simocetus* : In line with postorbital process.

*Pseudorca crassidens* : Aligned with anterior third of zygoma in A, B, and D.

*Leucopleurus acutus* : Changed from original coding to 4 based on E to H. In E could also be coded as state 3.

*Globicephala macrorhynchus* : Changed from 4 to 5 based on USNM specimens.

*Brachydelphis* holotype : Close to state 3, aligned with anterior part of zygomatic process.

*Pliopontos* 193 : On right side the right premax might be just ever so slightly behind the ant edge of the squamosal floor.

*Pliopontos littoralis* 953 : Ambiguity, ant edge of squamosal floor is broken.

*Ninoziphius platyrostris* : Ambiguity.

**Character** 75 :

*Kentriodon pernix* : In A could be a smaller 2nd on the right side but not on the left.

*Grampus griseus* : True polymorphism. E = 1, F = 1 on right but 2 on left, G = 1 on right but 3 on left, H = 2 on both sides.

*Orcaella brevirostris* : Within individual polymorphism. In A, 1 on right side but two on left.

*Pseudorca crassidens* : One maxillary foramen in A, one on the right side of B and D but two on the left side of B and D.

*Leucopleurus acutus* : Changed from original coding (which was 2 foramina) to 1 foramen based on E-H.

*Globicephala macrorhynchus* : Two in A and B but 1 in C.

*Orcinus orca* : Left side of D has 2 maxillary foramen. Thus polymorphic 1 and 2 is an alternative coding.

*Brachydelphis* holotype : In anterior position, could be considered part of the infraorbital complex.

*Brachydelphis* uncat : In anterior position.

*Ninoziphius platyrostris* : Ambiguity

**Character** 76 :

*Simocetus* : Does not cover lateral side of frontal. Fordyce, in original description, interpreted this as broken.

**Character** 77 :

*Ninoziphius platyrostris* : Ambiguity.

**Character** 78 :

*Pseudorca crassidens* : Thin in B and ChM specimen. Thick in A and D.

*Globicephala macrorhynchus* : Thick in A but same thickness in B and C.

*Brachydelphis* uncat : Although extreme anterior corner is thickened the rest of the anterolateral margin is not.

**Character** 79 :

*Grampus griseus* : The ridge is weak in G but well developed in other USNM specimens.

*Orcinus orca* : State 0 in D and state 1 in B and C.

*Brachydelphis* holotype : Present but low.

*Brachydelphis* uncat : Approaches state 1.

*Pliopontos* 931 : Approaches state 0.

**Character** 80 :

*Kentriodon pernix* : Changed from original coding. In A. amterior edge of nasals in line with postorbital process of frontal.

*Leucopleurus acutus* : Aligned with postorbital process.

*Globicephala macrorhynchus* : In line with ant edge of squamosal floor.

*Orcinus orca* : Based on ChM specimen.

*Brachydelphis* 124 : Ambiguity, inferred from position of antorbital notch.

*Simocetus* : Polymorphism.

**Character** 82 :

*Kentriodon pernix* : Changed from original coding to 0. I see no evidence of maxilla exposed in this area. Maybe based on one of the other specimens now excluded.

*Orcaella brevirostris* : True polymorphism. State 1 in A but 2 in B.

*Pseudorca crassidens* : State 1 in A and D but state 2 in B.

*Orcinus orca* : State 1 in ChM specimen and B but state 2 is in C and D.

*Brachydelphis* holotype : Not certain.

**Character** 84 :

*Grampus griseus* : The right premax is just slightly post to the left in G, but in rest of USNM specimens examined it is clearly more posterior.

*Orcaella brevirostris* : Not by much.

*Brachydelphis* holotype : Based on sutural surface.

**Character** 87 :

*Orcaella brevirostris* : Coded as zero but in fact right is about 75% the size of the left.

**Character** 89 :

*Grampus griseus* : True polymorphism. State 0 in G and state 1 in E,F, H. State 0 also seen in some other USNM specimens, but did not get numbers.

*Leucopleurus acutus* : True polymorphism. Adjacent to nasal opening in G but not in E, F, and H.

*Orcinus orca* : Originally coded as 1 based on ChM specimen. May need to check for polymorphism in this character.

**Character** 93 :

*Orcinus orca* : Dorsal end of plate still divided by a ridge.

**Character** 107 :

*Kentriodon pernix* : The inflection is absent but the place where it used to be is visible. Based on that next character is coded.

*Grampus griseus* : Inflection absent.

*Orcaella brevirostris* : No inflection and posteromedial splint.

*Pseudorca crassidens* : Inflection absent and premax not divided into a posteromedial splint and posterolateral plate.

*Leucopleurus acutus* : Premax not split into two posteior parts.

*Globicephala macrorhynchus* : Premaxilla not divided.

*Brachydelphis* holotype : No inflection.

*Brachydelphis* uncat : No inflection.

*Pliopontos* 193 : No inflection.

*Pliopontos* 931 : No inflection.

*Pliopontos littoralis* 953 : No inflection.

**Character** 108 :

*Kentriodon pernix* : In line with postorbital process.

**Character** 109 :

*Simocetus* : This is a tough call. *Simocetus* has a pronounced cleft but it originates about 2 cm anterior to where the posteromedial splint and anterolateral plate join. This is my best guess.

**Character** 110 :

*Grampus griseus* : This is complex. On all USNM specimens examined, the left premax overhangs slightly while the right does not, except in H.

*Orcaella brevirostris* : Right premax does not but left does.

*Pseudorca crassidens* : Just anterior to nasal opening, the right premax slightly overhangs in A and D, the left does in A only, and neither side does in B.

*Globicephala macrorhynchus* : C approaches state 0.

*Orcinus orca* : True polymorphism. State 0 in C and D, state 1 in B.

**Character** 111 :

*Kentriodon pernix* : Changed to ? from original coding. Presumably based on one of the excluded skulls.

*Grampus griseus* : There is a foramen in this region but based on a juv/neonate USNM 550348, this foramen is continuous with a canal that leads into the infraorbital/maxillary complex.

*Orcaella brevirostris* : C only.

**Character** 112 :

*Grampus griseus* : Subtle but should go with state 0, anterolateral. Least developed in H.

*Leucopleurus acutus* : Changed from 0 to 1 based on E-H.

*Orcinus orca* : Approaches state 0.

*Pliopontos* 193 : Much better developed on left side. Right side closer to state 1.

*Pliopontos* 931 : Much better developed on left side. Right side closer to state 1.

*Pliopontos littoralis* 953 : Much better developed on left side. Right side closer to state 1.

**Character** 113 :

*Orcinus orca* : L only.

**Character** 114 :

*Orcinus orca* : L only.

*Brachydelphis* uncat : The braincase is slightly crushed, complicating this coding, but I am fairly sure the nasal suture is not shifted to the left, or if so, it is quite minor. Overall the face itself in posterior view seems to be facing slightly to the left, thus being rotated counterclockwise. Seems to be the same in the holotype, although this could be due to distortion.

**Character** 116 :

*Orcinus orca* : L only.

*Pliopontos* 193 : Approaches state 0.

*Pliopontos* 931 : Approaches state 1.

**Character** 117 :

*Orcinus orca* : L only.

**Character** 118 :

*Globicephala macrorhynchus* : In A, medial portions are slightly lower but certainly no trough.

*Orcinus orca* : L only.

*Kentriodon pernix* : Polymorphism

**Character** 121 :

*Grampus griseus* : The left frontal has a prominent process that wedges between the nasals.

*Orcinus orca* : L only.

*Brachydelphis* uncat : There is a deep cleft, but no apparent processes of frontals.

**Character** 122 :

*Kentriodon pernix* : In A aligned with ant edge of squa fossa floor.

*Pseudorca crassidens* : In line with anterior edge of squamosal floor.

*Brachydelphis* holotype : Just anterior to edge of squamosal floor.

*Brachydelphis* uncat : In line with anterior edge of squamosal floor.

*Simocetus* : Polymorphism

**Character** 124 :

*Grampus griseus* : Nasals are very slightly higher than the frontals.

*Brachydelphis* uncat : Approaches state 0.

**Character** 125 :

*Orcaella brevirostris* : Seem to be the same width in A and B.

**Character** 126 :

*Grampus griseus* : Although the frontals are not nodular, they have a prominent cleft/open suture separating them.

*Orcaella brevirostris* : Clear open suture in A and B.

*Pseudorca crassidens* : The suture is quite open in A.

**Character** 127 :

*Kentriodon pernix* : Changed to ?. Presumably was based on one of the excluded skulls.

*Orcinus orca* : L only.

**Character** 128 :

*Kentriodon pernix* : Changed original coding from 2 to 1. Based on A supraoccipital is at same level maybe even a little lower.

*Grampus griseus* : Subtle, supraoccipital is really close to nasals in height.

*Pseudorca crassidens* : Slightly below in A and D but same level in B.

*Leucopleurus acutus* : True polymorphism. Same height in G but lower in E, F, and H.

*Orcinus orca* : Changed from 1 to 0 based on USNM specimens. Original coding based on ChM skull, which was somewhat worn.

*Brachydelphis* uncat : Approaches state 1.

**Character** 130 :

*Grampus griseus* : Lateral edge is upturned but otherwise fits state 0 best.

*Pseudorca crassidens* : Edge of crest is upturned in D.

**Character** 132 :

*Simocetus* : Actually roof is quite small.

*Orcinus orca* : True polymorphism. Maxilla exposed in ChM specimen and B but not in C and D.

**Character** 133 :

*Kentriodon pernix* : Changed to ?.

*Brachydelphis* holotype : Angled anteroventrally.

*Pliopontos* 193 : Slightly anteroventral.

*Pliopontos* 931 : Slightly anteroventral.

**Character** 134 :

*Grampus griseus* : In USNM 550982 (a juv) the parietals are large and articulate via an irregular suture.

*Orcaella brevirostris* : C only.

*Pseudorca crassidens* : No immatures available, that is why coding is ambiguous.

*Leucopleurus acutus* : Based on USNM 504178, a juvenile skull.

*Brachydelphis* uncat : Ambiguity.

*Pliopontos* 193 : Ambiguity.

*Pliopontos* 931 : Ambiguity.

*Pliopontos littoralis* 953 : Ambiguity.

**Character** 135 :

*Kentriodon pernix* : Changed to ?.

*Grampus griseus* : Based on USNM 550982 (a juv).

*Pliopontos* 931 : Not certain.

**Character** 136 :

*Pseudorca crassidens* : Not really the case in D since the maxillary edge is upturned.

*Pliopontos* 193 : Not really.

**Character** 138 :

*Kentriodon pernix* : Changed to ?.

**Character** 139 :

*Kentriodon pernix* : Based on A changed from 1 to 0. In A, supraoccipital is close to but behind ant edge of squa fossa floor.

*Simocetus* : Close but looks a little anterior to the anterior edge of the squamosal floor.

*Brachydelphis* holotype : Approaches state 1, could be that state if supraoccipital is broken.

*Brachydelphis* uncat : Just posterior to edge of squamosal floor.

*Simocetus* : Ambiguity.

**Character** 141 :

*Pliopontos* 193 : Does not have broad temporal exposure.

*Ninoziphius platyrostris* : The subtemporal crest is well developed but this does not mean it has a bulge.

**Character** 142 :

*Leucopleurus acutus* : Too short.

**Character** 143 :

*Kentriodon pernix* : Probably have some polymorphism here. In B, there is a distinct flange, but flange is absent in A. In this taxon zygoma is inclined anterodorsally with a flat to slightly concave dorsal margin.

*Pseudorca crassidens* : Actually sort of sigmoidal with a posterior convex part, then a swell that curves slightly anterodorsally.

*Brachydelphis* holotype : Oriented anterodorsally.

*Brachydelphis* uncat : Slightly concave, approaches state 0. The entire process is directed anterodorsally.

*Pliopontos* 193 : Directed anterodorsally.

**Character** 144 :

*Pseudorca crassidens* : Check ChM specimen. In USNM specimens examined, it looks like slight emargination.

*Leucopleurus acutus* : Changed from 2 to 1 based on E-H.

**Character** 147 :

*Globicephala macrorhynchus* : Maybe straight is a better way to describe it. The floor rises up posteriorly but is not sigmoidal.

**Character** 148 :

*Pliopontos* 193 : Not dramatic thickening.

*Pliopontos littoralis* 953 : Not dramatic thickening.

**Character** 150 :

*Leucopleurus acutus* : Slightly concave, nearly flat.

*Globicephala macrorhynchus* : Changed from 0 to 1 based on USNM specimens.

*Brachydelphis* holotype : Ambiguity.

*Pliopontos* 193 : Actually sigmoidal, convex anteriorly but concave posteriorly.

*Pliopontos littoralis* 953 : Actually sigmoidal, convex anteriorly but concave posteriorly.

**Character** 151 :

*Grampus griseus* : Sort of ambiguous in USNM specimens.

*Leucopleurus acutus* : True polymorphism. In E-G blunt but in H comes to a point.

*Ninoziphius platyrostris* : But differs from eurhinodelphid condition in that it is not blocky.

**Character** 152 :

*Orcaella brevirostris* : True polymorphism. Round in A box like in B.

*Orcinus orca* : M only.

*Brachydelphis* holotype : Ambiguity.

*Pliopontos* 193 : Does have median point but overall rectangular. Same in all three specimens.

**Character** 153 :

*Leucopleurus acutus* : Changed from 1 based on E-H. Was state 2.

*Pliopontos* 931 : Ambiguity.

*Pliopontos littoralis* 953 : Ambiguity.

**Character** 154 :

*Kentriodon pernix* : Changed to ?.

**Character** 155 :

*Grampus griseus* : Development varies. Best developed in E and F.

*Orcaella brevirostris* : Crest is low and indistinct.

*Pseudorca crassidens* : Crest is very weak in D but clear in A and B.

*Leucopleurus acutus* : There is a tiny and very short sagittal crest in F.

*Globicephala macrorhynchus* : Changed from 0 to 1. The crest is much better developed in A and B (which are probably males) than C.

*Orcinus orca* : M only.

*Pliopontos* 931 : There is a very small crest at the dorsal end but this is absent on nearly all of the supraoccipital, thus coded 0.

**Character** 157 :

*Grampus griseus* : Does seem to excavate into the maxilla, particularly in F and H but is short not long.

*Brachydelphis* holotype : Short fossa that extends slightly anterior to jugal.

*Brachydelphis* 124 : Short fossa.

*Pliopontos* 193 : No clear fossa.

*Pliopontos littoralis* 953 : No clear fossa.

*Ninoziphius platyrostris* : Ambiguity. No fossa.

**Character** 159 :

*Orcaella brevirostris* : Current coding based on A.

*Globicephala macrorhynchus* : In A and C the pterygoid coveres all of the medial exposure of the palatine leaving just the lateral lamina exposed.

**Character** 160 :

*Ninoziphius platyrostris* : Very shallow fossa.

**Character** 161 :

*Kentriodon pernix* : Changed to 0. I can see the max/palatine suture in A

**Character** 163 :

*Orcaella brevirostris* : Maybe a little anterior to orbit but not by much.

*Brachydelphis* 124 : Ambiguity.

*Pliopontos* 931 : Ambiguity.

**Character** 164 :

*Orcinus orca* : Not unreasonable to code as 2, absent entirely.

*Brachydelphis* 124 : Could be broken so extent is uncertain. Does seem more extensive than in most Pliocene odontocetes.

*Brachydelphis* uncat : It is possible that the lateral lamina extended more posteriorly but it seems to be ending in the same in place 2 specimens (this one and 124)

**Character** 165 :

*Ninoziphius platyrostris* : Very well developed.

**Character** 167 :

*Kentriodon pernix* : Changed from 2 or 3 to 3 based on A.

*Brachydelphis* holotype : Ambiguity.

*Ninoziphius platyrostris* : Approaches state 0 but most of alisphenoid exposed. It almost seems like early in ontogeny pterygoid covers the alisphenoid but as the sinus develops, it is reabsorbed exposing the alisphenoid.

**Character** 169 :

*Leucopleurus acutus* : Getting close to state 4 but I would code as 3.

*Pliopontos* 931 : Ambiguity.

*Pliopontos* 193 : Approaches state 4.

*Pliopontos littoralis* 953 : Approaches state 4.

**Character** 170 :

*Kentriodon pernix* : There is an excavation but the fossa is very small in A. *Orcaella brevirostris* : Looks like there is a postorbital fossa in B.

*Globicephala macrorhynchus* : Changed from 3 to 2 based on USNM specimens.

*Brachydelphis* holotype : Ambiguity. No clear fossa present.

**Character** 172 :

*Pseudorca crassidens* : Are hollowed out, like state 2 but the ends are plate-like.

*Leucopleurus acutus* : They are hollowed out laterally but most of it is a plate.

*Globicephala macrorhynchus* : Changed from 2 to 3 based on USNM specimens. It is plate-like medially, although thick. Laterally it is hollowed out.

*Brachydelphis* 124 : Is hollowed out latreally but medial portion is a plate.

*Ninoziphius platyrostris* : Posterior end approaches state 1.

**Character** 173 :

*Grampus griseus* : Keels are very weak in E and F.

*Orcaella brevirostris* : Keels look really weak in A.

**Character** 174 :

*Grampus griseus* : Really between states 0 and 1 but closest to state 1.

*Leucopleurus acutus* : Actually over posterior half of orbit but closest to middle of orbit.

*Globicephala macrorhynchus* : Changed from 2 to 1 based on USNM specimens. The hamular processes are approximately in line with the postorbital processes.

*Brachydelphis* 124 : Ambiguity.

*Ninoziphius platyrostris* : Approaches state 2.

**Character** 175 :

*Globicephala macrorhynchus* : True polymorphism. There is a shallow fossa in A but none in B or C.

**Character** 176 :

*Kentriodon pernix* : Changed to ?. Could be broken.

*Globicephala macrorhynchus* : Based on A and B.

*Ninoziphius platyrostris* : Not entirely preserved but looks like state 1.

**Character** 184 :

*Grampus griseus* : True polymorphism and varies a lot. There is a clear ridge in G and in the ChM specimen examined. the ridge is absent in E, F, and H.

*Pseudorca crassidens* : A and D.

*Leucopleurus acutus* : Hard to say. Looks like a ridge in G but not in E, F, and H. Code as polymorphic.

*Platanista gangetica* : A

**Character** 185 :

*Grampus griseus* : In F and to a lesser extent in G there is an excavation into the squamosal but this is in a more anterior position, really lateral to where the anterior process of the petrosal would lie.

*Orcaella brevirostris* : Probably not homologous because in a slightly more dorsal position than in platanistoids and xenorophoids.

*Pseudorca crassidens* : Absent but there is an excavation dorsolateral to the anterior process. Same thing seen in *Grampus*.

*Leucopleurus acutus* : rue polymorphism. Shallow in F but deep in E, G, and H.

*Orcinus orca* : True polymorphism. C= 0, B and D = 1.

*Platanista gangetica* : A

**Character** 186 :

*Grampus griseus* : This area is complex but I could not find a clear foramen.

*Globicephala macrorhynchus* : There is a large opening in C but not sure if it is just a gap in the suture or a true foramen.

*Platanista gangetica* : A

**Character** 187 :

*Grampus griseus* : True polymorphism. State 0 is in F and G, state 3 is in G and H.

*Leucopleurus acutus* : Polymorphism. State 3 in E and G, state 0 in F and H.

*Orcinus orca* : True polymorphism. B = 2, C and D = 0.

**Character** 189 :

*Orcinus orca* : It is wide but not compared to the size of the skull. Relatively it is quite narrow.

**Character** 192 :

*Globicephala macrorhynchus* : Very well developed.

*Brachydelphis* holotype : Part on basioccipital approaches state 1.

**Character** 193 :

*Kentriodon pernix* : Coded as 1 based on A.

*Pliopontos* 931 : Flange but no notch and is more like an undercut ridge.

*Pliopontos* 193 : Flange but no notch and is more like an undercut ridge.

**Character** 196 :

*Globicephala macrorhynchus* : Narrow in A and B but open in C.

*Brachydelphis* holotype : Approaches state 1, but much more open than many foramina.

**Character** 197 :

*Orcinus orca* : In far posterior position compared to most delphinids.

**Character** 200 :

*Leucopleurus acutus* : F and G

**Character** 201 :

*Leucopleurus acutus* : F and G

*Grampus griseus* : True polymorphism. The point when present is at the anterodorsal corner. Point developed in E (left side , G (right side), and H (left side). In others squared off.

**Character** 202 :

*Leucopleurus acutus* : F and G

*Globicephala macrorhynchus* : Deflected in D but not in A.

**Character** 204 :

*Leucopleurus acutus* : F, G, H

*Orcaella brevirostris* : Slightly concave but close enough to flat to code as such.

*Pseudorca crassidens* : Flat to slightly concave.

*Globicephala macrorhynchus* : Slightly concave in A but clearly concave in D.

*Orcinus orca* : In all concave but in H nearly flat, F, clearly concave, and in G and I concave but not as concave as in F.

**Character** 205 :

*Leucopleurus acutus* : F, G, H

*Brachydelphis* holotype : There is a groove but is bowed dorsally.

**Character** 206 :

*Leucopleurus acutus* : F,G,H

*Brachydelphis* holotype : There is a groove here, position seems correct.

*Kentriodon pernix* : There is a hint of a shallow sulcus here.

*Brachydelphis*_125 : There is a hint of a shallow sulcus here.

**Character** 207 :

*Leucopleurus acutus* : Based on F and G

*Pseudorca crassidens* : Based on B, which has petrotympanics still in articulation with the skull.

*Globicephala macrorhynchus* : Petrosal not articulated so unsure. Regardless little to no contact, thus 2 or 3.

**Character** 209 :

*Orcinus orca* : Based on F, G, and I.

*Brachydelphis* holotype : Too short.

*Kentriodon pernix* : Too short

*Pliopontos littoralis* 953 : Too short.

*Brachydelphis*_125 : Too short

*Brachydelphis*_230 : Too short.

**Character** 210 :

*Leucopleurus acutus* : F, G, and H

**Character** 211 :

*Orcinus orca* : The lateral tuberosity actually has a fossa for the sigmoid process. Best developed in G and H. Makes the tubeosity seem smaller.

*Brachydelphis* holotype : Approaches state 0.

*Pliopontos littoralis* 953 : Really absent.

*Ninoziphius platyrostris* : Very well developed.

*Brachydelphis*_230 : Very weak, approaches state 0.

**Character** 212 :

*Orcinus orca* : True polymorphism. Narrow in G, but wide in F, H, and I.

*Pliopontos littoralis* 953 : Hard to code. Looks narrow but edge of posterior process poorly defined.

**Character** 213 :

*Leucopleurus acutus* : Both this and character 214 (malleus fossa) based on F and G.

*Grampus griseus* : Present in F and H but cannot find in G. In G this could be a preservational artifact so coded as present.

*Pseudorca crassidens* : Broken off in A.

*Orcinus orca* : Could not be scored in F.

**Character** 214 :

*Orcinus orca* : Could not be scored in F.

**Character** 216 :

*Leucopleurus acutus* : Both this and next character (tensor tympani groove) are based on F amd G.

*Grampus griseus* : True polymorphism and highly variable. Whole side is pitted in H, both the anterior and posterior processes in G, similar in F except just the dorsal edge of anterior process and the ventral edge of posterior process, and just sporadic spongy bone in F.

*Globicephala macrorhynchus* : Changed from 2 to 3 based on USNM specimens.

*Orcinus orca* : Extreme ventral edge of posterior process contains rugged bone but rest is smooth.

*Ninoziphius platyrostris* : This is difficult. On the right side the petrosal almost approaches state 0. The posterior process is definitely rugose as is the anteriormost point of the anterior process. On the left side the petrosal is not very rugose laterally, but not quite smooth either. State 2 seems like the best compromise coding, although alternatively within individual polymoprhism could be used too.

**Character** 217 :

*Globicephala macrorhynchus* : Changed from 2 to 1 based on USNM specimens.

**Character** 218 :

*Leucopleurus acutus* : True polymorphism.

*Orcinus orca* : Could not be scored in F.

*Ninoziphius platyrostris* : It appears like state 2 (acute) because of the accessory ossicle but if this is removed it is 9 degrees.

**Character** 220 :

*Leucopleurus acutus* : This and character through 329 based on F and G.

*Pliopontos littoralis* 953 : Tough call.

**Character** 221 :

*Orcinus orca* : Could not be scored in F.

**Character** 222 :

*Leucopleurus acutus* : State 0 in F and 1 in G.

*Orcinus orca* : True polymorphism. Tear-drop shape with short fissure in G, tear-drop but no fissure in H, oval in F and I.

\**Character** 223 :

*Pseudorca crassidens* : True polymorphim. A = 0 while C and D =1.

*Pliopontos littoralis* 953 : Approaches state 1.

**Character** 224 :

*Grampus griseus* : True polymorphism. The stylomastoid fossa is absent in E and G, but small and present in F and G.

*Pseudorca crassidens* : Really small in A but well developed in others.

*Kentriodon pernix* : Approaches state 0.

*Pliopontos littoralis* 953 : Very poorly defined. Approaches absent.

*Ninoziphius platyrostris* : Very small.

*Brachydelphis*_125 : Approaches state 0.

**Character** 225 :

*Grampus griseus* : Does not look prominent on left side of H but I think this is broken.

*Orcinus orca* : Not visible in I, caudal tympanic process appears to be broken.

**Character** 226 :

*Ninoziphius platyrostris* : Left petrosal approaches state 1.

**Character** 227 :

*Orcaella brevirostris* : Endolymphatic foramen is huge.

*Globicephala macrorhynchus* : Perilymphatic is smaller in A but subequal in D.

*Orcinus orca* : Could not be scored in H because foramina filled with matrix.

*Ninoziphius platyrostris* : On both petrosals the perilympahic foramen is slightly smaller than the endolympahtic foramen. However, in both the perilymphatic is quite large and close enough in size to the endolympahtic to be considered subequal.

**Character** 231 :

*Ninoziphius platyrostris* : Not certain.

**Character** 234 :

*Leucopleurus acutus* : F, G, H

*Orcinus orca* : Funnel-shaped in all but F.

**Character** 235 :

*Ninoziphius platyrostris* : Unlikely to be homologous to early mysticete condition, but it is certainly very high.

**Character** 236 :

*Leucopleurus acutus* : This through character 241 based on F, G, and H.

*Grampus griseus* : True polymorphism. State 1 in E, G, and H. State 2 is in F and a ChM specimen.

*Pseudorca crassidens* : Nearly equal in D.

*Ninoziphius platyrostris* : Partition separating singular foramen from tractus spiralis is quite high.

**Character** 238 :

*Grampus griseus* : True polymorphism. Circular in E and G and oval in F and H.

*Globicephala macrorhynchus* : Circular in D and A right side, oval in A left side.

*Orcinus orca* : Could not be scored in H. Also in F there appears to be a fissure but this is superficial. The foramen itself is circular.

**Character** 240 :

*Leucopleurus acutus* : Hard to tell either 3 or 4.

*Grampus griseus* : Not sure. My best guess is state 3 or 4.

*Pseudorca crassidens* : Based on B only.

*Globicephala macrorhynchus* : Unsure but either 3 or 4 based on articulating the petrosal by hand.

**Character** 241 :

*Leucopleurus acutus* : F only

*Orcinus orca* : Could not be scored in F and I.

*Brachydelphis* holotype : Slight hint of grooves, so approaches state 1.

*Kentriodon pernix* : Very faint grooves, approaches state 1.

*Brachydelphis*_125 : Very faint grooves, approaches state 1.

**Character** 242 :

*Leucopleurus acutus* : F only

*Pseudorca crassidens* : Approaches flat in A.

*Globicephala macrorhynchus* : Slightly concave but close enough to flat so coded as state 1.

*Orcinus orca* : Could not be scored in F and I.

*Brachydelphis* holotype : Slightly concave.

*Pliopontos littoralis* 953 : Slightly convex.

**Character** 243 :

*Leucopleurus acutus* : F, G, and H

**Character** 244 :

*Leucopleurus acutus* : F, G, H

*Kentriodon pernix* : Long relative to length.

*Brachydelphis*_125 : Long relative to length.

**Character** 250 :

*Grampus griseus* : Very long in G and H. Small in F and probably broken in E. This projection originates almost entirely from the involucrum side and may not be homologous to the spine in other taxa.

*Orcaella brevirostris* : Looks absent.

**Character** 252 :

*Leucopleurus acutus* : F and H

*Pseudorca crassidens* : Does point on right side in C but ignored because state in minority.

*Globicephala macrorhynchus* : Does not protrude much but definitely forms an angle and not rounded over.

*Orcinus orca* : In G does not really point medially but still is not rounded.

*Pliopontos* 931 : Does not point much medially, more ventrally.

**Character** 253 :

*Globicephala macrorhynchus* : A left side.

**Character** 254 :

*Globicephala macrorhynchus* : A, left side only.

*Ninoziphius platyrostris* : Just not sure. It is not obviously state 1 but need to compare with other ziphiids.

**Character** 255 :

*Leucopleurus acutus* : Based on F. In G there posterior end of the ossicle is fused but most is not.

*Grampus griseus* : Posterior portion is partially fused but rest is not.

*Orcinus orca* : Based on G.

**Character** 256 :

*Leucopleurus acutus* : This through char 265 are based on F and H.

*Globicephala macrorhynchus* : A only.

*Orcinus orca* : It is really faint in F and G. Could not be scored in I.

**Character** 258 :

*Leucopleurus acutus* : F only

*Grampus griseus* : H only.

*Pseudorca crassidens* : Based on ChM specimen.

*Globicephala macrorhynchus* : A only.

*Orcinus orca* : G only.

**Character** 259 :

*Grampus griseus* : Ventral margin nearly absent in H, right side.

*Pseudorca crassidens* : Nearly absent.

*Globicephala macrorhynchus* : As seen in A, the ventral margin seems almost absent because it smoothly curves into the lateral side.

*Orcinus orca* : Scaored in F and G. Very well developed.

*Platanista gangetica* : A and B

**Character** 260 :

*Leucopleurus acutus* : H only

*Orcinus orca* : Forms fossa in petrosal.

*Platanista gangetica* : A

*Pliopontos littoralis* 953 : Ambiguity.

*Ninoziphius platyrostris* : Ambiguity.

**Character** 262 :

*Leucopleurus acutus* : Based on F and G.

*Pseudorca crassidens* : Based on B only.

*Globicephala macrorhynchus* : Note certain but best guess.

**Character** 263 :

*Platanista gangetica* : A

*Pliopontos littoralis* 953 : Not certain.

**Character** 264 :

*Grampus griseus* : Hard to say, particularly in F which is thinner than the other specimens.

*Orcinus orca* : Based on G.

**Character** 265 :

*Orcinus orca* : Based on G.

**Character** 267 :

*Leucopleurus acutus* : Based on F and H.

*Platanista gangetica* : A and B

*Ninoziphius platyrostris* : Tough call.

**Character** 268 :

*Brachydelphis* holotype : Not certain but seems to be the case.

**Character** 269 :

*Leucopleurus acutus* : F and H

*Grampus griseus* : Medial side is much more anterior.

*Pliopontos* 931 : Not pronounced.

*Pliopontos littoralis* 953 : Not pronounced.

**Character** 270 :

*Grampus griseus* : Actually diverge.

*Pseudorca crassidens* : Actually diverge

*Globicephala macrorhynchus* : Actually diverge.

*Orcinus orca* : In F and I actually diverge while G is between state 0 and 1.

**Character** 271 :

*Leucopleurus acutus* : F only

*Grampus griseus* : This and next character based on F, G, H but not E.

*Pseudorca crassidens* : A and C.

*Platanista gangetica* : A and B

*Ninoziphius platyrostris* : The groove is actually absent but the steep step in the thickness of the involucrum is present.

**Character** 272 :

*Leucopleurus acutus* : F only

*Pseudorca crassidens* : A and C.

*Orcinus orca* : Could not be scored in F.

*Platanista gangetica* : A and B

**Character** 273 :

*Leucopleurus acutus* : F and H

**Character** 274 :

*Leucopleurus acutus* : This and next character based on F and H.

**Character** 275 :

*Ninoziphius platyrostris* : Posterior third is concave but anterior 2/3rds is convex, so coded as convex.

**Character** 277 :

*Leucopleurus acutus* : Very big.

*Orcinus orca* : J and K.

*Ninoziphius platyrostris* : Dorsal is probably absent.

**Character** 278 :

*Orcinus orca* : J and K.

**Character** 279 :

*Leucopleurus acutus* : Arches are fused, not centra.

*Globicephala macrorhynchus* : C has state 4, E and F have state 5.

*Orcinus orca* : J and K.

**Character** 280 :

*Leucopleurus acutus* : Appears to be true polymorphism. 15 sets of ribs in J, 14 sets in H.

*Pseudorca crassidens* : Based on F, which has 15 ribs total.

*Grampus griseus* : Based on F and J.

*Globicephala macrorhynchus* : Based on ribs, 10 thoracics in C, E, and F.

*Orcinus orca* : Based on 22 ribs (both sides) in J.

*Pliopontos littoralis* 953 : I think the 3rd is missing, possibly the 4th too. There are one or two likely missing from the end. Given that 10 are preserved, this gives a count up to 14 and no less than 12.

**Character** 281 :

*Leucopleurus acutus* : 5 in J and H.

*Pseudorca crassidens* : Based on F and G, which have 11 double-headed and 4 single.

*Grampus griseus* : True polymorphism. There are 6 in J and 7 in F.

*Globicephala macrorhynchus* : In C there are 13 double and 7 single (interpret that one vertebrae differs on each side), E has 12 double and 8 single, F has 10 double and 10 single. There are totals and imclude ribs on both sides.

*Orcinus orca* : Based on J. From both sides, 14 ribs have two heads and 8 have 1.

*Pliopontos littoralis* 953 : Ambiguity. There are 10 double headed ribs and 8 single headed ribs. Divided by 2 for each side, this means 5 vertebrae with 2 articulations and 4 with one. However, there are between 12 and 14 thoracics. This means that there are between 5 and 10 vertebrae with double articulations.

**Character** 282 :

*Leucopleurus acutus* : J only.

*Globicephala macrorhynchus* : C and F only.

*Orcinus orca* : J.

**Character** 283 :

*Leucopleurus acutus* : Really rounded over.

**Character** 284 :

*Orcinus orca* : J.

*Platanista gangetica* : A, slightly ventrolaterally in anterior ones.

**Character** 285 :

*Orcinus orca* : J.

*Platanista gangetica* : A

**Character** 287 :

*Leucopleurus acutus* : 19 lumbars in J,

*Pseudorca crassidens* : 8 or 9 in F. Need to corroborate.

*Grampus griseus* : Some lumbars and caudals are very similar. 17 in F and J.

*Globicephala macrorhynchus* : C= 13 lumbars, F has 14, E has 12.

*Orcinus orca* : 6 lumbars based on J.

*Pliopontos littoralis* 953 : 9 or 10 are preserved but some are missing. True number could be another 5 more.

**Character** 288 :

*Leucopleurus acutus* : In J there are 38 caudals.

*Pseudorca crassidens* : I count 15 or 16 but I do not know if the vertebral column is complete. Need to corroborate coding.

*Grampus griseus* : Hard to say but seem polymorphic. I counted 32 caudals in J and 30 in F.

*Globicephala macrorhynchus* : 24 caudals in C, 28 in E, 23 in F.

*Orcinus orca* : 18 caudals are observed but at least 4 more should be present. Based on J.

**Character** 289 :

*Kentriodon pernix* : Changed to ?.

*Leucopleurus acutus* : They are really small.

*Orcinus orca* : J.

**Character** 290 :

*Kentriodon pernix* : Changed to ?.

*Orcinus orca* : J.

**Character** 291 :

*Kentriodon pernix* : Changed to ?.

*Orcinus orca* : J.

**Character** 292 :

*Orcinus orca* : J.

**Character** 293 :

*Orcinus orca* : J.

**Character** 294 :

*Kentriodon pernix* : Changed to ?.

*Grampus griseus* : Visible in I but not so clear in J.

*Globicephala macrorhynchus* : C and E for this character and those up to 298.

*Orcinus orca* : N.

*Platanista gangetica* : A

**Character** 295 :

*Kentriodon pernix* : Changed to ?.

*Orcinus orca* : N.

*Platanista gangetica* : Not applicable

**Character** 296 :

*Kentriodon pernix* : Changed to ?.

*Orcinus orca* : N.

**Character** 297 :

*Kentriodon pernix* : Changed to ?.

*Grampus griseus* : Subequal to ulna but a little shorter than radius.

*Orcinus orca* : N.

*Brachydelphis*_230 : Longer than ulna subequal to radius.

**Character** 298 :

*Kentriodon pernix* : Changed to ?.

*Orcinus orca* : N.

**Character** 299 :

*Leucopleurus acutus* : Based on I only. Could be wrong since some bones are missing but my best guess is 4.
